# Evaluation of the dependence of radiomic features on the machine learning model

**DOI:** 10.1186/s13244-022-01170-2

**Published:** 2022-02-24

**Authors:** Aydin Demircioğlu

**Affiliations:** Institute of Diagnostic and Interventional Radiology and Neuroradiology, University Hospital Essen, Hufelandstraße 55, 45157 Essen, Germany

**Keywords:** Radiomics, Feature relevance, Biomarkers, Feature selection, Machine learning

## Abstract

**Background:**

In radiomic studies, several models are often trained with different combinations of feature selection methods and classifiers. The features of the best model are usually considered relevant to the problem, and they represent potential biomarkers. Features selected from statistically similarly performing models are generally not studied. To understand the degree to which the selected features of these statistically similar models differ, 14 publicly available datasets, 8 feature selection methods, and 8 classifiers were used in this retrospective study. For each combination of feature selection and classifier, a model was trained, and its performance was measured with AUC-ROC. The best-performing model was compared to other models using a DeLong test. Models that were statistically similar were compared in terms of their selected features.

**Results:**

Approximately 57% of all models analyzed were statistically similar to the best-performing model. Feature selection methods were, in general, relatively unstable (0.58; range 0.35–0.84). The features selected by different models varied largely (0.19; range 0.02–0.42), although the selected features themselves were highly correlated (0.71; range 0.4–0.92).

**Conclusions:**

Feature relevance in radiomics strongly depends on the model used, and statistically similar models will generally identify different features as relevant. Considering features selected by a single model is misleading, and it is often not possible to directly determine whether such features are candidate biomarkers.

**Supplementary Information:**

The online version contains supplementary material available at 10.1186/s13244-022-01170-2.

## Key points

- Different combinations of feature selection methods and classifiers result in models that are not significantly different from the best model in approximately 57%.
- Features selected by statistically best-performing models are largely different (0.19; range 0.02–0.42), although their correlation is higher (0.71; range 0.4–0.92).
- Relevance of features often cannot be decided by the single best model.

## Background

Radiomics is an emergent technique used for diagnostic and predictive purposes and is based on machine learning techniques [1, 2]. It promises a non-invasive, personalized medicine and is applied primarily in an oncological context for diagnosis, survival prediction, and other purposes [3]. Radiomics is often performed using a well-established machine learning pipeline; generic features from the images are first extracted before feature selection methods and classifiers for modeling are employed [4, 5]. One of the main concerns related to radiomics is whether the extracted features have biological meaning [6].

However, in the absence of a direct link between features and the underlying biology of various pathologies, in radiomic studies, many generic features are extracted in the hope that some would be associated with the biology and thus be predictive [7]. These features strongly depend on several choices regarding the acquisition parameters, preprocessing choices, the segmentation of the volume-of-interest and others, and therefore contain both necessarily correlated and irrelevant features [8, 9]. Radiomics then proceeds by using a feature selection method to identify relevant features and a machine learning model for prediction. It seems natural to consider relevant features of the best-performing model as surrogates for biomarkers because such features contribute to the model’s predictive performance. They should therefore be considered informative and are at least good candidates for biomarkers [10–14].

Unfortunately, from a statistical standpoint, there is often not a single best-performing model. Different choices of feature selection methods and classifiers can lead to models performing only slightly worse than and statistically similarly to the best-performing model. In these cases, the null hypothesis that they are equal cannot be rejected. If the best-performing model’s features are associated with the underlying biology, it raises the question of whether the same features can be considered relevant in statistically similar models.

Therefore, we analyzed on 14 publicly available radiomic datasets whether the selected features of statistically similar models are similar. We employed 8 different feature selection methods and 8 classifiers and measured the predictive performance of the models by area under the receiver operating characteristic curve (AUC-ROC). We compared the stability of the selected features, the similarity, and the correlation among the best-performing models.

## Methods

To ensure reproducibility, only publicly available datasets were employed for this study. Ethical approval for this study was therefore waived by the local ethics committee (Ethik-Kommission, Medizinische Fakultät der Universität Duisburg-Essen, Germany). All methods and procedures were performed following the relevant guidelines and regulations.

### Datasets

We identified publicly available datasets by reviewing open-access journals. A total of 14 radiomic datasets were included in this study (Table 1). As is common for radiomic datasets, these datasets were all high-dimensional; in other words, they contained more features than samples, except for the dataset Carvalho2018. Since the focus of this study is on radiomic features, only features coming from imaging data were used, and other features, e.g., clinical or genetic features, were removed. All available data were merged to reduce the effects of non-identically distributed data.

**Table 1:** Overview of the datasets used for the study

Dataset	*N*	*d*	Dimensionality (#Samples/#Features)	Outcome balance [%]	Modality	Tumor type	Software for feature extraction	Feature selection and classifier	DOI
Arita2018 [32]	168	685	0.25	66	MRI	Brain	Inhouse	LASSO and LASSO	https://doi.org/10.1038/s41598-018-30273-4
Carvalho2018 [33]	262	118	2.22	59	FDG-PET	NSCLC	Inhouse	LASSO and Cox regression	https://doi.org/10.1371/journal.pone.0192859
Hosny2018A (HarvardRT) [34]	293	1005	0.29	54	CT	NSCLC	Pyradiomics	mRMR and random forest	https://doi.org/10.1371/journal.pmed.1002711
Hosny2018B (Maastro) [34]	211	1005	0.21	28	CT	NSCLC	Pyradiomics	mRMR and random forest	https://doi.org/10.1371/journal.pmed.1002711
Hosny2018C (Moffitt) [34]	183	1005	0.18	73	CT	NSCLC	Pyradiomics	mRMR and random forest	https://doi.org/10.1371/journal.pmed.1002711
Ramella2018 [35]	91	243	0.37	55	CT	NSCLC	Inhouse	Random forest for both	https://doi.org/10.1371/journal.pone.0207455
Lu2019 [36]	213	658	0.32	43	CT	Ovarian cancer	Inhouse	Univariate and LASSO + Cox	https://doi.org/10.1038/s41467-019-08718-9
Sasaki2019 [37]	138	588	0.23	49	MRI	Brain	Inhouse	Super PCA and LASSO	https://doi.org/10.1038/s41598-019-50849-y
Toivonen2019 [38]	100	7106	0.01	80	MRI	Prostate cancer	Inhouse	Logistic regression for both	https://doi.org/10.1371/journal.pone.0217702
Keek2020 [39]	273	1323	0.21	40	CT	HNSCC	Inhouse	Univariate Concordance Index and Cox regression as well as random survival forest	https://doi.org/10.1371/journal.pone.0232639
Li2020 [40]	51	397	0.13	63	MRI	Glioma	Artificial Intelligence Kit, GE Healthcare	LASSO + Mann–Whitney-U + correlation and logistic regression	https://doi.org/10.1371/journal.pone.0227703
Park2020 [41]	768	941	0.82	24	US	Thyroid cancer	Inhouse	LASSO for both	https://doi.org/10.1371/journal.pone.0227315
Song2020 [42]	260	265	0.98	49	MRI	Prostate cancer	Pyradiomics	ANOVA, RFE, relief and 10 classifiers	https://doi.org/10.1371/journal.pone.0237587
Veeraraghavan2020 [43]	150	201	0.75	31	DCE-MRI	Breast	Inhouse	No feature selection and random forest	https://doi.org/10.1038/s41598-020-72475-9

For reproducibility reasons only publicly, available datasets were used. The sample size is denoted by *N*, the number of features as *d*, which corresponds to the dimension of the data. Outcome balance denotes the percentage of events in the outcome used. The software that was used to extract the features, the feature selection and classifier methods is reported as stated in the corresponding study. Finally, DOI denotes the identifier of the publication corresponding to the dataset

### Features

All datasets were available in preprocessed form; that is, they contained already extracted features. Extraction and acquisition parameters differed for each dataset. Because in-house software was used, compliance with the Image Biomarker Standardisation Initiative (IBSI) was not always ensured [15]. Texture and histogram features were available for all datasets but shape features were only available for some. Further information on the feature extraction and preprocessing methods used for each dataset can be found in the corresponding study.

### Preprocessing

Additional simple preprocessing was performed to harmonize the data; specifically, missing values were imputed using column-wise mean, and the datasets were normalized using *z*-scores.

### Feature selection methods

Eight often used feature selection methods were employed (Table 2), including LASSO, MRMRe, and MIM. These feature selection algorithms do not directly identify the most relevant features but instead calculate a score for each. Thus, a choice had to be made as to how many of the highest-scoring features should be used for the subsequent classifier. The number of selected features was chosen among *N* = 1, 2, 4, …, 64.

**Table 2:** Overview of all feature selection methods used

Feature selection	Type	Hyperparameters
ANOVA	Filtering	–
Bhattacharyya distance	Filtering	–
ExtraTrees	Wrapper	–
Fast correlation-based filtering (FCBF)	Filtering	–
Kendall correlation	Filtering	–
LASSO	Wrapper	Regularization parameter, fixed at *C* = 1.0
Mutual information (MIM)	Filtering	–
Miinimum redundancy maximum relevance ensemble (MRMRe)	Filtering	Number of ensembles, fixed at 5

Filtering methods assign a score to each feature directly, while wrapper methods use a classifier

### Classifiers

After the choice of the feature selection methods, the choice of classifier is most important because suboptimal classifiers yield inferior results. Eight often-used classifiers were selected (Table 3).

**Table 3:** Overview of all classifiers used during training

Classifier	Hyperparameters
Linear discriminant analysis (LDA)	–
Linear SVM	Regularization parameter *C* in 2**{− 6, − 4, − 2, 0, 2, 4, 6}
Logistic regression	Regularization parameter, *C* in 2**{− 6, − 4, − 2, 0, 2, 4, 6}
Naive Bayes	–
Neural network (three layers)	Neurons in layer 1, 2, 3 in {4, 16, 64}
Random forest	Number of trees in 50, 250, 500
Radial basis function-SVM (RBF-SVM)	Regularization parameter, *C* in 2**{− 6, − 4, − 2, 0, 2, 4, 6}, Kernel parameter *γ* = auto
XGBoost	Learning rate in 0.001, 0.1, 0.3, 0.9, number of estimators in 50, 250, 500

### Training

Training proceeded following the standard radiomics workflow; combinations of feature selection methods and classifiers were used. In the absence of explicit validation and test sets, tenfold stratified cross-validation was employed.

### Evaluation

Predictive performance is the most important metric in radiomics. Therefore, AUC-ROCs were employed for evaluation. Model predictions over all 10 test folds of the cross-validation were pooled into a single receiver operating characteristic (ROC) curve. A DeLong test was used to determine whether models were statistically different.

For the evaluation, we focused on the best-performing model for each dataset as well as the models that were not be shown to be statistically different. A histogram of Pearson correlations between all features was plotted to visually depict the correlations present in the datasets.

### Stability of the feature selection methods

A critical property for the interpretation of features is the stability of the feature selection, meaning whether a given feature selection method will select similar features if presented with data from the same distribution. Using the data from the tenfold cross-validation, we calculated the stability using the Pearson correlation method. Because features are selected prior to the training of the classifier, the stability does not depend on the classifier; rather, it depends on the number of chosen features. The stability might be different for two models that use the same feature selection method but a different number of features. The stability will be 1.0 if, over each cross-validation fold, the very same features are selected.

### Similarity among the feature selection methods

The similarity of the selected features was computed using the Pearson correlation method to determine the discrepancy between the features selected by the best model and those selected by a statistically similar model. A correlation of 1.0 would indicate that the two feature selection methods selected the same features over each cross-validation fold.

### Correlation among the selected features

Because the features in radiomic datasets are known to be highly correlated, two feature selection methods could select features that are different but nonetheless highly correlated. In this case, their similarity would be low; however, this is misleading because the different features would contain similar information. Therefore, correlations among the selected features themselves were measured by computing the average highest correlation of each feature to that of all other features. This measure will be 1.0 if a perfectly correlated feature of another method can be found for each selected feature of one method. More details on the measure can be found in Additional file 1.

### Predictive performance

The stability, similarity, and correlation of statistically similar models could be associated with their predictive performance. For example, if a few features had very high correlation with the outcome in a dataset, it is likely that many statistical similar models will be able to identify these features. Therefore, a larger correlation among them could be observed. Similarly, if the dataset contains many relatively uninformative features, it is conceivable that the selection of one feature over another depends on the model. Therefore, in this case, lower correlations among models would be observed. Hence, a linear regression was performed to relate the AUC-ROCs to stability, similarity, and correlation.

### Software

Python 3.6 was used for all experiments. Feature selection methods and classifiers from scikit-learn 0.24.2 [16] and ITMO_FS 0.3.2 [17] were utilized.

### Statistics

Descriptive statistics were reported as means and ranges, computed using Python 3.6. *p* values less than 0.05 were considered significant. No adjustments for multiple testing were performed. AUC-ROCs were compared using a DeLong test using the R library pROC.

## Results

**Fig. 1:**
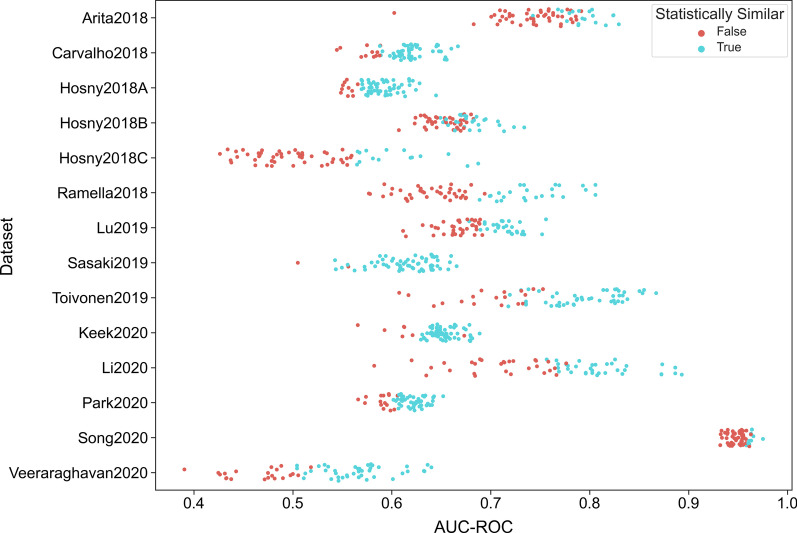
Graphical overview of the predictive performance of all models. The AUC-ROC of all computed models were plotted for all datasets. Those models that cannot be statistically shown to be different from the best model were marked in cyan color, while those that were worse were marked in orange color

Overall, 3640 models for each of the 14 datasets were fitted, each with stratified tenfold cross-validation. For evaluation, the model with highest AUC-ROC was selected for each combination of the 8 features selection methods and 8 classifiers, yielding 64 models. A DeLong test was used to compare these models to the overall best performing model to calculate statistical difference (Fig. 1). For 508 of 882 models, the hypothesis that the AUC-ROCs were different from those of the best performing model could not be rejected (Table 4). This corresponds to 58% of all models (*N* = 508/882) and to roughly 36 of the 64 models per dataset.

**Table 4:** Counts of how many models were statistically not different to the best model for each dataset, sorted by AUC-ROC of the best model

Dataset	AUC-ROC of best model	Number of stat. eq. models
Song2020	0.98	9
Li2020	0.89	34
Toivonen2019	0.87	45
Arita2018	0.83	18
Ramella2018	0.81	23
Lu2019	0.76	27
Hosny2018B	0.73	26
Hosny2018C	0.69	14
Keek2020	0.69	56
Carvalho2018	0.67	52
Sasaki2019	0.67	61
Park2020	0.65	48
Hosny2018A	0.64	53
Veeraraghavan2020	0.64	42

### Datasets

**Fig. 2:**
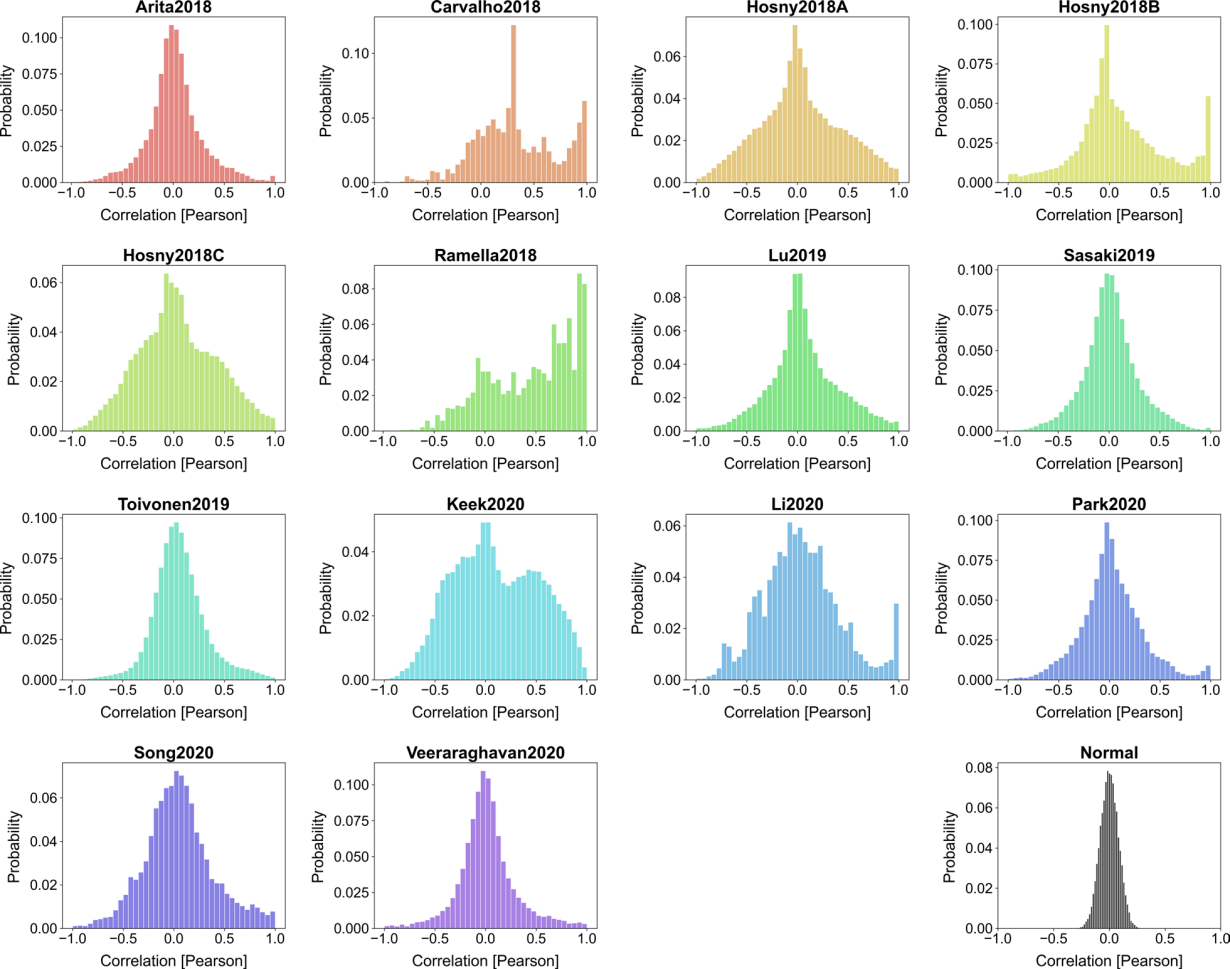
Histogram of the Pearson correlation between all features. The “Normal” histogram was obtained by creating a dummy dataset that only contained independent and normally distributed features and serves as a reference

Plotting the Pearson correlation among all features revealed that some datasets had many highly correlated features (Fig. 2). All datasets deviated largely from the histogram of a dataset with normally distributed and independently chosen columns. Although Toivonen2019 is very close, it still revealed many highly correlated features, as can be seen in the fat right tail.

### Stability of the feature selection method

**Fig. 3:**
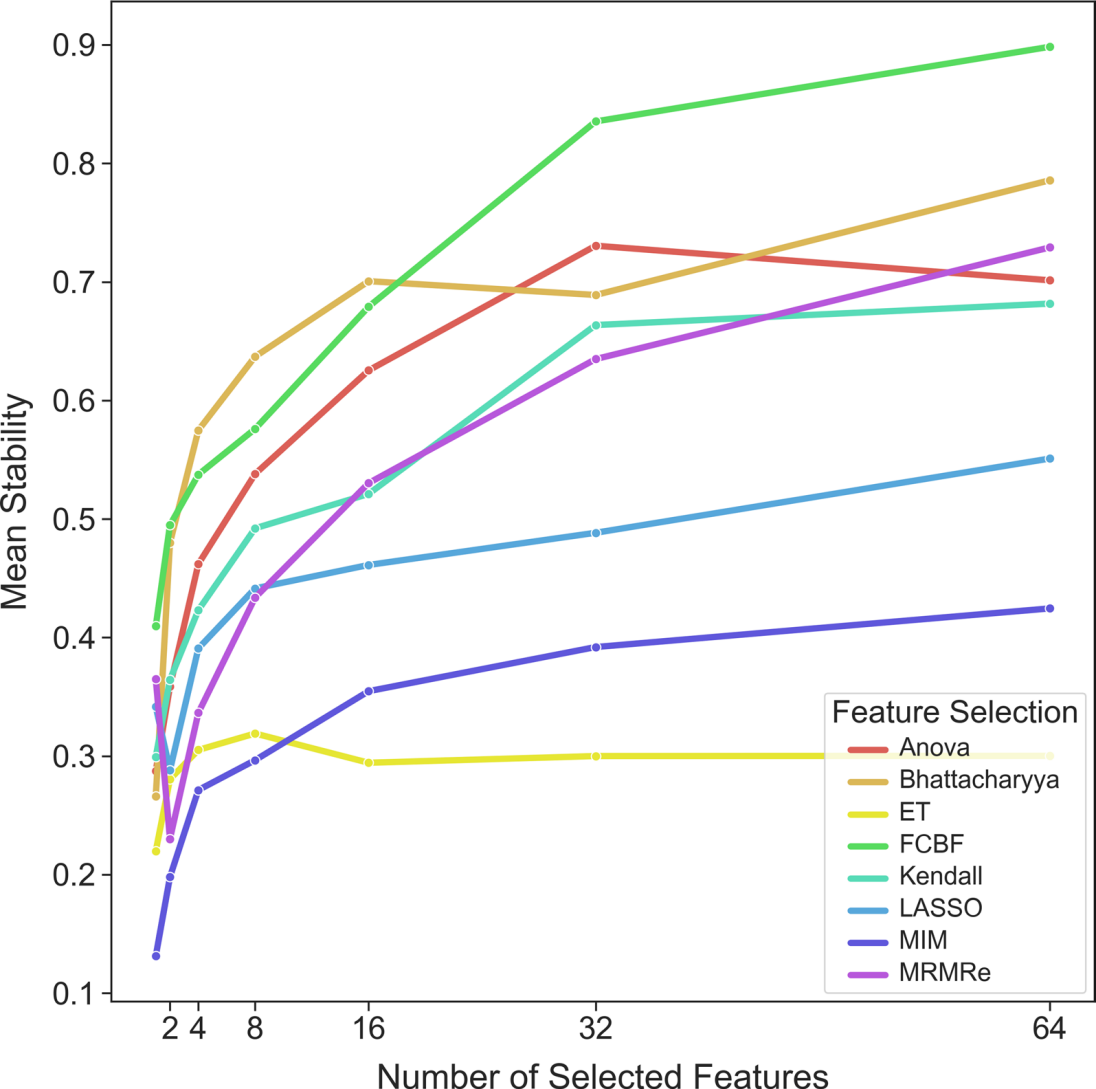
Relation of feature stability with the number of selected features

**Fig. 4:**
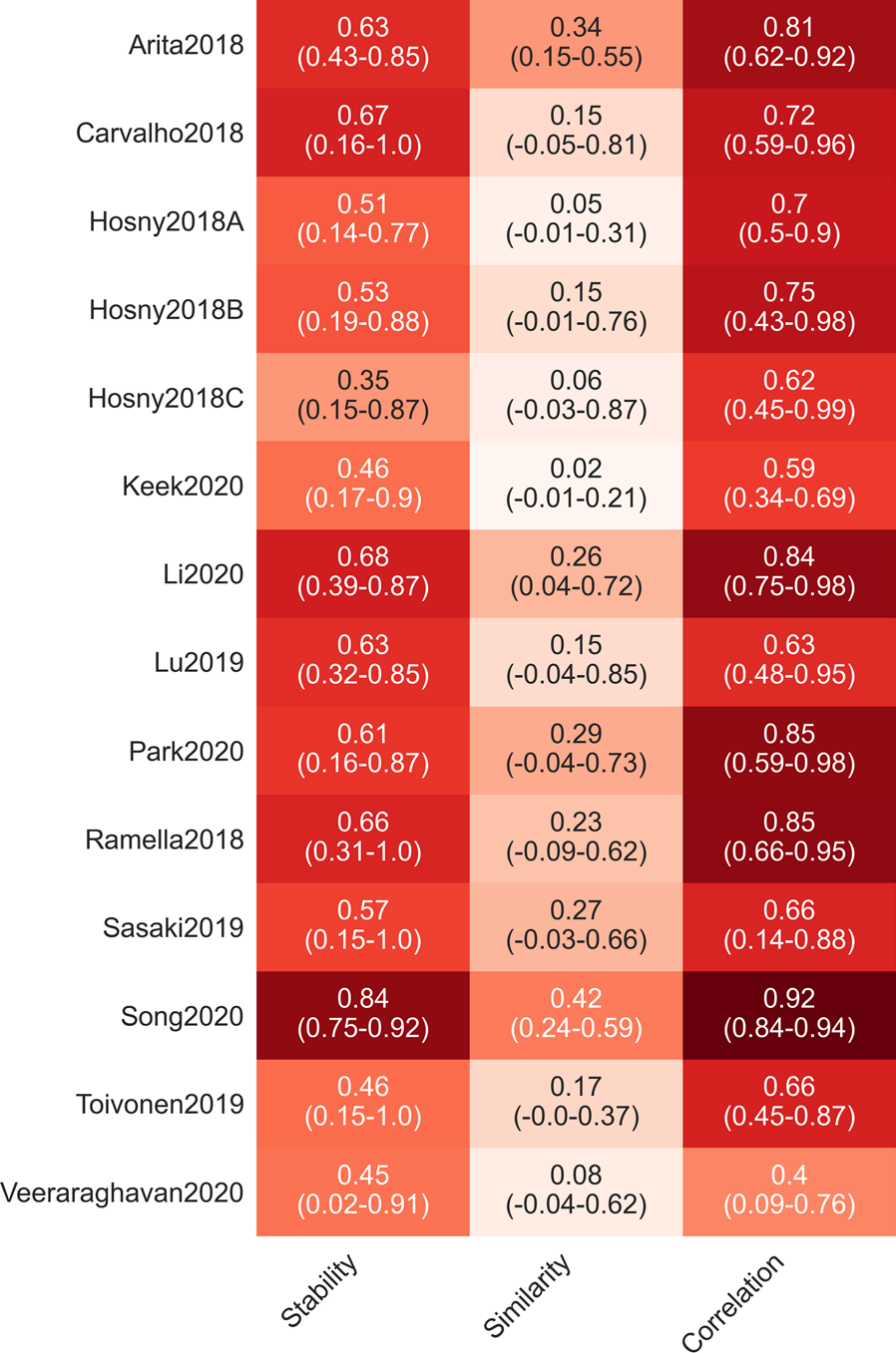
Analyzed measured for all statistically similar models. The range is given in parentheses, the color of each cell corresponds to the stated measure

In general, selecting more features resulted in higher stability (Fig. 3). The three simpler methods (Anova, Bhattacharyya and Kendall) yielded higher stability than the more complex methods (including LASSO and Extra Trees). Overall, the stability of the methods was moderate (Fig. 4). Results for each dataset can be found in Additional file 2 and Additional file 3.

### Similarity among the feature selection methods

The average similarity between the features selected by statistically similar models was rather low (Fig. 4). There were almost always models with selected features that were not similar to those of the best model. In all cases, the average similarity among the feature selection methods was lower than their stability (Fig. 4). Details can be found in Additional file 4.

### Correlation among the selected features

The average correlation among the features was much higher than their similarity. For Song2020, on average, the models had a correlation of 0.92, while for Veeraraghavan2020, this figure was only 0.40. Again, there were often models with features that were only moderately correlated with the features of the best model. Results for each dataset can be found in Additional file 5.

### Predictive performance and feature stability

**Fig. 5:**
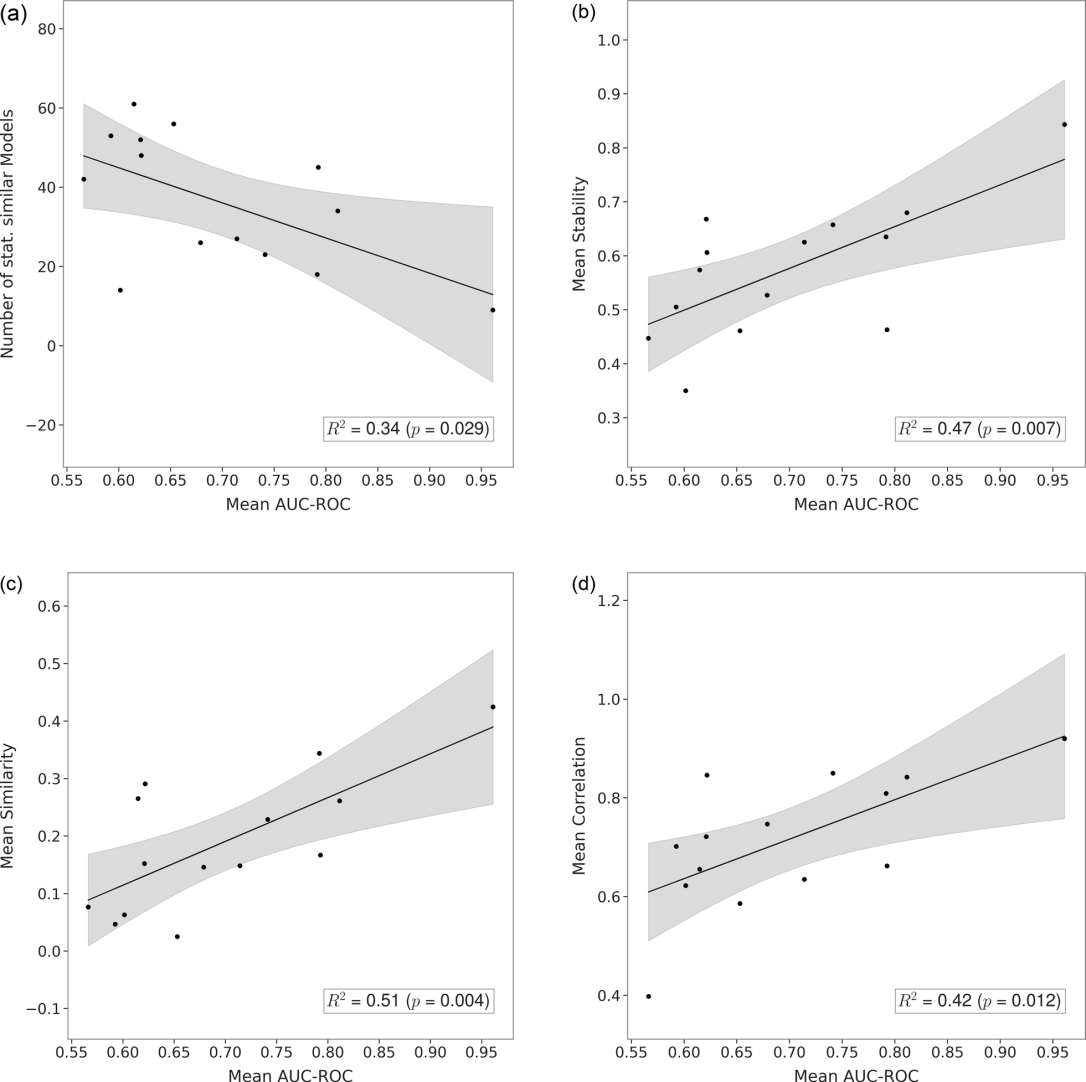
Association of the mean AUC-ROC for all statistically similar models with (**a**) the number of equivalent models, (**b**) stability, (**c**) similarity and (**d**) correlation. Each point represents one dataset

Comparing the mean AUC-ROC to the number of statistically similar models showed a slightly significant and decreasing association (*p* = 0.029; Fig. 5a), that is, the better the best model performed, the less statistically similar models were observed. The associations of AUC-ROCs with stability, similarity, and correlation were higher and, in all cases, positive: a strong positive association (*p* = 0.007; Fig. 5b) was observed for stability, in other words, models that reached higher AUC-ROCs were more stable. An equally strong association between mean AUC-ROC and similarity was found (*p* = 0.004; Fig. 5c), better models seemed to be more similar. On the other hand, for correlation, a weaker association was observed (*p* = 0.012; Fig. 5c).

## Discussion

Radiomics has been intensively studied in pursuit of non-invasive, precise, and personalized medicine, enabling better and more easily deduced diagnoses and prognoses. However, a critical problem of radiomics is that the features are generically defined and strongly depend on acquisition parameters. Therefore, they often lack biological meaning. This reduces reproducibility, that is, the features, and thus the models, often cannot be faithfully recreated if the study is conducted at other sites [18]. Feature selection is used to identify relevant features that could represent the underlying biology and thus be considered biomarkers [19]. In radiomic studies, often only a single feature selection method and classifier combination is considered, without a rationale for why a given method was chosen over others [20–23]. Even if multiple methods are considered, the best-performing model is only compared to the other models in terms of its predictive performance [24–28]. While this approach is justifiable, worse-performing models need not be different from a statistical viewpoint. There is little reason to ignore them.

Therefore, in this study, we considered all models that were statistically similar to the best model and compared their selected features. First and foremost, our study demonstrates that several statistically similar models exist for each dataset. This finding is not surprising, given that the radiomic datasets considered have small sample sizes and that the null hypothesis can only be rejected in the case of a large difference in predictive performance. Nonetheless, approximately 58% of the considered models were statistically similar to the best model, which is much higher than anticipated.

Based on the stability of the feature selection methods, in general, the more features selected, the more stable feature selection is. This is expected because, if all possible features were selected, the correlation coefficient would be equal to 1. Nonetheless, it is surprising that the association is not U-shaped (Fig. 1) because if the datasets contained a small set of relevant features, it could be expected that most feature selection methods would identify them. In this case, the stability would be quite high for a small number of features; however, this was not observed (Fig. 3). There are two possible reasons for this: either the feature selection algorithms were not able to identify those relevant features, or the datasets, in general, did not contain such sets. In the latter case, considering the low stability of the feature selection methods, this could mean that the interpretation of relevant features as biomarkers is doubtful at best.

Consequently, the similarity among the models was also very low. Almost no similarity could be seen for some datasets, and the largest similarity was only moderate (0.42). Therefore, even if the best model was relatively stable in terms of the selected features, thus hinting toward a set of relevant features, in most cases, there is a similarly performant model that would yield completely different features.

Stability and similarity might be misleading because features are highly correlated in radiomic datasets and different features could still express related information. Therefore, in addition to similarity, the correlation of the selected features was compared. Indeed, the correlation was higher than seen for similarity and at least moderate (> 0.40) on average for all datasets.

Taking these observations together, it seems clear that an interpretation of relevant features as biomarkers cannot be obtained *en passant* during modeling with machine learning because these results are not based on causality but rather on correlation. Radiomic datasets are high-dimensional, so results are often abundant and partly random.

Intuitively, a higher overall AUC-ROC should be associated with a higher mean association among statistically similar models, because a higher AUC-ROC could indicate that there exists a set of relevant features that the models can identify. Indeed, regression results indicate that models with higher AUC-ROCs seem to have higher stability and similarity as well as slightly higher correlation. This means that feature relevance in models that do not perform well must be determined cautiously. Indeed, for all datasets, the best model was significantly different from the constant model (predicting a probability of 0.5 for each sample; *p* < 0.05), but for some, the predictive performance was too low (< 0.70) to be clinically useful. However, the results were valid to a large extent for the datasets with higher AUC-ROCs—for example, for Song2020, where the AUC-ROC of 0.98 is high enough for possible clinical use.

When performing feature selection, the expectation is that there is a single set of relevant features and that these can be precisely determined. While this intuition may be correct for low-dimensional datasets, radiomic datasets are high-dimensional and are highly correlated. In theory, both problems could be prevented by acquiring larger samples and more specific features. Regrettably, increasing sample size is problematic, if only because of the need for segmentations, which, currently, are often still delineated manually. Furthermore, performing decorrelation in a high-dimensional setting is unlikely to be useful because correlated features might complement one another [29]. As decorrelation can be regarded as an unsupervised feature selection method, it might not perform any better than a supervised feature selection method. Principal component analysis, on the other hand, could be more suitable; however, due to features being recombined, no kind of association can be made with the biological underpinning.

Although the current radiomics pipeline is generally accepted as state-of-the-art, deep learning methods have been considered. These forgo the tedious task of generating features and feature selection methods [30, 31]. Radiomic models with generic features are often regarded being more interpretable than deep learning models, but our results demonstrate that this presumed superiority is not necessarily the case.

While we obtained our results using cross-validation, other studies have used a train-test split which was then tested on an independent validation set. Using such a training scheme might give the impression that the selected features are relevant and stable, but this is misleading because a simple split neither considers the stability of the model nor the fact that a disjoint set of features could produce a model with statistically similar predictive performance. Nonetheless, having an explicit validation set available would provide a more precise picture because it is conceivable that statistically similar models would produce different results if an external dataset were used.

We focused only on a few often-used feature selection algorithms and classifiers, but we believe that adding more methods to the study would only enhance our results. The same would be true if the hyperparameters of the classifiers were more heavily tuned. We also did not account for multiple testing; doing so would increase *p* values, making more models statistically similar. Thus, our results can be thus considered a lower limit.

## Conclusion

Our study demonstrated that the relevance of features in radiomic models depends on the model used. The features selected by the best-performing model were often different than those of similarly performing models. Thus, it is not always possible to directly determine potential biomarkers using machine learning methods.

## Supplementary Information

**Additional file 1.** Details on the stability, similarity and correlation measures.

**Additional file 2.** Relationship between feature stability and number of features selected for each dataset.

**Additional file 3.** Results for the stability of feature selection methods for each data set.

**Additional file 4.** Results for the similarity of feature selection methods for each data set.

**Additional file 5.** Results for the correlation of the selected features for each data set.

## Data Availability

All datasets generated and/or analyzed during the current study are publicly available (https://github.com/aydindemircioglu/radInt).

## Abbreviations

AUC-ROC: Area-under-curve of the receiver-operating-characteristics
FCBF: Fast correlation-based filtering
IBSI: Image Biomarker Standardisation Initiative
LASSO: Least Absolute Shrinkage and Selection Operator
MIM: Mutual information measure
MRMRe: Minimum redundancy maximum relevance ensemble
ROC: Receiver-operating-characteristics
