# Supplementary material for: Evaluation of the dependence of radiomic features on the machine learning model
Source: Insights Imaging. 2022 Feb 24;13:28. doi: 10.1186/s13244-022-01170-2 (PMC8873309; doi:10.1186/s13244-022-01170-2)
Supplement: Supplementary file 1 — Additional file 1. Details on the stability, similarity and correlation measures. [file 13244_2022_1170_MOESM1_ESM.pdf]

Supplemental 1

Measurements

Stability measure

The stability between two models was computed by comparing pairwise whether the same features were selected. More specific, let the features selected by a model A, i.e., by the corresponding feature selection method, be given as a binary vector f^A. For example, if the first two features were selected, f^A is the vector (1, 1, 0, 0, …). Since a 10-fold cross-validation was applied, on each fold i such a vector is given, denoted by f_i^A. The stability is now given by the average Pearson correlation over all such pairs:

$$Stab\ (A) = \frac{2}{N * (N-1)} \sum_{\substack{i,j \\ j>i}} Pearson\ (f_i^A, f_j^A)$$

with N = 10 and 1 ≤ i < j ≤ N. If the very same features are selected in each fold, the stability will be 1.0, on the other hand, if on each fold a disjoint set of features are selected, the stability will become 0.0.

Similarity measure

Similarity can be simply understood as the stability between to different models. If the binary vector of selected features of model A over fold I is denoted by f_i^A and the binary vector of selected feature of model B over fold j is denoted f_j^B, then the similarity between the models A and B is given by:

$$Sim\ (A, B) = \frac{1}{N * N} \sum_{i,j} Pearson\ (f_i^A, f_j^B)$$

Here, N is the number of cross-validation folds, i.e., in our case N = 10. Notice that the normalization factor has changed, since no correlation is counted twice like in the case of stability. Also, the similarity is influenced by stability: If a model selects different features on each fold then

its similarity of the model to itself Is not 1, since by definition different folds are compared to each other. For example, if the model selects only feature u in fold 1 and only feature v in fold 2, then the Pearson correlation of them is 0—and the similarity, as an average of all pairwise Pearson correlations, must thus be smaller than 1.

Correlation measure

The correlation measure considers whether the selected features are correlated. This is computed by the following method: Suppose we are given two binary vectors of selected features f_i^A and f_j^B corresponding to the features of model A over fold i and model B over fold j. Then, for each selected feature u in f_i^A, we compute its correlations to all selected features v in f_j^B. The correlation of u to f_j^B is given by the maximum Pearson correlation:

$$uCorr\left(u, f_j^B\right) = \max_{v \in f_j^B,} Pearson\ (u, v).$$

The correlation of f_i^A and f_j^B is then given by averaging all correlations of selected features A.

$$uCorr\left(f_i^A, f_j^B\right) = \frac{1}{|f_i^A|} \sum_{u \in f_i^A} uCorr\ (u, f_j^B)$$

In other words, the correlation takes for each feature of A the maximally correlated feature in B and computes their Pearson correlation. The correlations of A and B over the folds i and j is then given by the average Pearson correlation over all selected features of A. For example, a value of 0.5 would mean that either each feature in fold i of A can be correlated to a feature in fold j of B with a correlation of 0.5, or it could mean for half of the features a “perfect match” could be found, i.e., a feature with correlation 1.0.

The correlation of models A and B is then given by averaging over all folds

$$uCorr\ (A, B) = \frac{1}{N * N} \sum_{1 \leq i, j \leq N} uCorr\ (f_i^A, f_j^B)$$

Here, N is the number of cross-validation folds. Note that by definition the correlation is not symmetric. To symmetrize, we simply take the mean over both correlations, i.e.

$$Corr\ (A, B) = \frac{1}{2}\left(uCorr(A, B) + uCorr\ (B, A)\right)$$
