# Supplementary material for: Evaluation of the dependence of radiomic features on the machine learning model
Source: Insights Imaging. 2022 Feb 24;13:28. doi: 10.1186/s13244-022-01170-2 (PMC8873309; doi:10.1186/s13244-022-01170-2)
Supplement: Supplementary file 2 — Additional file 2. Relationship between feature stability and number of features selected for each dataset. [file 13244_2022_1170_MOESM2_ESM.pdf]

Supplemental 2

Stability vs. number of features

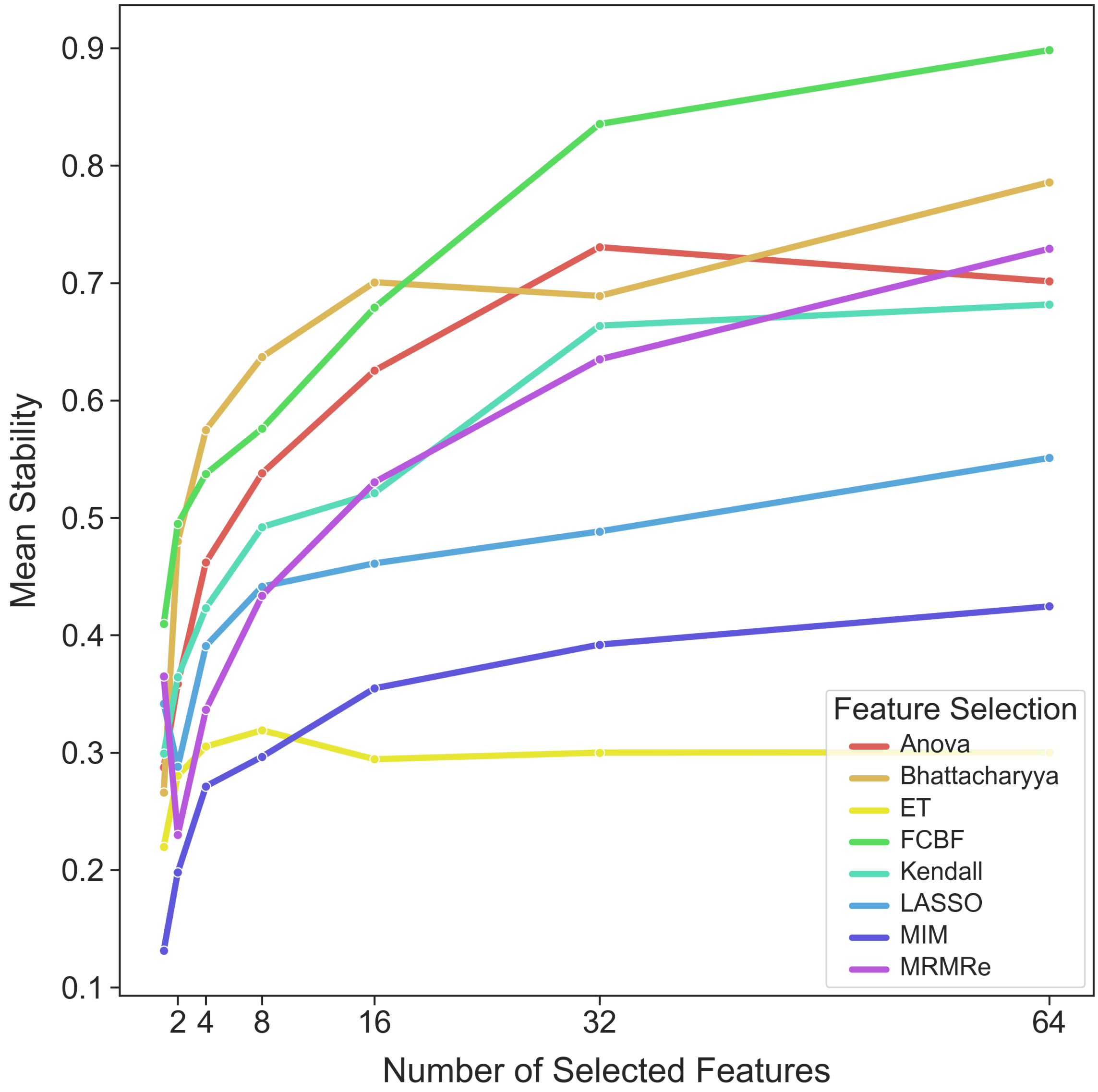

Figure S1: Relation of feature stability with the number of selected features.

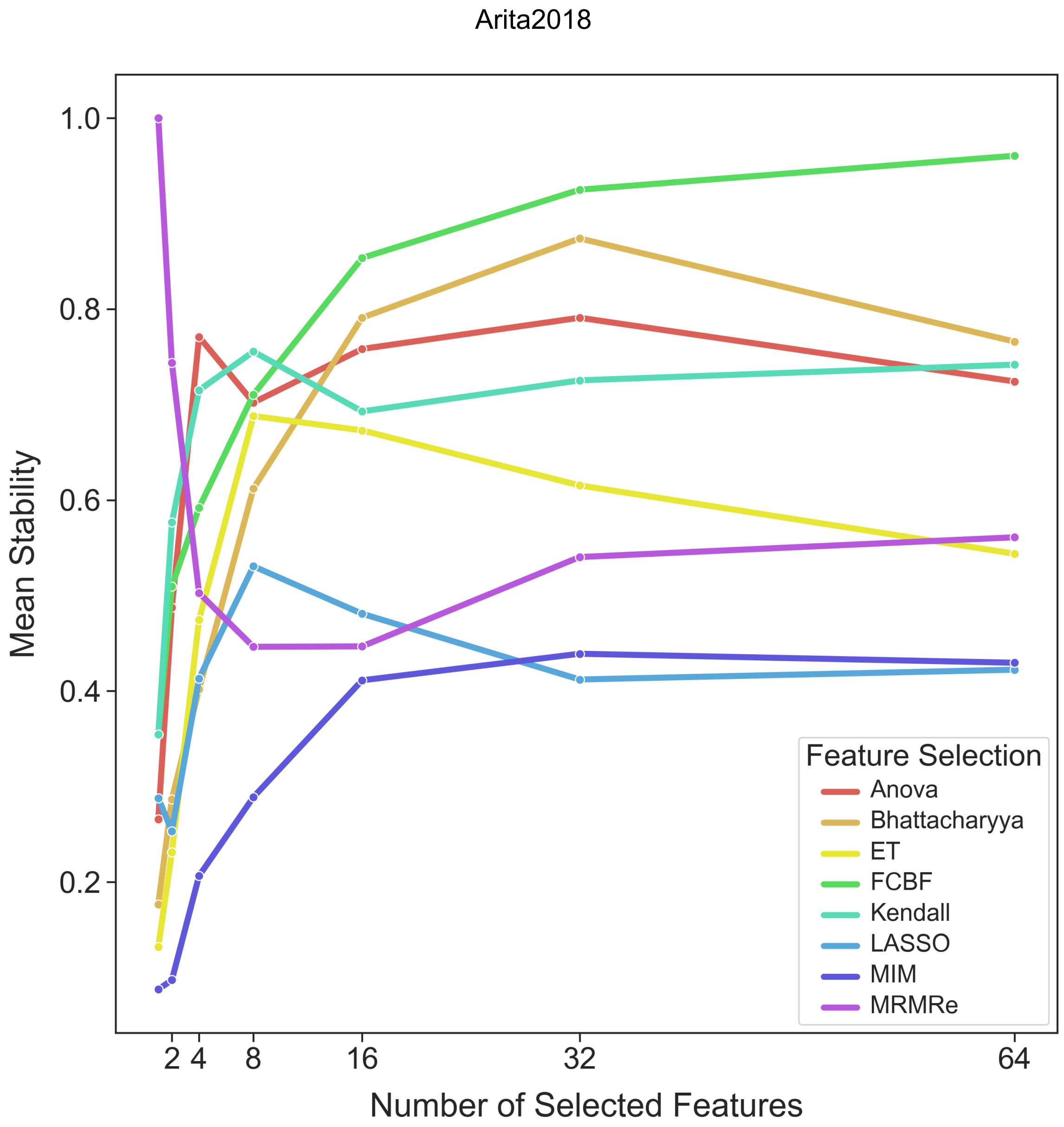

Figure S2: Relation of feature stability with the number of selected features on dataset Arita2018.

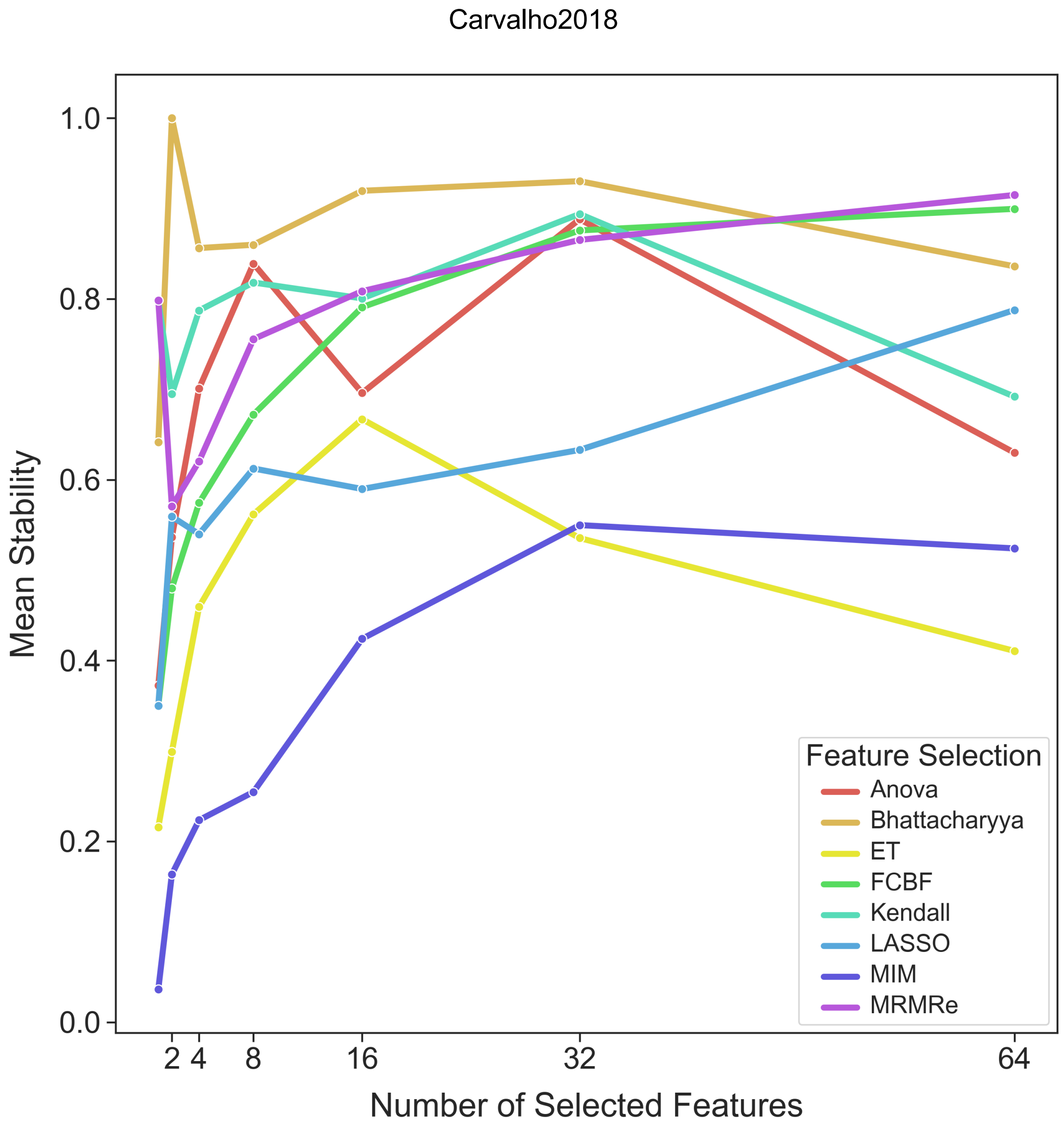

Figure S3: Relation of feature stability with the number of selected features on dataset Carvalho2018.

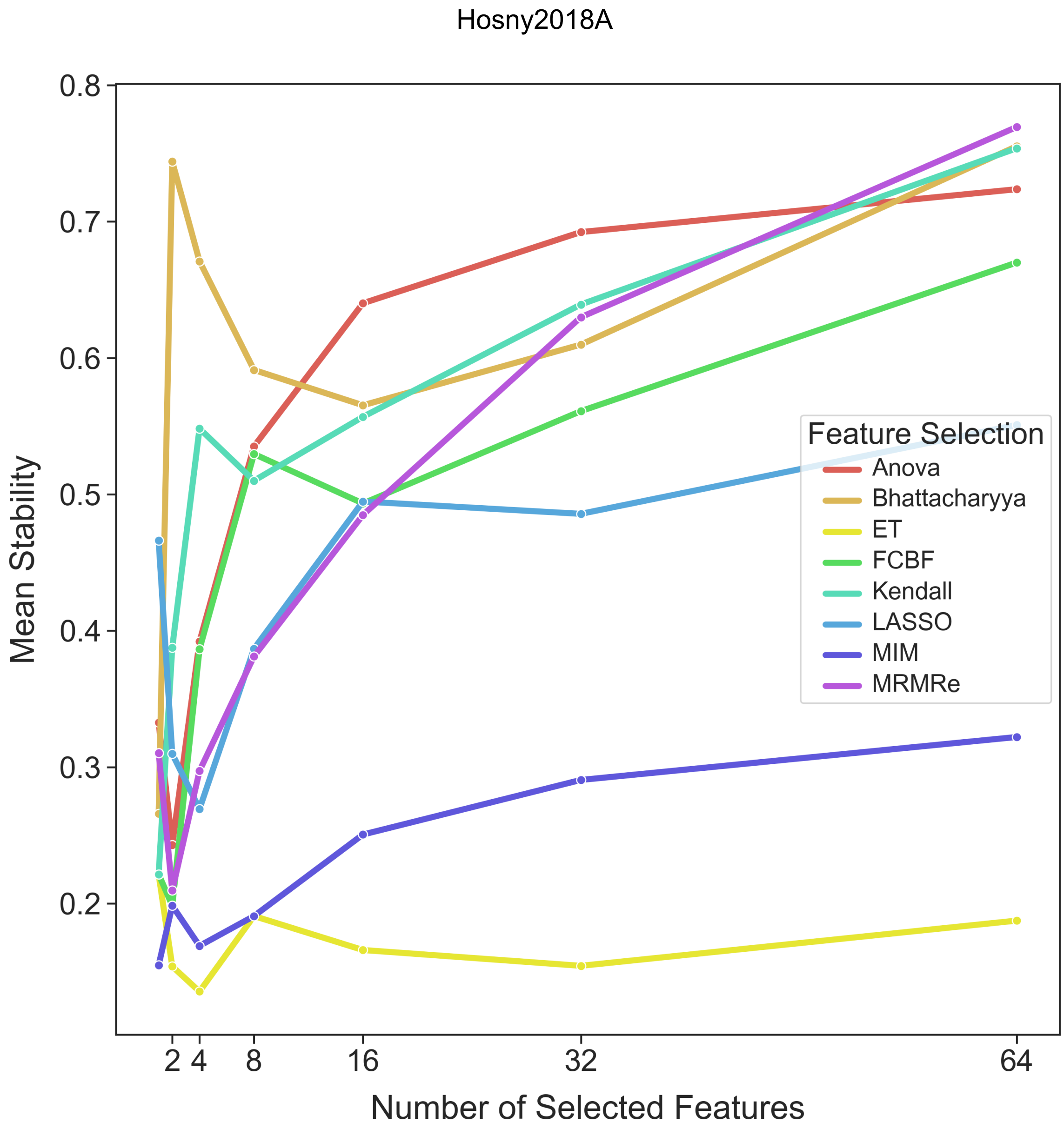

Figure S4: Relation of feature stability with the number of selected features on dataset Hosny2018A.

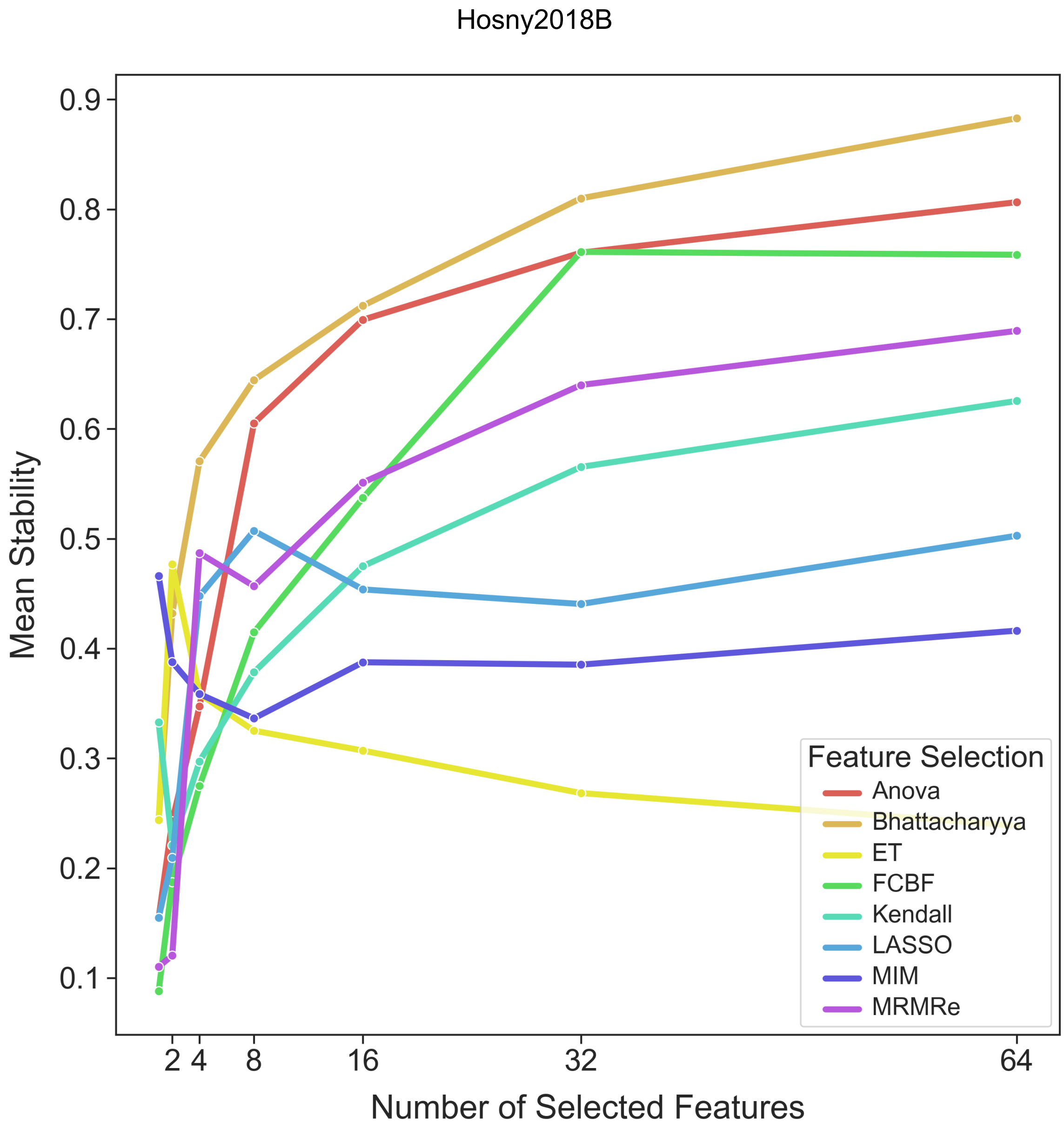

Figure S5: Relation of feature stability with the number of selected features on dataset Hosny2018B.

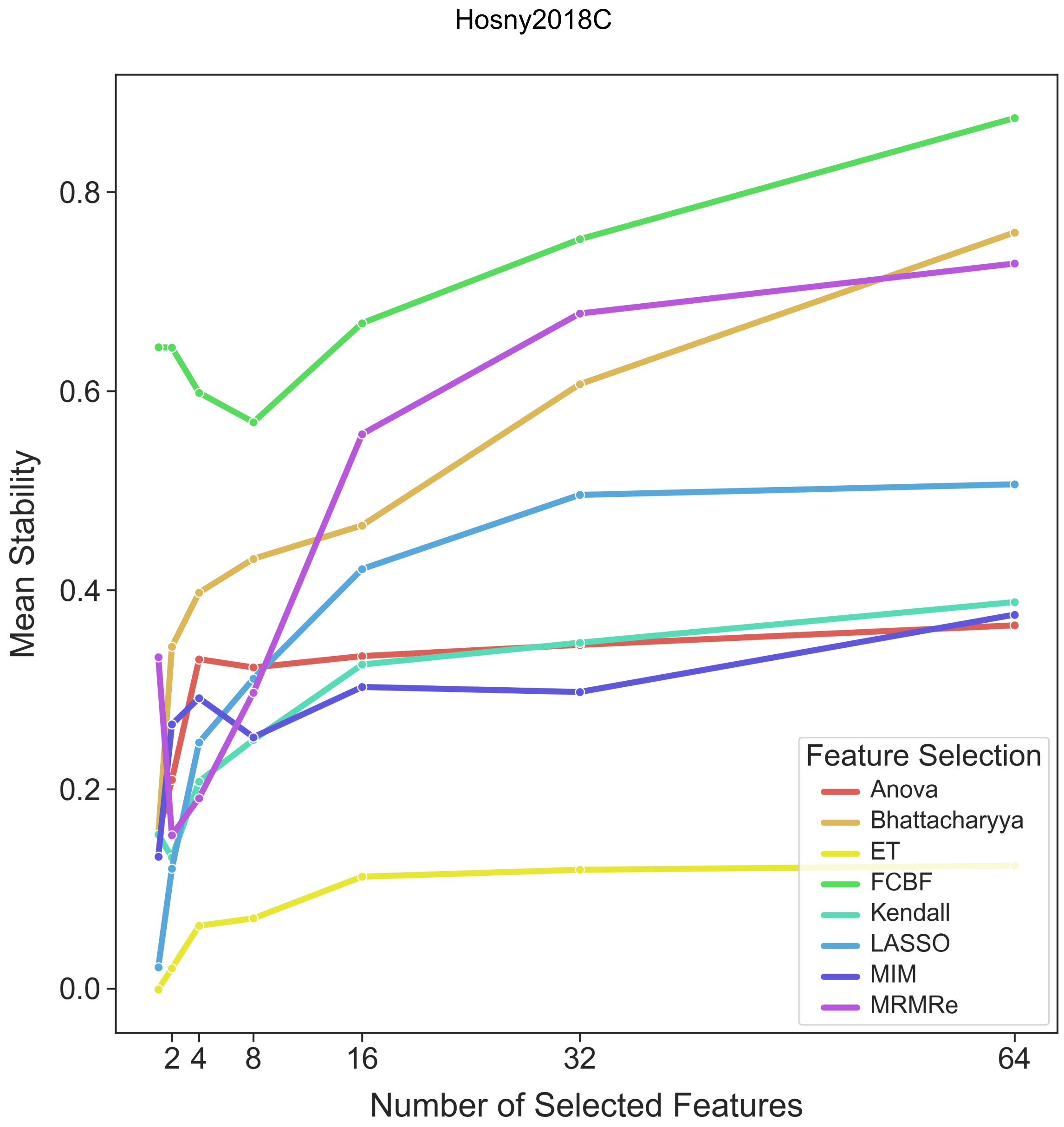

Figure S6: Relation of feature stability with the number of selected features on dataset Hosny2018C.

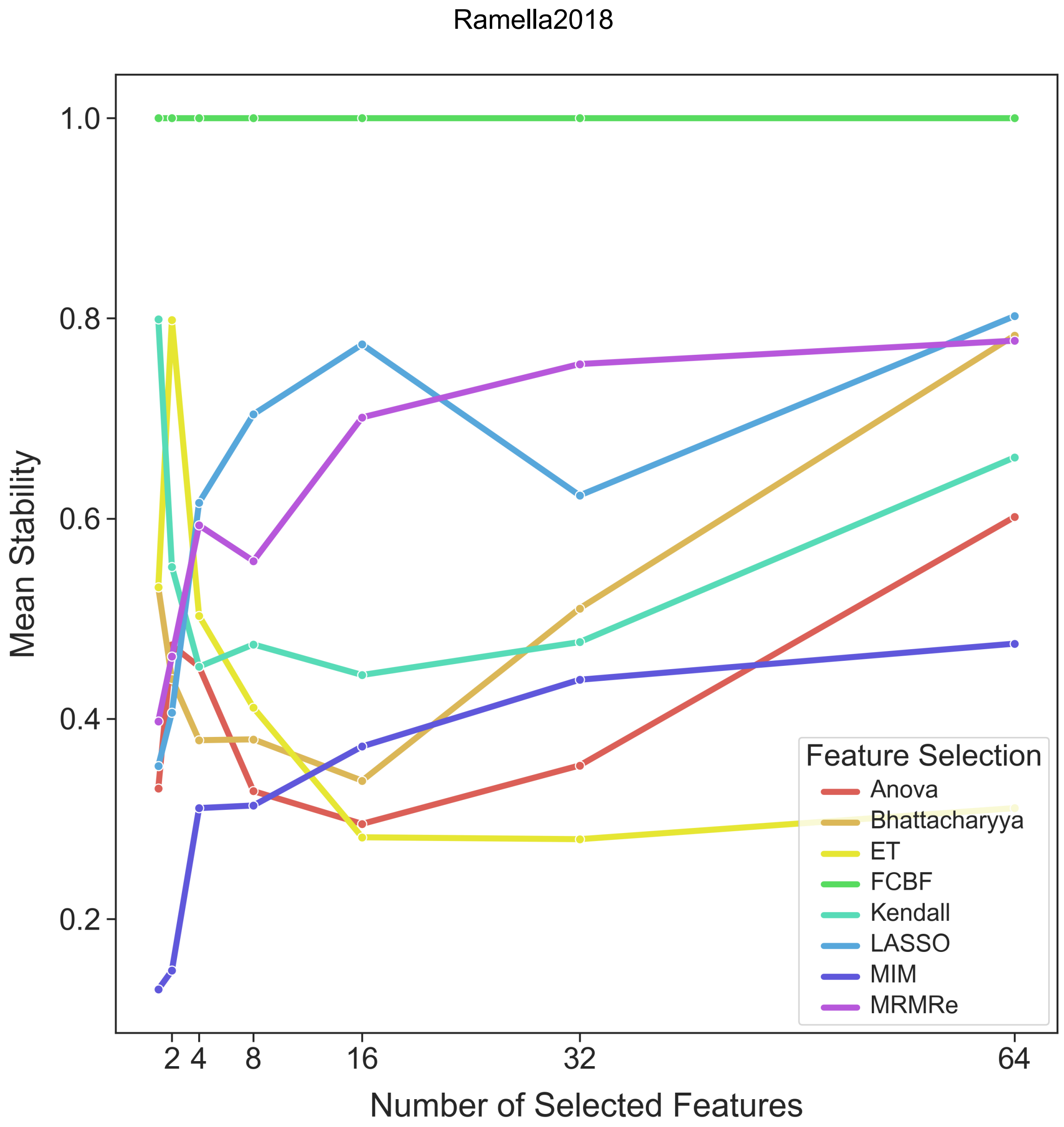

Figure S7: Relation of feature stability with the number of selected features on dataset Ramella2018.

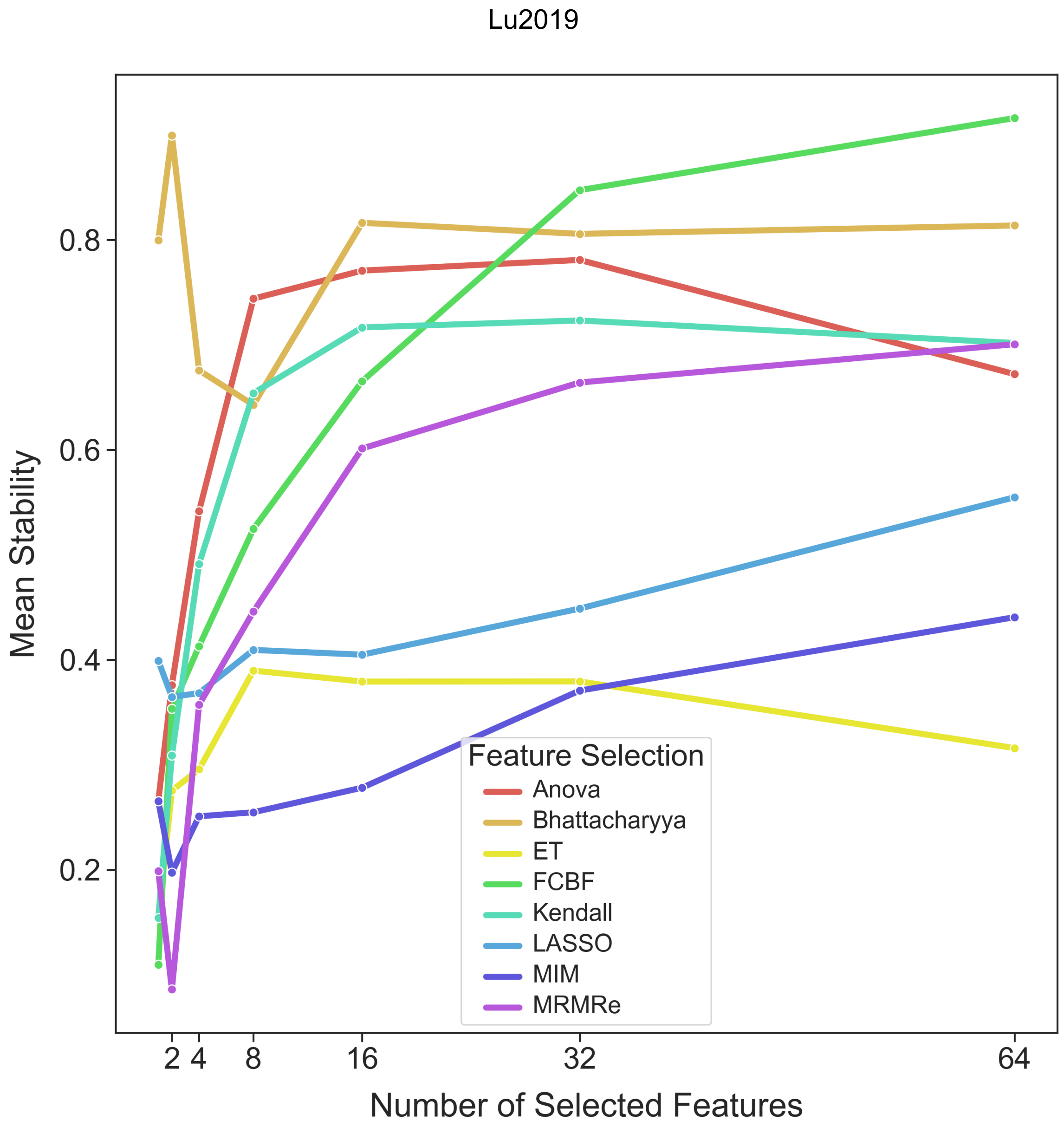

Figure S8: Relation of feature stability with the number of selected features on dataset Lu2019.

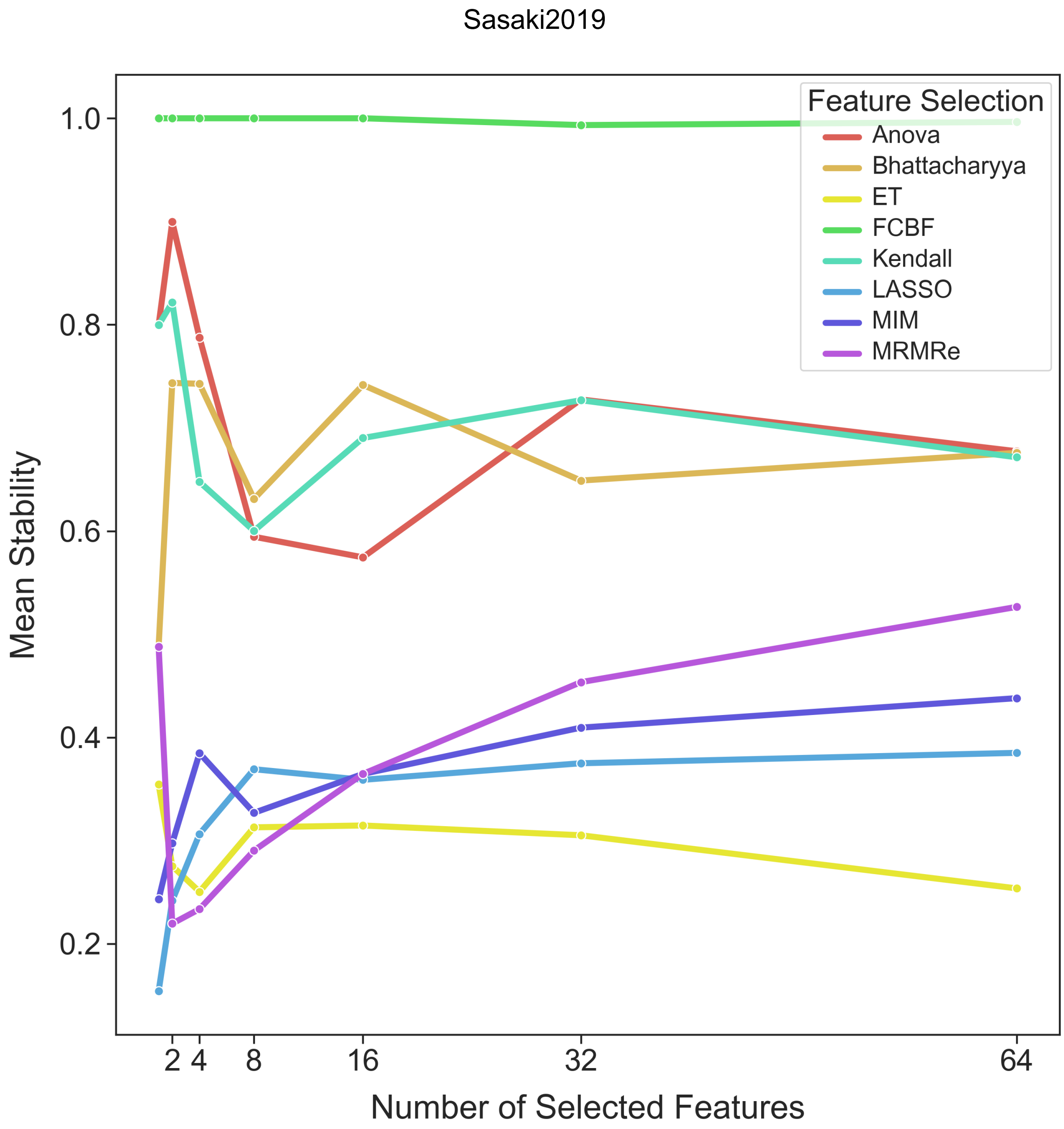

Figure S9: Relation of feature stability with the number of selected features on dataset Sasaki2019.

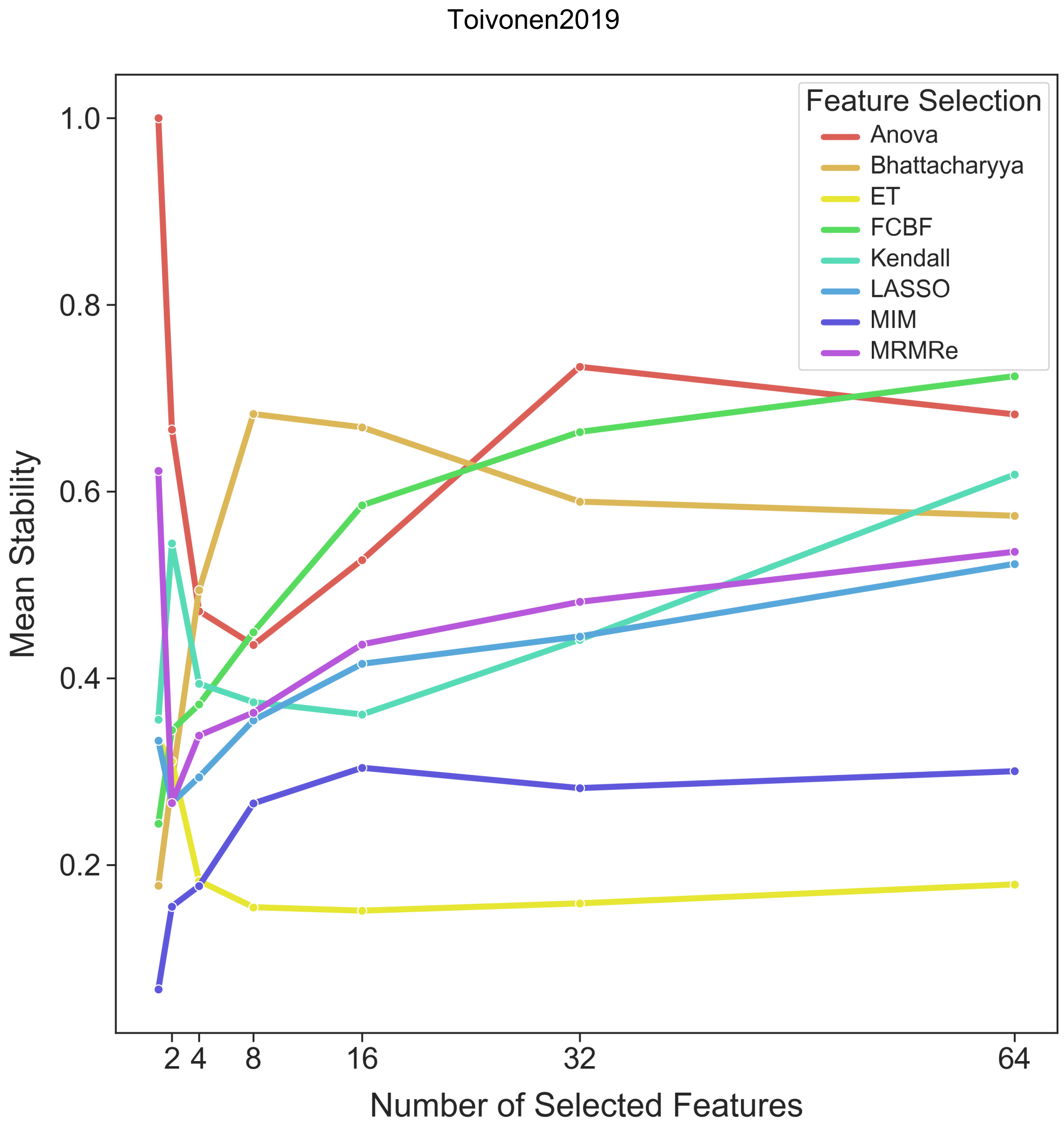

Figure S10: Relation of feature stability with the number of selected features on dataset Toivonen2019.

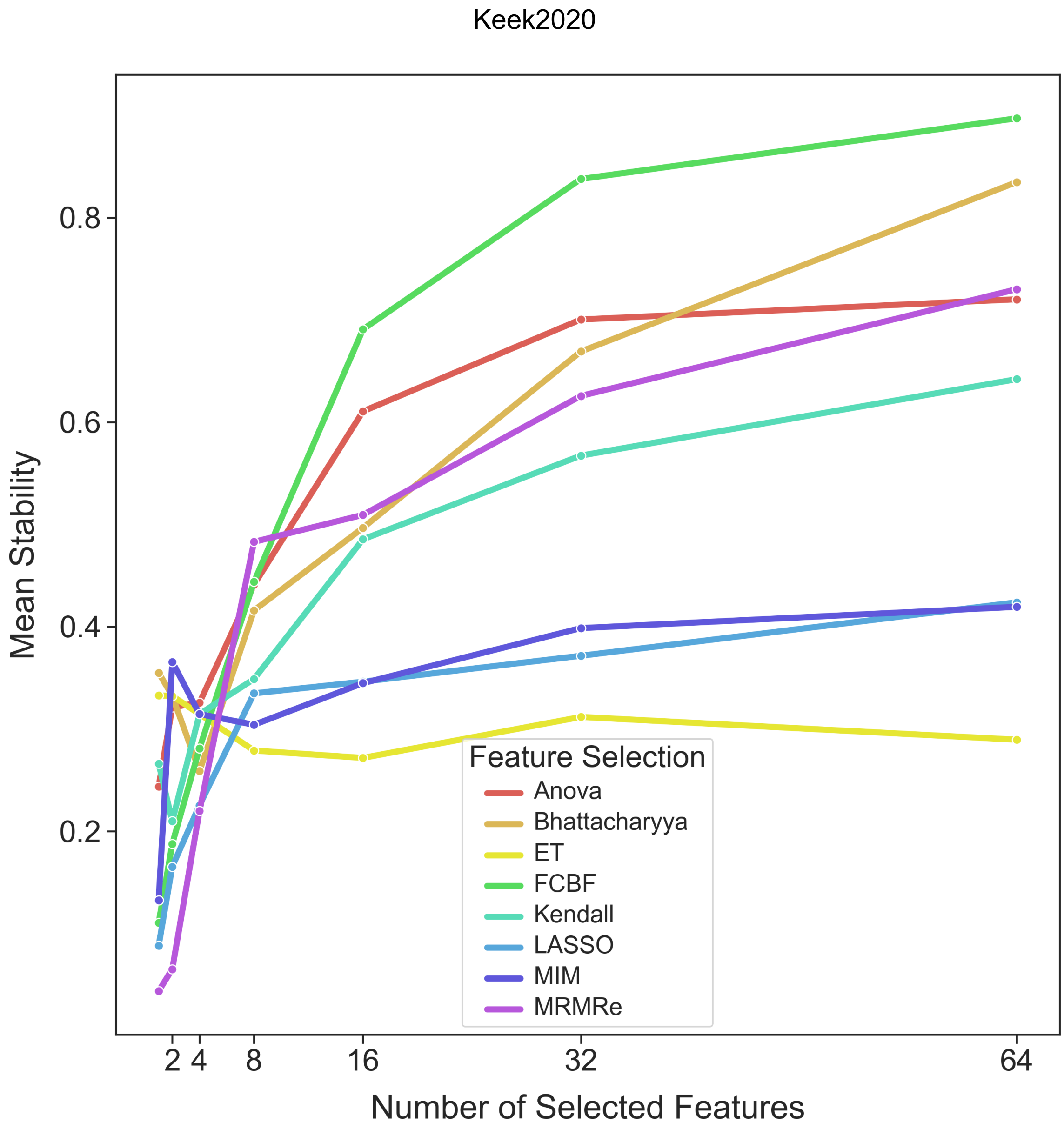

Figure S11: Relation of feature stability with the number of selected features on dataset Keek2020.

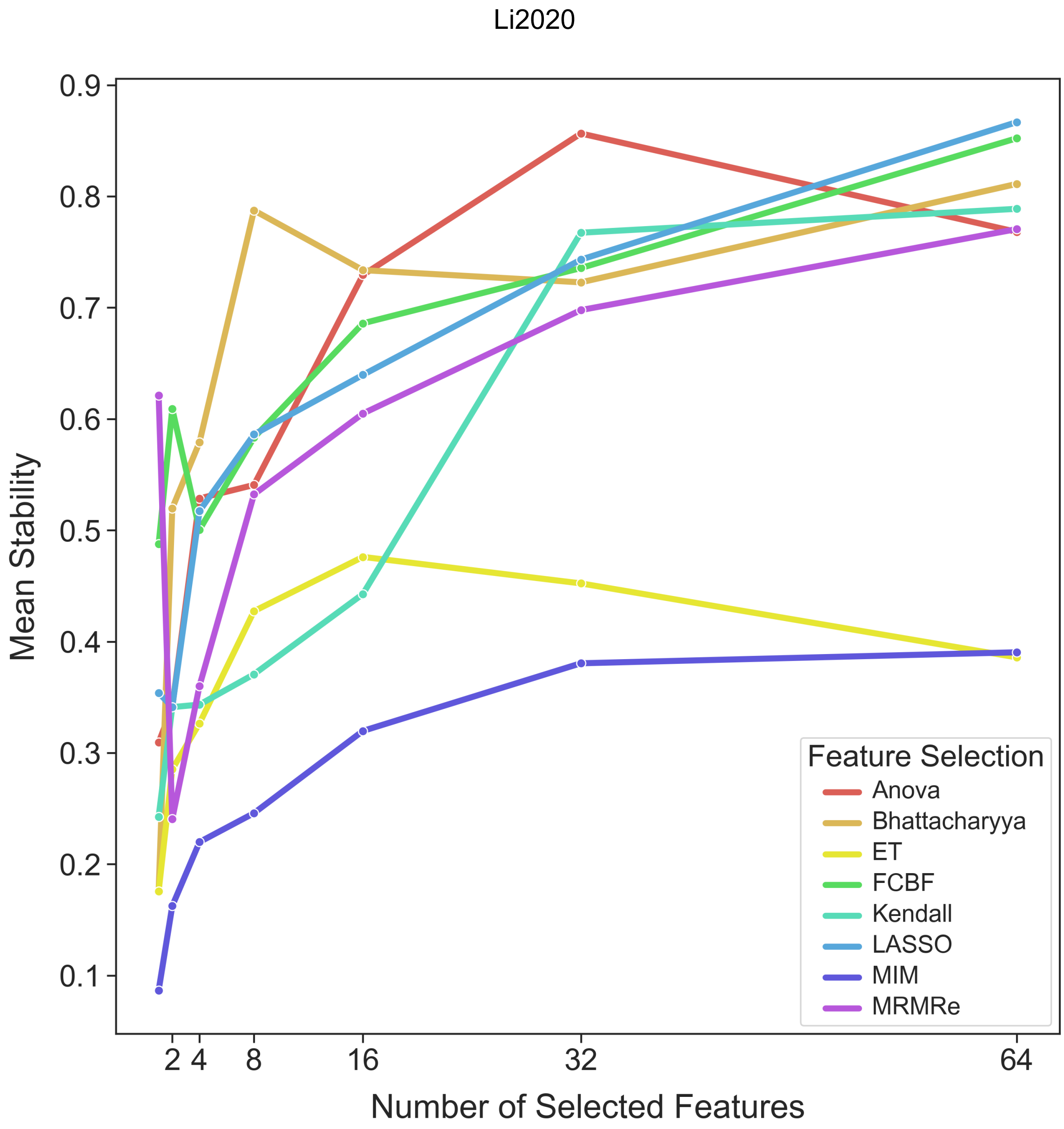

Figure S12: Relation of feature stability with the number of selected features on dataset Li2020.

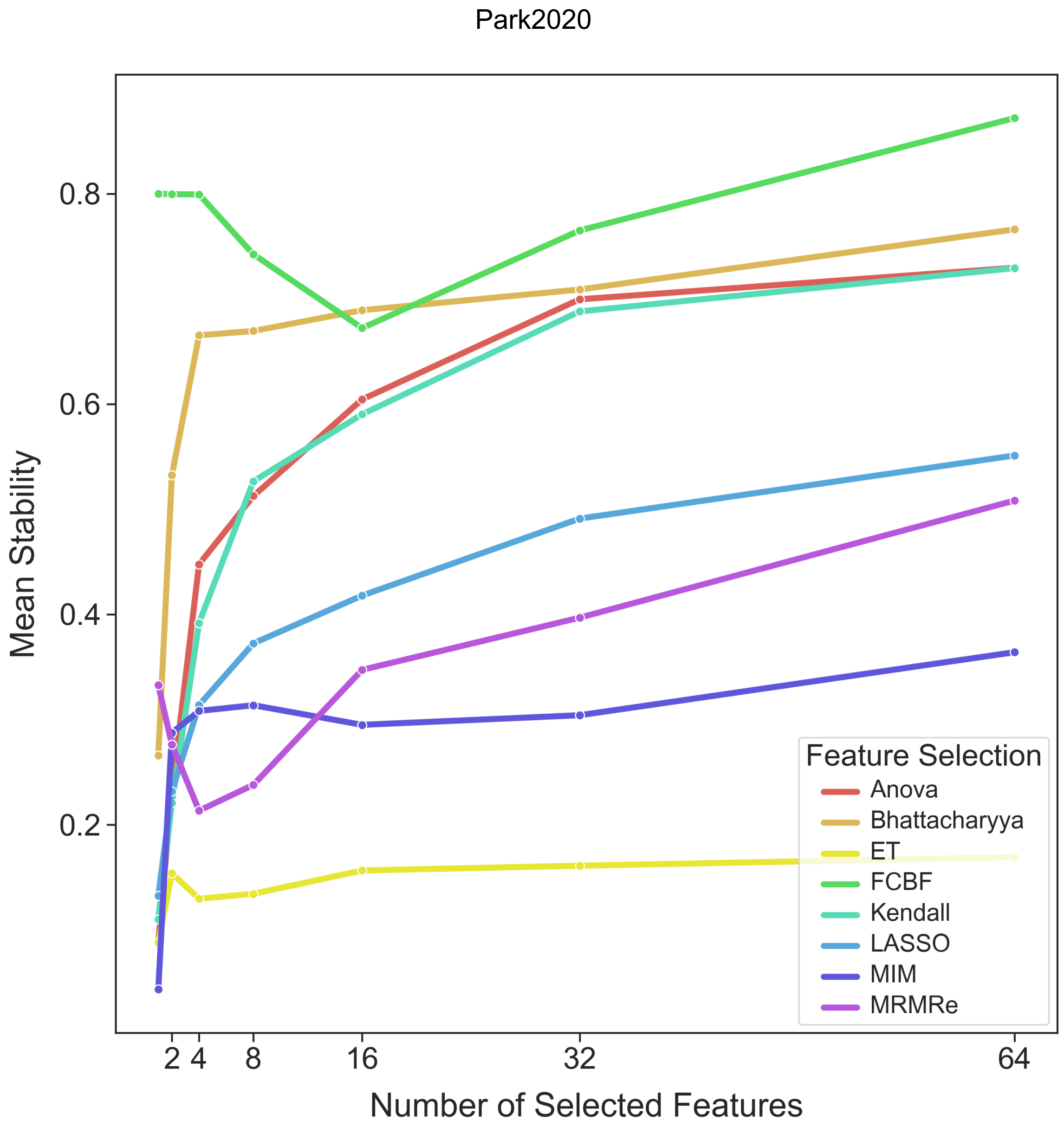

Figure S13: Relation of feature stability with the number of selected features on dataset Park2020.

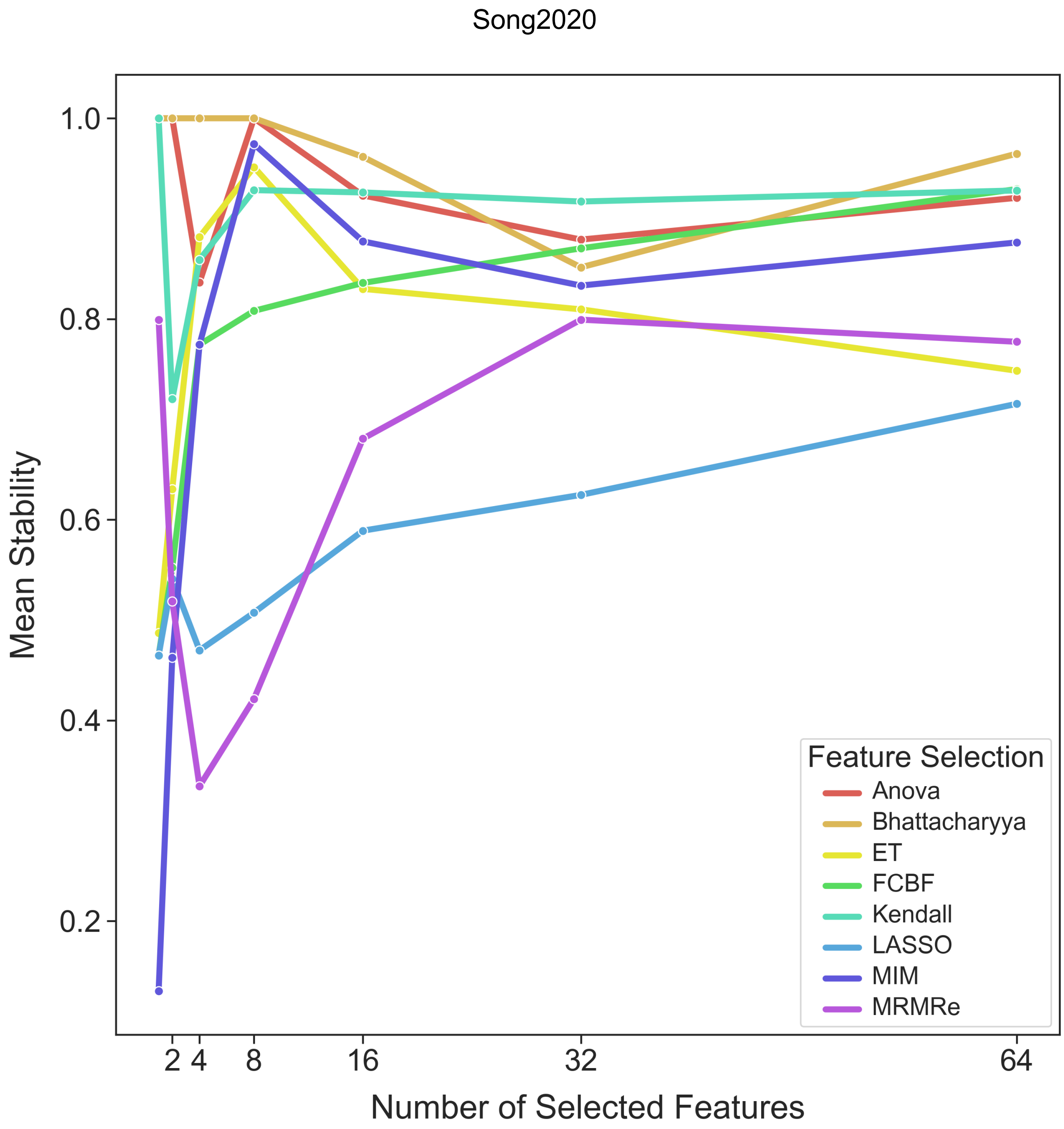

Figure S14: Relation of feature stability with the number of selected features on dataset Song2020.

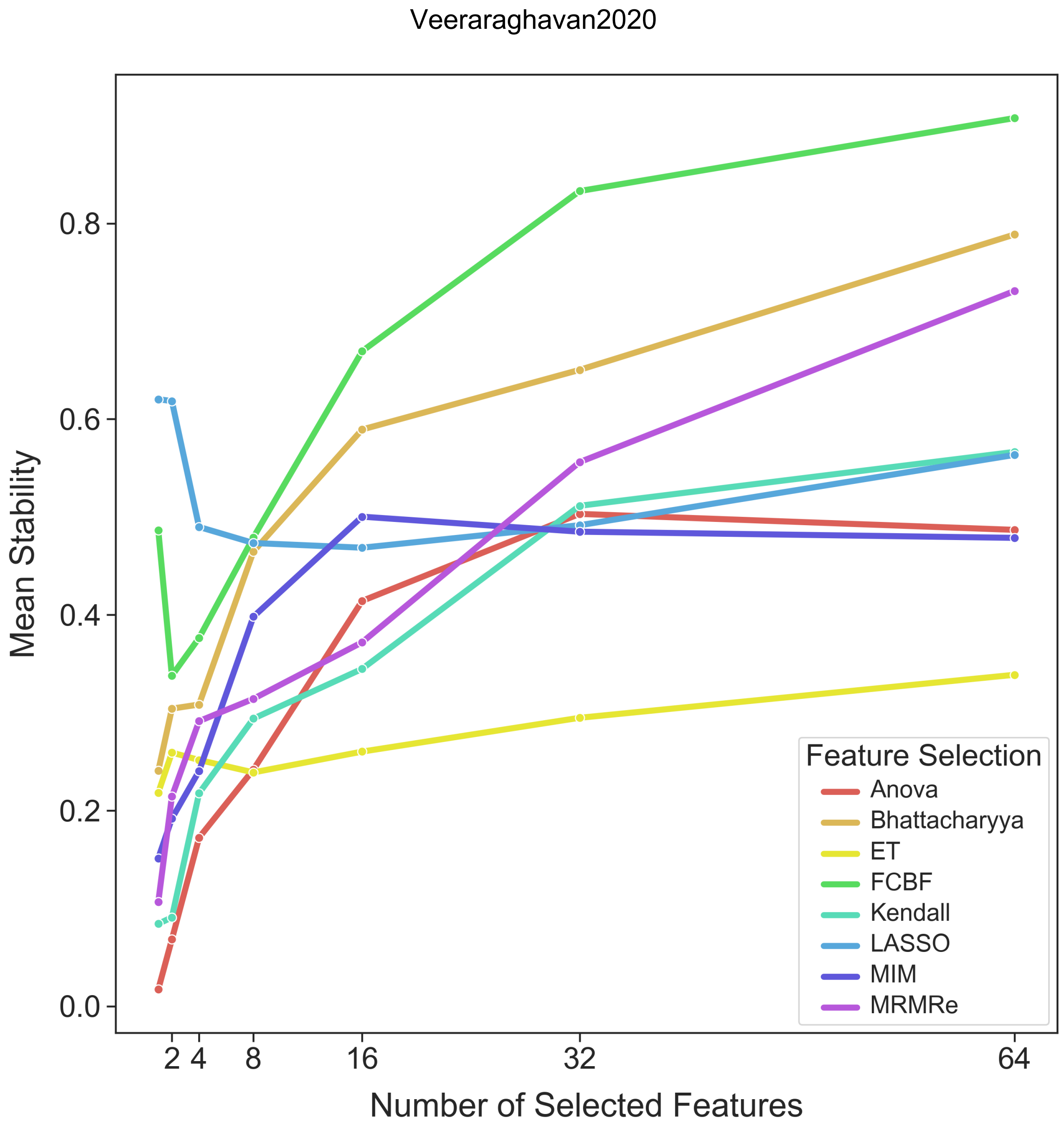

Figure S15: Relation of feature stability with the number of selected features on dataset Veeraraghavan2020.
