# Supplementary material for: Evaluation of the dependence of radiomic features on the machine learning model
Source: Insights Imaging. 2022 Feb 24;13:28. doi: 10.1186/s13244-022-01170-2 (PMC8873309; doi:10.1186/s13244-022-01170-2)
Supplement: Supplementary file 3 — Additional file 3. Results for the stability of feature selection methods for each data set. [file 13244_2022_1170_MOESM3_ESM.pdf]

Supplemental 3

Feature Stability

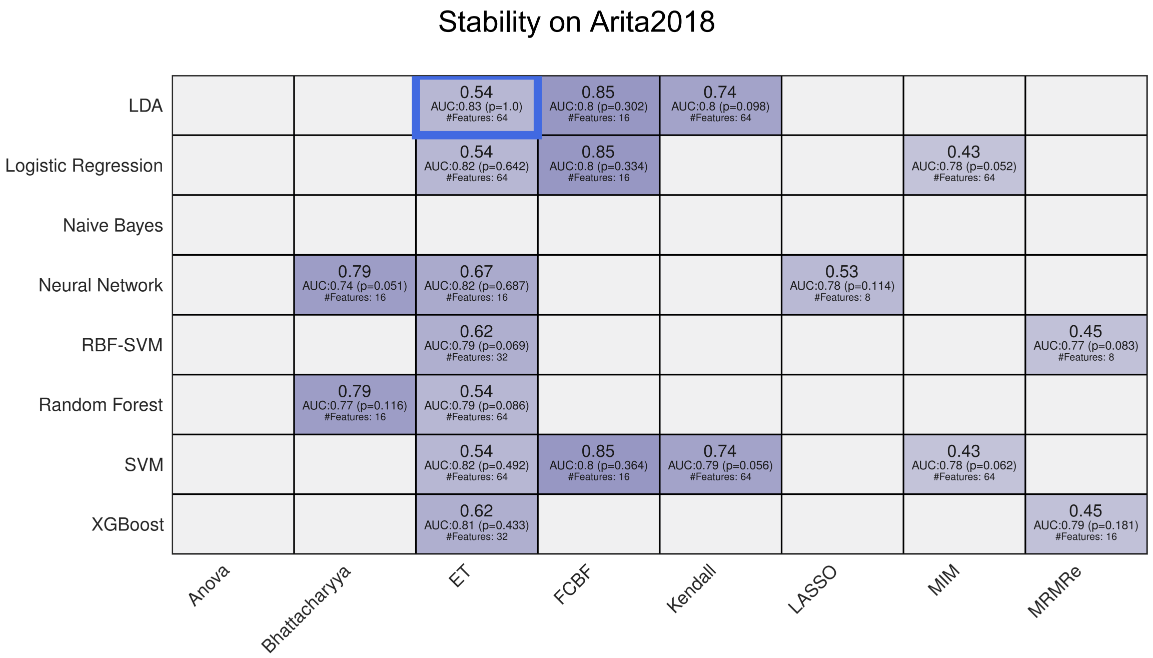

Figure S16: Stability of the models on dataset Arita2018. The best model is framed with a blue border, models that were significantly different to the best model are not shown. Statistical significance was tested using a DeLong test.

Stability on Carvalho2018

	Anova	Bhattacharyya	ET	FCBF	Kendall	LASSO	MIM	MRMRe
LDA	0.7<br>AUC:0.62 (p=0.238)<br>#Features: 4	1.0<br>AUC:0.62 (p=0.223)<br>#Features: 2	0.46<br>AUC:0.61 (p=0.152)<br>#Features: 4		0.79<br>AUC:0.62 (p=0.207)<br>#Features: 4		0.22<br>AUC:0.6 (p=0.144)<br>#Features: 4	0.57<br>AUC:0.61 (p=0.159)<br>#Features: 2
Logistic Regression	0.84<br>AUC:0.62 (p=0.214)<br>#Features: 8	1.0<br>AUC:0.62 (p=0.228)<br>#Features: 2	0.46<br>AUC:0.61 (p=0.144)<br>#Features: 4		0.82<br>AUC:0.62 (p=0.221)<br>#Features: 8	0.59<br>AUC:0.61 (p=0.128)<br>#Features: 16	0.22<br>AUC:0.61 (p=0.166)<br>#Features: 4	0.57<br>AUC:0.62 (p=0.21)<br>#Features: 2
Naive Bayes	0.7<br>AUC:0.62 (p=0.211)<br>#Features: 16	1.0<br>AUC:0.61 (p=0.145)<br>#Features: 2	0.67<br>AUC:0.61 (p=0.131)<br>#Features: 16		0.8<br>AUC:0.62 (p=0.229)<br>#Features: 16			0.57<br>AUC:0.61 (p=0.189)<br>#Features: 2
Neural Network	0.63<br>AUC:0.65 (p=0.547)<br>#Features: 64	0.84<br>AUC:0.64 (p=0.474)<br>#Features: 64	0.56<br>AUC:0.65 (p=0.746)<br>#Features: 8	0.79<br>AUC:0.63 (p=0.339)<br>#Features: 16	0.82<br>AUC:0.65 (p=0.748)<br>#Features: 8	0.59<br>AUC:0.66 (p=0.848)<br>#Features: 16	0.52<br>AUC:0.63 (p=0.303)<br>#Features: 64	0.81<br>AUC:0.67 (p=1.0)<br>#Features: 16
RBF-SVM	0.7<br>AUC:0.65 (p=0.612)<br>#Features: 4	1.0<br>AUC:0.61 (p=0.215)<br>#Features: 2	0.46<br>AUC:0.6 (p=0.116)<br>#Features: 4		0.79<br>AUC:0.65 (p=0.642)<br>#Features: 4	0.59<br>AUC:0.62 (p=0.195)<br>#Features: 16	0.16<br>AUC:0.59 (p=0.093)<br>#Features: 2	0.81<br>AUC:0.66 (p=0.705)<br>#Features: 16
Random Forest	0.84<br>AUC:0.62 (p=0.202)<br>#Features: 8	0.86<br>AUC:0.62 (p=0.218)<br>#Features: 8	0.67<br>AUC:0.6 (p=0.085)<br>#Features: 16		0.82<br>AUC:0.63 (p=0.327)<br>#Features: 8		0.55<br>AUC:0.6 (p=0.055)<br>#Features: 32	0.62<br>AUC:0.65 (p=0.59)<br>#Features: 4
SVM	0.7<br>AUC:0.62 (p=0.208)<br>#Features: 4	1.0<br>AUC:0.62 (p=0.242)<br>#Features: 2	0.46<br>AUC:0.61 (p=0.138)<br>#Features: 4		0.79<br>AUC:0.62 (p=0.204)<br>#Features: 4	0.59<br>AUC:0.61 (p=0.129)<br>#Features: 16	0.22<br>AUC:0.6 (p=0.14)<br>#Features: 4	0.57<br>AUC:0.62 (p=0.218)<br>#Features: 2
XGBoost	0.7<br>AUC:0.62 (p=0.254)<br>#Features: 4	0.86<br>AUC:0.59 (p=0.054)<br>#Features: 8	0.54<br>AUC:0.62 (p=0.296)<br>#Features: 32	0.67<br>AUC:0.59 (p=0.062)<br>#Features: 8	0.8<br>AUC:0.63 (p=0.318)<br>#Features: 16	0.61<br>AUC:0.62 (p=0.317)<br>#Features: 8		0.62<br>AUC:0.65 (p=0.673)<br>#Features: 4

Figure S17: Stability of the models on dataset Carvalho2018. The best model is framed with a blue border, models that were significantly different to the best model are not shown. Statistical significance was tested using a DeLong test.

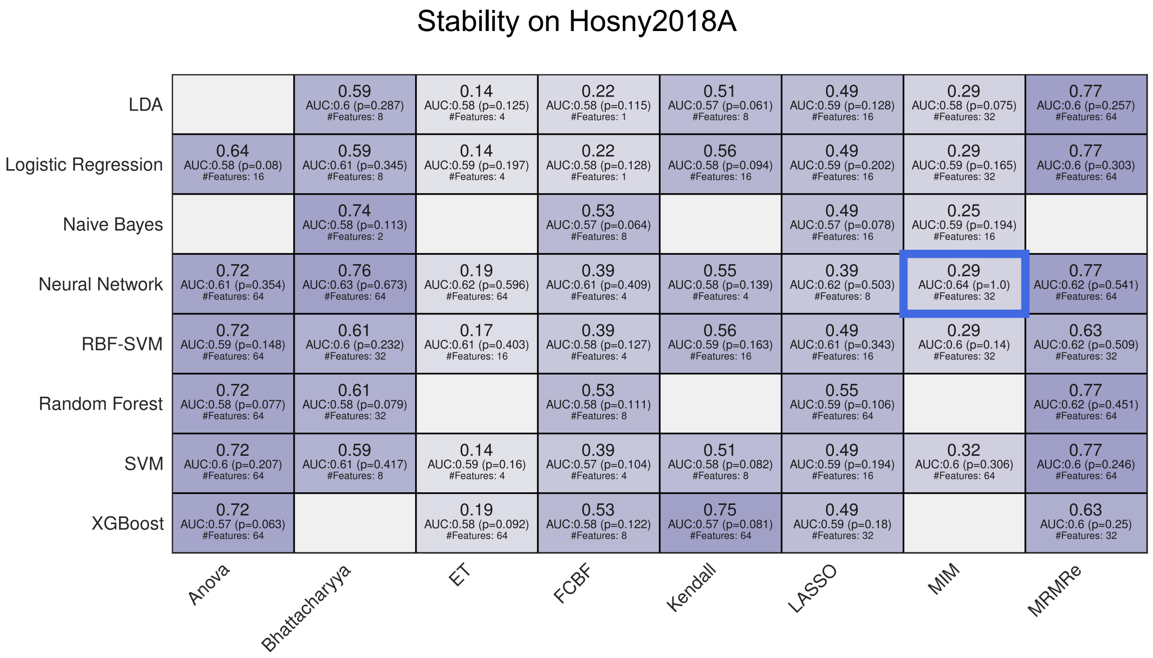

Figure S18: Stability of the models on dataset Hosny2018A. The best model is framed with a blue border, models that were significantly different to the best model are not shown. Statistical significance was tested using a DeLong test.

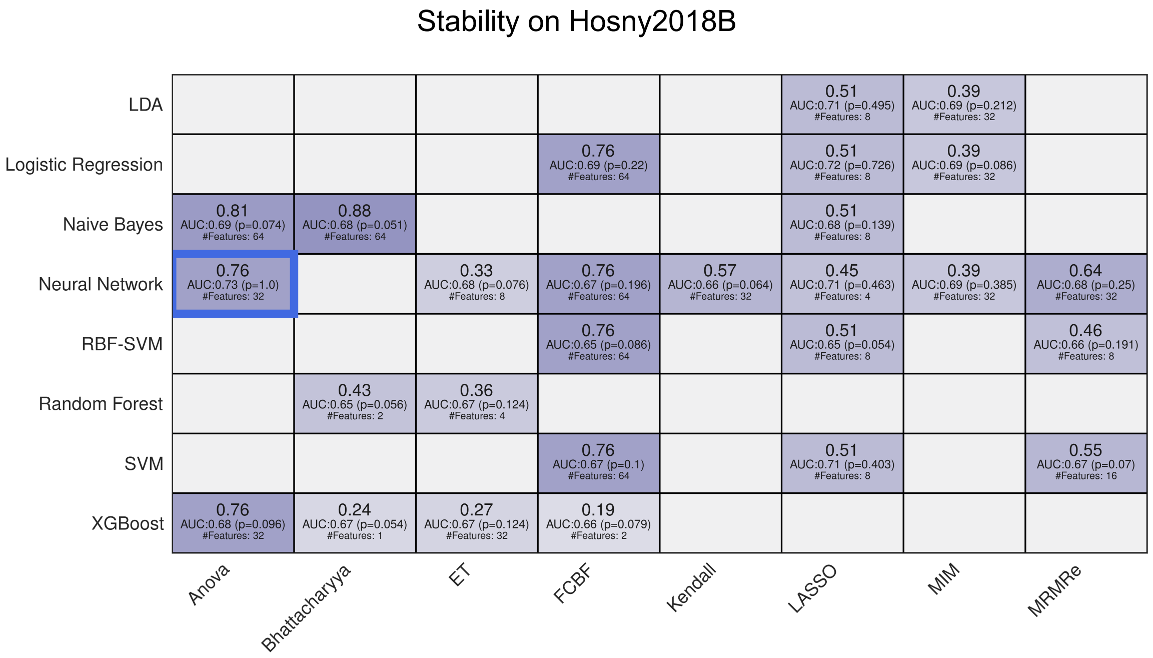

Figure S19: Stability of the models on dataset Hosny2018B. The best model is framed with a blue border, models that were significantly different to the best model are not shown. Statistical significance was tested using a DeLong test.

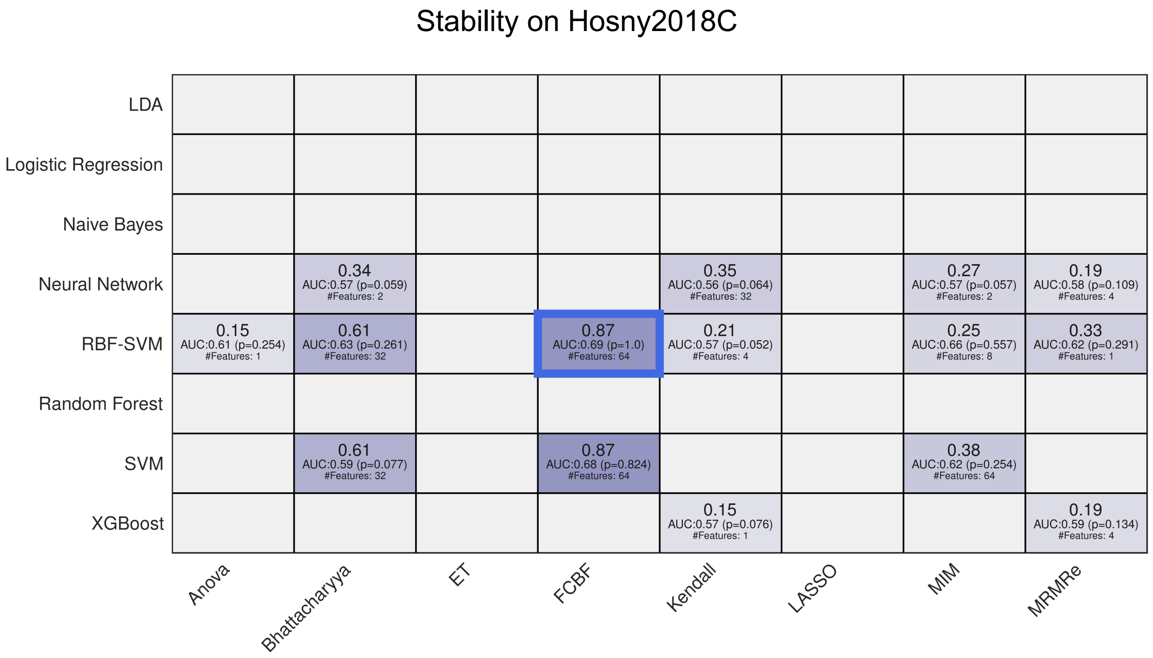

Figure S20: Stability of the models on dataset Hosny2018C. The best model is framed with a blue border, models that were significantly different to the best model are not shown. Statistical significance was tested using a DeLong test.

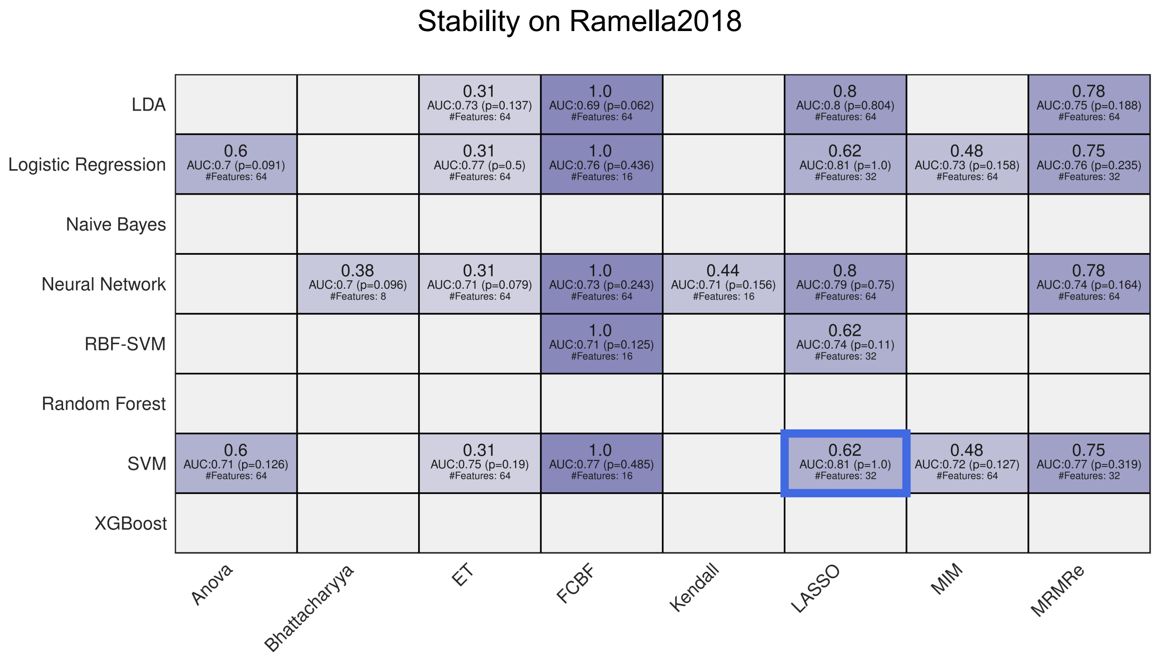

Figure S21: Stability of the models on dataset Ramella2018. The best model is framed with a blue border, models that were significantly different to the best model are not shown. Statistical significance was tested using a DeLong test.

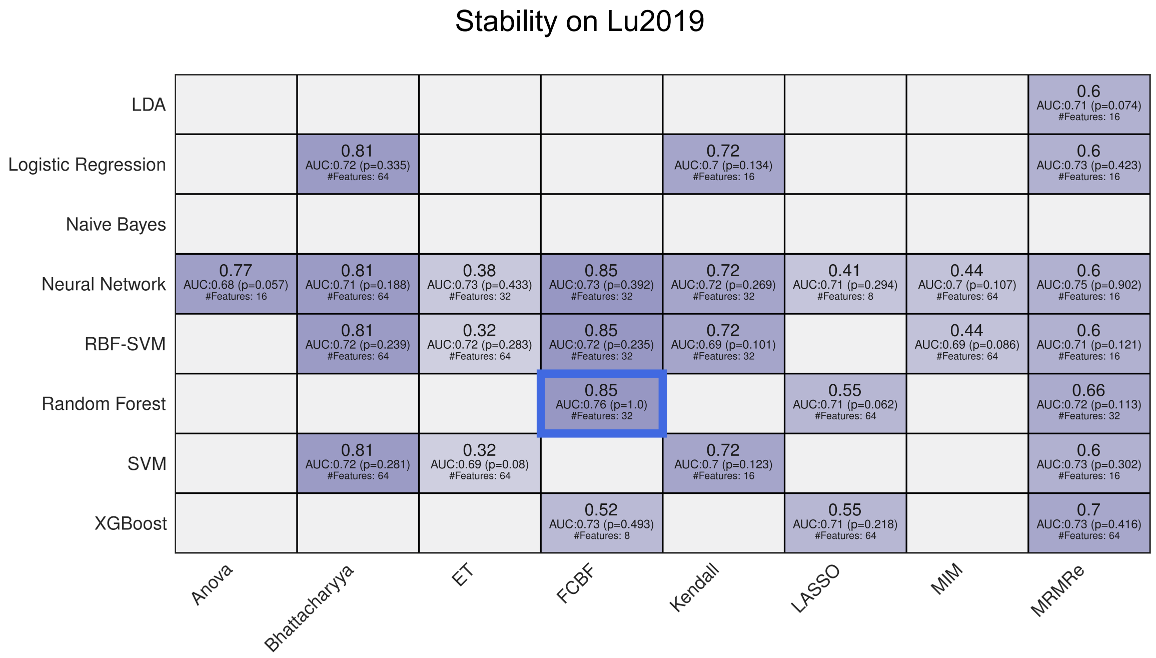

Figure S22: Stability of the models on dataset Lu2019. The best model is framed with a blue border, models that were significantly different to the best model are not shown. Statistical significance was tested using a DeLong test.

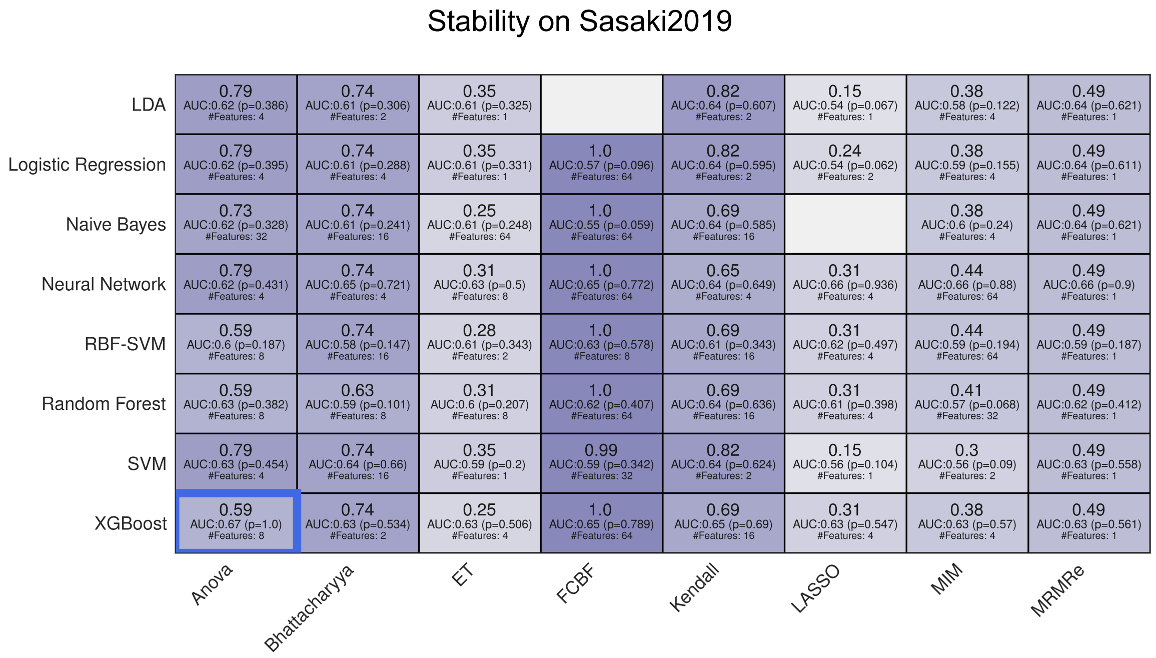

Figure S23: Stability of the models on dataset Sasaki2019. The best model is framed with a blue border, models that were significantly different to the best model are not shown. Statistical significance was tested using a DeLong test.

Stability on Toivonen2019

	Anova	Bhattacharyya	ET	FCBF	Kendall	LASSO	MIM	MRMRe
LDA	1.0<br>AUC:0.83 (p=0.525)<br>#Features: 1		0.18<br>AUC:0.82 (p=0.455)<br>#Features: 4		0.44<br>AUC:0.83 (p=0.411)<br>#Features: 32	0.44<br>AUC:0.77 (p=0.187)<br>#Features: 32	0.3<br>AUC:0.77 (p=0.175)<br>#Features: 16	0.54<br>AUC:0.78 (p=0.173)<br>#Features: 64
Logistic Regression	1.0<br>AUC:0.84 (p=0.619)<br>#Features: 1	0.68<br>AUC:0.73 (p=0.054)<br>#Features: 8	0.15<br>AUC:0.82 (p=0.455)<br>#Features: 16		0.36<br>AUC:0.83 (p=0.408)<br>#Features: 16	0.44<br>AUC:0.77 (p=0.188)<br>#Features: 32	0.3<br>AUC:0.78 (p=0.22)<br>#Features: 16	0.48<br>AUC:0.77 (p=0.114)<br>#Features: 32
Naive Bayes	1.0<br>AUC:0.82 (p=0.463)<br>#Features: 1	0.57<br>AUC:0.72 (p=0.052)<br>#Features: 64	0.18<br>AUC:0.76 (p=0.112)<br>#Features: 4			0.42<br>AUC:0.74 (p=0.059)<br>#Features: 16	0.18<br>AUC:0.79 (p=0.091)<br>#Features: 4	
Neural Network	1.0<br>AUC:0.83 (p=0.442)<br>#Features: 1	0.59<br>AUC:0.74 (p=0.097)<br>#Features: 32	0.15<br>AUC:0.83 (p=0.516)<br>#Features: 16		0.44<br>AUC:0.85 (p=0.744)<br>#Features: 32	0.44<br>AUC:0.81 (p=0.409)<br>#Features: 32	0.16<br>AUC:0.8 (p=0.314)<br>#Features: 2	0.54<br>AUC:0.82 (p=0.486)<br>#Features: 64
RBF-SVM	0.44<br>AUC:0.77 (p=0.071)<br>#Features: 8		0.18<br>AUC:0.79 (p=0.218)<br>#Features: 4			0.44<br>AUC:0.79 (p=0.253)<br>#Features: 32	0.3<br>AUC:0.72 (p=0.065)<br>#Features: 16	0.48<br>AUC:0.74 (p=0.061)<br>#Features: 32
Random Forest	1.0<br>AUC:0.85 (p=0.741)<br>#Features: 1		0.15<br>AUC:0.78 (p=0.175)<br>#Features: 16		0.37<br>AUC:0.83 (p=0.195)<br>#Features: 8			0.34<br>AUC:0.74 (p=0.057)<br>#Features: 4
SVM	1.0<br>AUC:0.84 (p=0.574)<br>#Features: 1		0.15<br>AUC:0.83 (p=0.48)<br>#Features: 16		0.44<br>AUC:0.86 (p=0.733)<br>#Features: 32	0.44<br>AUC:0.78 (p=0.243)<br>#Features: 32	0.27<br>AUC:0.79 (p=0.217)<br>#Features: 8	0.48<br>AUC:0.8 (p=0.302)<br>#Features: 32
XGBoost	1.0<br>AUC:0.83 (p=0.408)<br>#Features: 1		0.15<br>AUC:0.81 (p=0.359)<br>#Features: 16		0.37<br>AUC:0.87 (p=1.0)<br>#Features: 8	0.44<br>AUC:0.76 (p=0.117)<br>#Features: 32	0.3<br>AUC:0.76 (p=0.112)<br>#Features: 16	0.48<br>AUC:0.75 (p=0.085)<br>#Features: 32

Figure S24: Stability of the models on dataset Toivonen2019. The best model is framed with a blue border, models that were significantly different to the best model are not shown. Statistical significance was tested using a DeLong test.

Stability on Keek2020

	Anova	Bhattacharyya	ET	FCBF	Kendall	LASSO	MIM	MRMRe
LDA	0.33<br>AUC:0.65 (p=0.137)<br>#Features: 4	0.36<br>AUC:0.64 (p=0.117)<br>#Features: 1	0.33<br>AUC:0.65 (p=0.183)<br>#Features: 2		0.27<br>AUC:0.65 (p=0.191)<br>#Features: 1		0.3<br>AUC:0.68 (p=0.709)<br>#Features: 8	0.48<br>AUC:0.64 (p=0.109)<br>#Features: 8
Logistic Regression	0.61<br>AUC:0.66 (p=0.233)<br>#Features: 16	0.83<br>AUC:0.66 (p=0.27)<br>#Features: 64	0.28<br>AUC:0.66 (p=0.242)<br>#Features: 8	0.9<br>AUC:0.65 (p=0.126)<br>#Features: 64	0.27<br>AUC:0.65 (p=0.185)<br>#Features: 1	0.17<br>AUC:0.68 (p=0.145)<br>#Features: 2	0.3<br>AUC:0.68 (p=0.787)<br>#Features: 8	0.48<br>AUC:0.64 (p=0.147)<br>#Features: 8
Naive Bayes	0.7<br>AUC:0.65 (p=0.225)<br>#Features: 32	0.83<br>AUC:0.66 (p=0.356)<br>#Features: 64	0.31<br>AUC:0.66 (p=0.299)<br>#Features: 32	0.9<br>AUC:0.64 (p=0.154)<br>#Features: 64	0.64<br>AUC:0.65 (p=0.151)<br>#Features: 64	0.23<br>AUC:0.65 (p=0.137)<br>#Features: 4	0.3<br>AUC:0.67 (p=0.629)<br>#Features: 8	
Neural Network	0.33<br>AUC:0.66 (p=0.338)<br>#Features: 4	0.36<br>AUC:0.65 (p=0.213)<br>#Features: 1	0.33<br>AUC:0.65 (p=0.134)<br>#Features: 1		0.49<br>AUC:0.65 (p=0.195)<br>#Features: 16	0.17<br>AUC:0.68 (p=0.56)<br>#Features: 2	0.3<br>AUC:0.67 (p=0.655)<br>#Features: 8	0.63<br>AUC:0.64 (p=0.21)<br>#Features: 32
RBF-SVM	0.72<br>AUC:0.64 (p=0.063)<br>#Features: 64	0.36<br>AUC:0.65 (p=0.213)<br>#Features: 1	0.29<br>AUC:0.63 (p=0.054)<br>#Features: 64	0.9<br>AUC:0.63 (p=0.053)<br>#Features: 64	0.31<br>AUC:0.65 (p=0.153)<br>#Features: 4	0.17<br>AUC:0.66 (p=0.107)<br>#Features: 2	0.3<br>AUC:0.66 (p=0.403)<br>#Features: 8	0.73<br>AUC:0.67 (p=0.459)<br>#Features: 64
Random Forest	0.72<br>AUC:0.64 (p=0.18)<br>#Features: 64	0.83<br>AUC:0.67 (p=0.466)<br>#Features: 64	0.28<br>AUC:0.64 (p=0.168)<br>#Features: 8		0.64<br>AUC:0.66 (p=0.441)<br>#Features: 64	0.37<br>AUC:0.64 (p=0.074)<br>#Features: 32	0.4<br>AUC:0.64 (p=0.118)<br>#Features: 32	0.73<br>AUC:0.68 (p=0.725)<br>#Features: 64
SVM	0.61<br>AUC:0.66 (p=0.374)<br>#Features: 16	0.36<br>AUC:0.65 (p=0.143)<br>#Features: 1	0.33<br>AUC:0.65 (p=0.247)<br>#Features: 2		0.31<br>AUC:0.65 (p=0.221)<br>#Features: 4	0.17<br>AUC:0.69 (p=1.0)<br>#Features: 2	0.3<br>AUC:0.68 (p=0.715)<br>#Features: 8	0.48<br>AUC:0.64 (p=0.162)<br>#Features: 8
XGBoost	0.61<br>AUC:0.65 (p=0.28)<br>#Features: 16	0.5<br>AUC:0.63 (p=0.132)<br>#Features: 16	0.28<br>AUC:0.65 (p=0.305)<br>#Features: 8		0.35<br>AUC:0.65 (p=0.236)<br>#Features: 8	0.37<br>AUC:0.63 (p=0.069)<br>#Features: 32	0.4<br>AUC:0.66 (p=0.445)<br>#Features: 32	0.73<br>AUC:0.64 (p=0.156)<br>#Features: 64

Figure S25: Stability of the models on dataset Keek2020. The best model is framed with a blue border, models that were significantly different to the best model are not shown. Statistical significance was tested using a DeLong test.

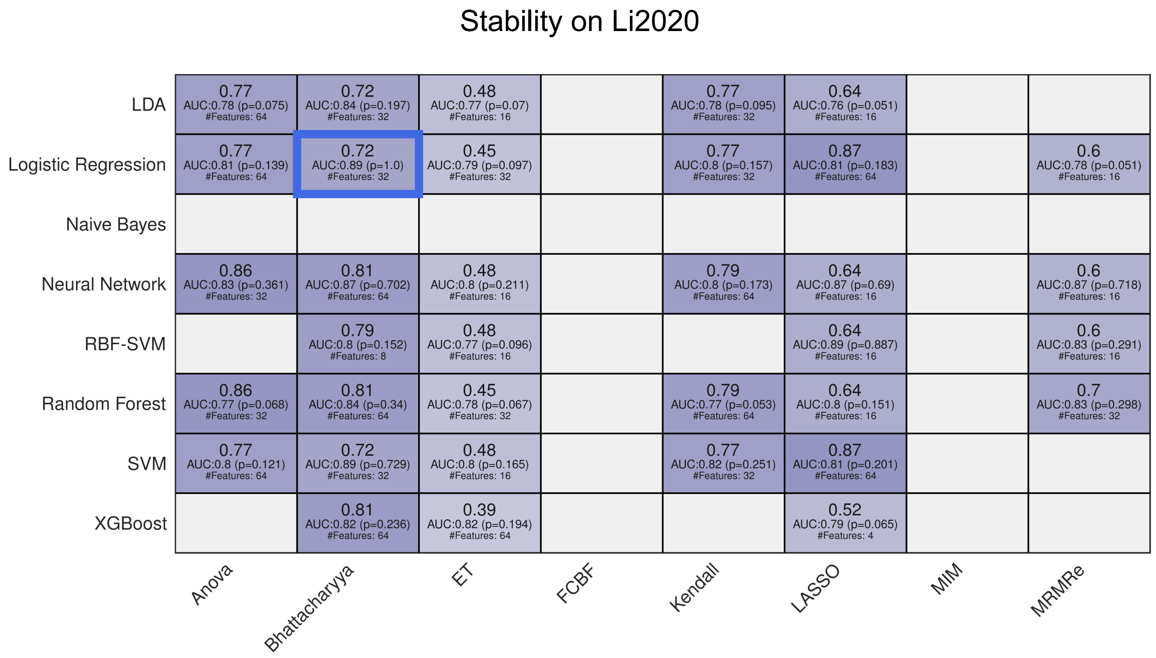

Figure S26: Stability of the models on dataset Li2020. The best model is framed with a blue border, models that were significantly different to the best model are not shown. Statistical significance was tested using a DeLong test.

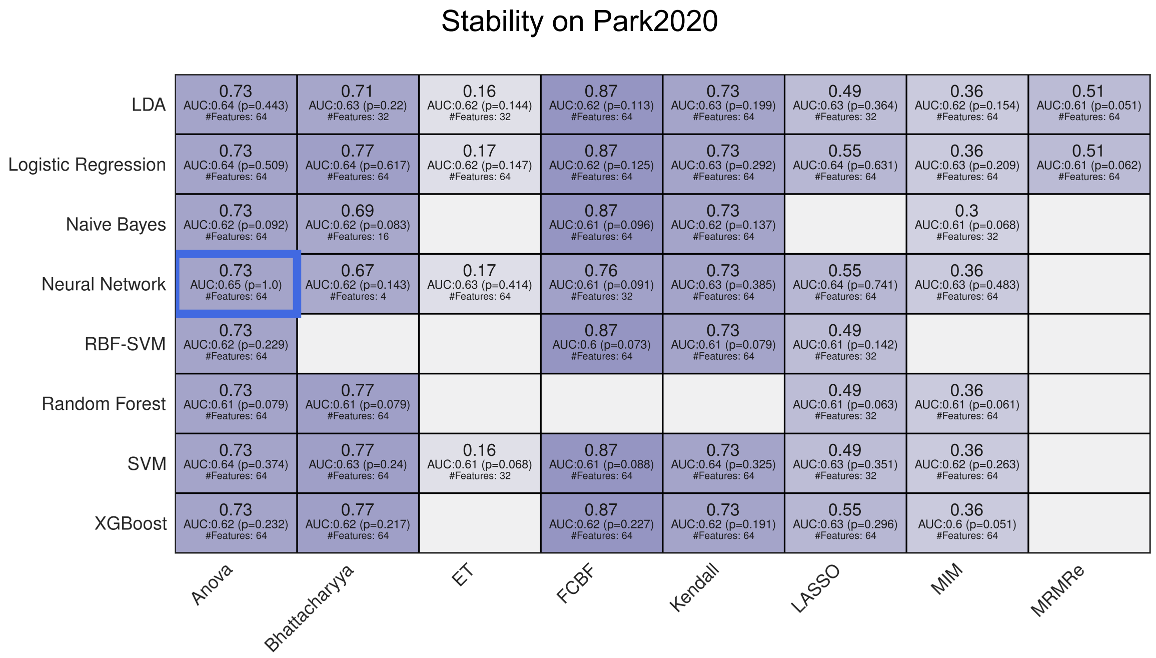

Figure S27: Stability of the models on dataset Park2020. The best model is framed with a blue border, models that were significantly different to the best model are not shown. Statistical significance was tested using a DeLong test.

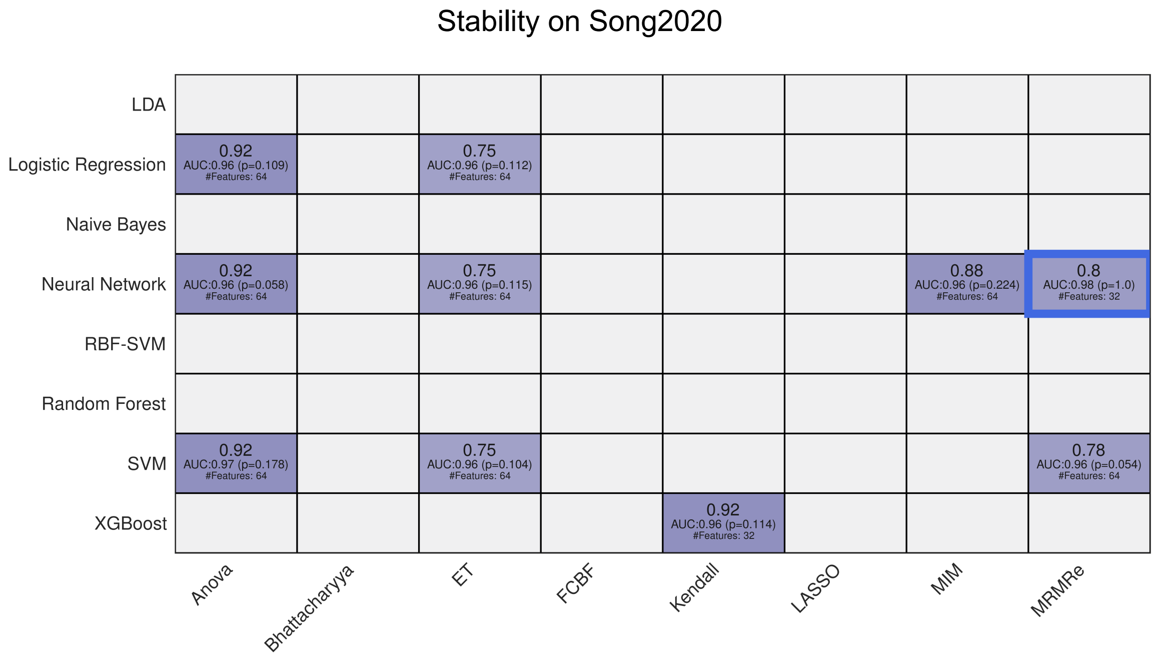

Figure S28: Stability of the models on dataset Song2020. The best model is framed with a blue border, models that were significantly different to the best model are not shown. Statistical significance was tested using a DeLong test.

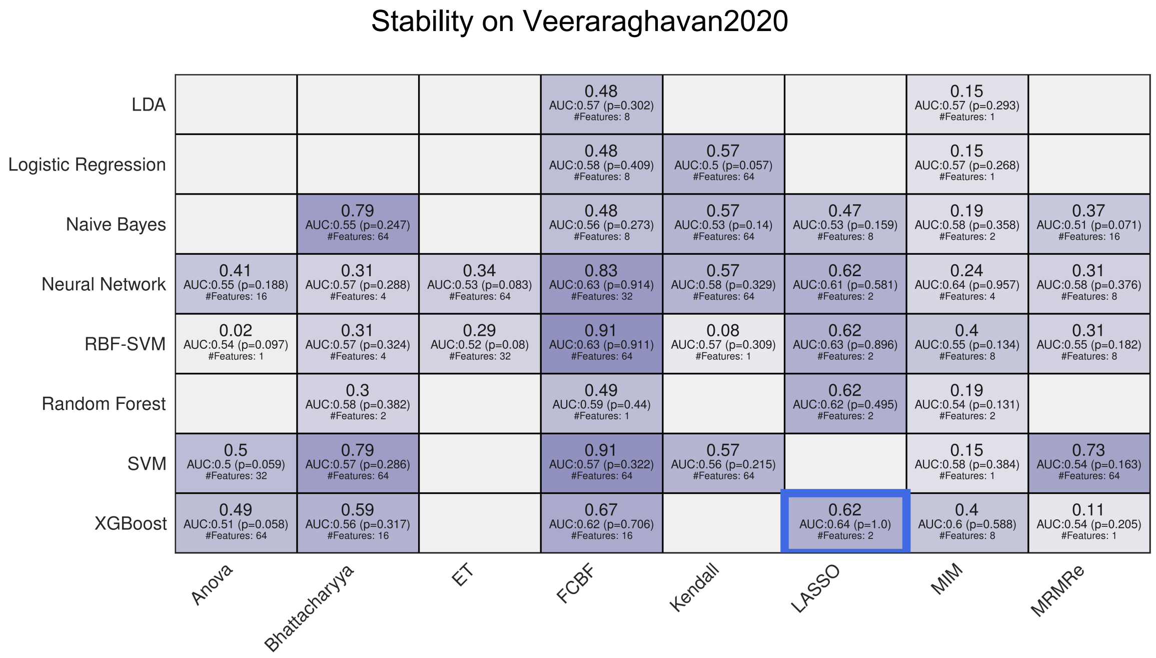

Figure S29: Stability of the models on dataset Veeraraghavan2020. The best model is framed with a blue border, models that were significantly different to the best model are not shown. Statistical significance was tested using a DeLong test.
