# Supplementary material for: Evaluation of the dependence of radiomic features on the machine learning model
Source: Insights Imaging. 2022 Feb 24;13:28. doi: 10.1186/s13244-022-01170-2 (PMC8873309; doi:10.1186/s13244-022-01170-2)
Supplement: Supplementary file 4 — Additional file 4. Results for the similarity of feature selection methods for each data set. [file 13244_2022_1170_MOESM4_ESM.pdf]

Supplemental 4

Feature Similarity

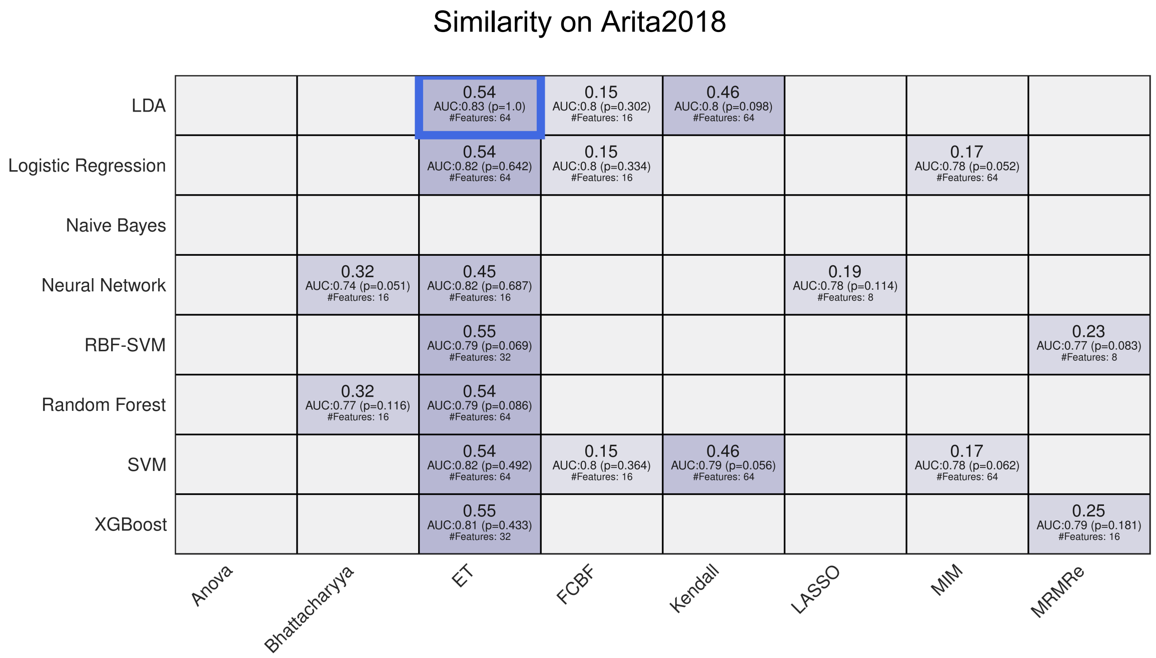

Figure S30: Similarity of the models on dataset Arita2018. The best model is framed with a blue border, models that were significantly different to the best model are not shown. Statistical significance was tested using a DeLong test.

Similarity on Carvalho2018

	Anova	Bhattacharyya	ET	FCBF	Kendall	LASSO	MIM	MRMRe
LDA	0.16 AUC:0.62 (p=0.238) #Features: 4	-0.05 AUC:0.62 (p=0.223) #Features: 2	0.18 AUC:0.61 (p=0.152) #Features: 4		0.15 AUC:0.62 (p=0.207) #Features: 4		0.02 AUC:0.6 (p=0.144) #Features: 4	0.23 AUC:0.61 (p=0.159) #Features: 2
Logistic Regression	0.23 AUC:0.62 (p=0.214) #Features: 8	-0.05 AUC:0.62 (p=0.228) #Features: 2	0.18 AUC:0.61 (p=0.144) #Features: 4		0.16 AUC:0.62 (p=0.221) #Features: 8	0.26 AUC:0.61 (p=0.128) #Features: 16	0.02 AUC:0.61 (p=0.166) #Features: 4	0.23 AUC:0.62 (p=0.21) #Features: 2
Naive Bayes	0.16 AUC:0.62 (p=0.211) #Features: 16	-0.05 AUC:0.61 (p=0.145) #Features: 2	0.27 AUC:0.61 (p=0.131) #Features: 16		0.11 AUC:0.62 (p=0.229) #Features: 16			0.23 AUC:0.61 (p=0.189) #Features: 2
Neural Network	0.11 AUC:0.65 (p=0.547) #Features: 64	-0.01 AUC:0.64 (p=0.474) #Features: 64	0.23 AUC:0.65 (p=0.746) #Features: 8	0.03 AUC:0.63 (p=0.339) #Features: 16	0.16 AUC:0.65 (p=0.748) #Features: 8	0.26 AUC:0.66 (p=0.848) #Features: 16	0.07 AUC:0.63 (p=0.303) #Features: 64	0.81 AUC:0.67 (p=1.0) #Features: 16
RBF-SVM	0.16 AUC:0.65 (p=0.612) #Features: 4	-0.05 AUC:0.61 (p=0.215) #Features: 2	0.18 AUC:0.6 (p=0.116) #Features: 4		0.15 AUC:0.65 (p=0.642) #Features: 4	0.26 AUC:0.62 (p=0.195) #Features: 16	-0.01 AUC:0.59 (p=0.093) #Features: 2	0.81 AUC:0.66 (p=0.705) #Features: 16
Random Forest	0.23 AUC:0.62 (p=0.202) #Features: 8	0.01 AUC:0.62 (p=0.218) #Features: 8	0.27 AUC:0.6 (p=0.085) #Features: 16		0.16 AUC:0.63 (p=0.327) #Features: 8		0.1 AUC:0.6 (p=0.055) #Features: 32	0.36 AUC:0.65 (p=0.59) #Features: 4
SVM	0.16 AUC:0.62 (p=0.208) #Features: 4	-0.05 AUC:0.62 (p=0.242) #Features: 2	0.18 AUC:0.61 (p=0.138) #Features: 4		0.15 AUC:0.62 (p=0.204) #Features: 4	0.26 AUC:0.61 (p=0.129) #Features: 16	0.02 AUC:0.6 (p=0.14) #Features: 4	0.23 AUC:0.62 (p=0.218) #Features: 2
XGBoost	0.16 AUC:0.62 (p=0.254) #Features: 4	0.01 AUC:0.59 (p=0.054) #Features: 8	0.17 AUC:0.62 (p=0.296) #Features: 32	0.05 AUC:0.59 (p=0.062) #Features: 8	0.11 AUC:0.63 (p=0.318) #Features: 16	0.18 AUC:0.62 (p=0.317) #Features: 8		0.36 AUC:0.65 (p=0.673) #Features: 4

Figure S31: Similarity of the models on dataset Carvalho2018. The best model is framed with a blue border, models that were significantly different to the best model are not shown. Statistical significance was tested using a DeLong test.

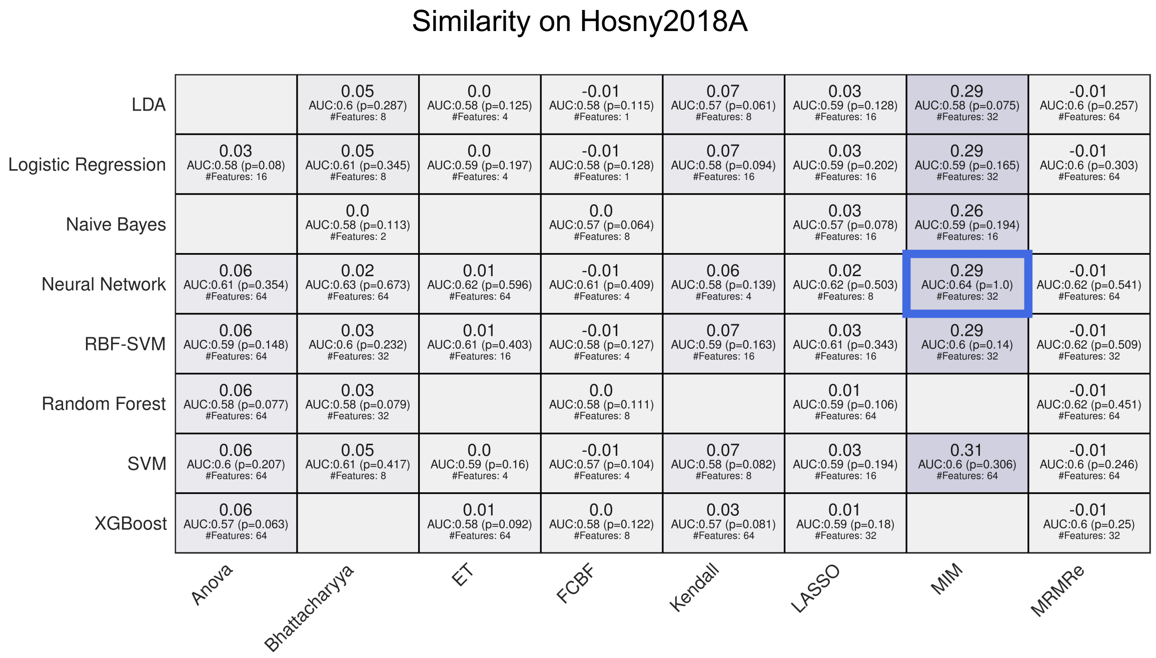

Figure S32: Similarity of the models on dataset Hosny2018A. The best model is framed with a blue border, models that were significantly different to the best model are not shown. Statistical significance was tested using a DeLong test.

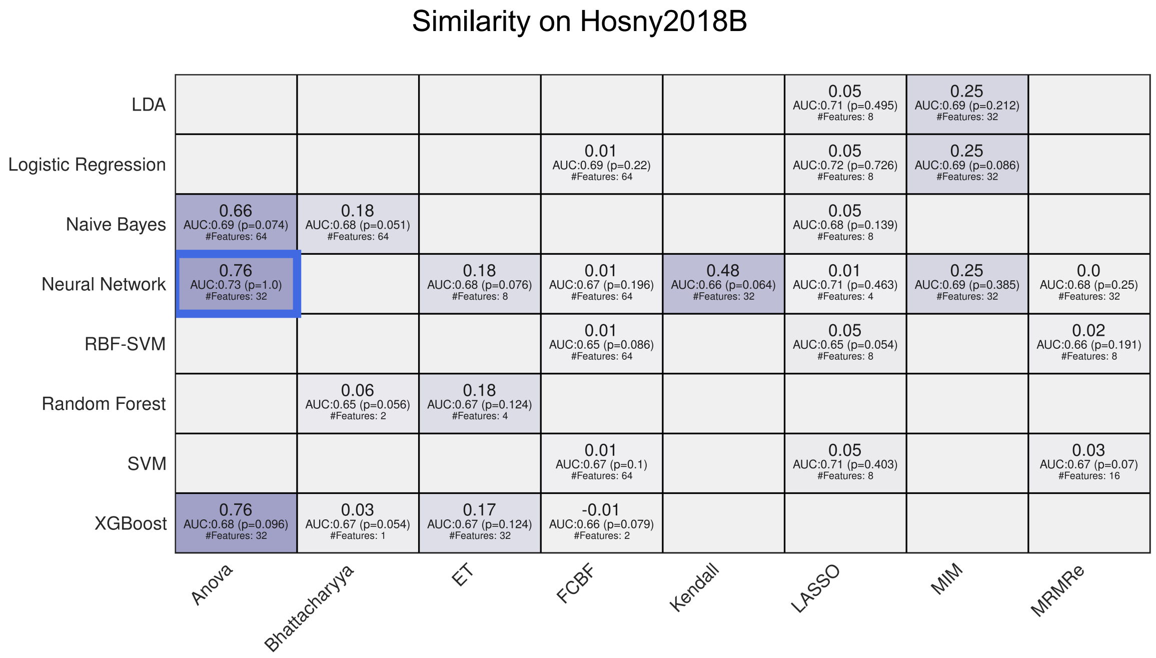

Figure S33: Similarity of the models on dataset Hosny2018B. The best model is framed with a blue border, models that were significantly different to the best model are not shown. Statistical significance was tested using a DeLong test.

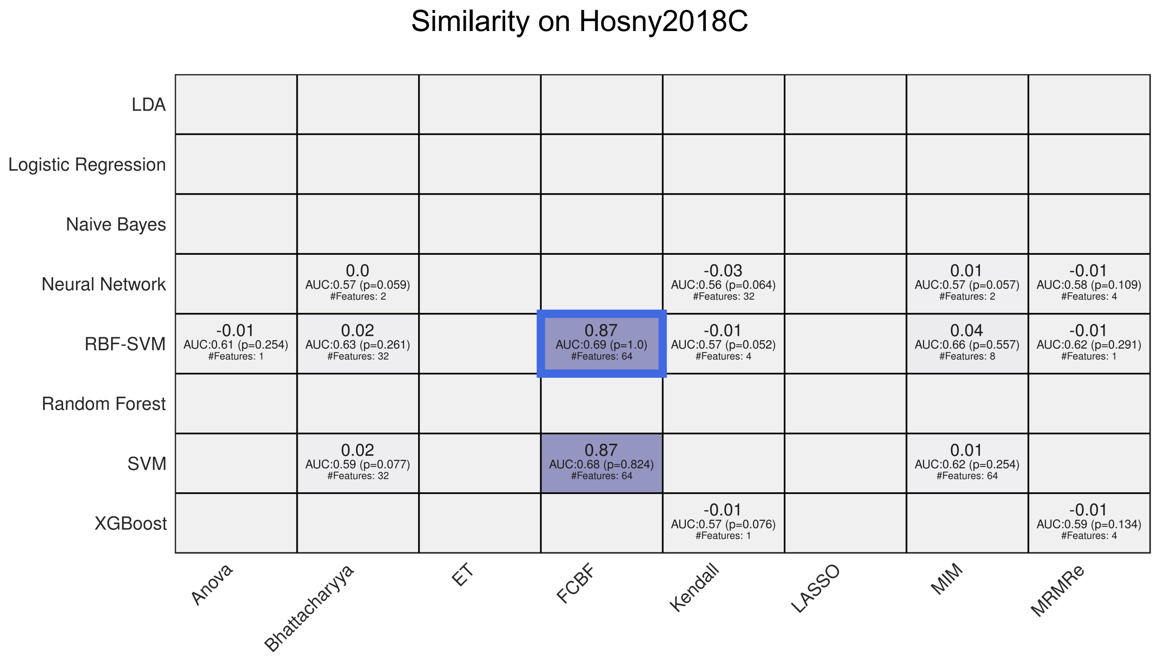

Figure S34: Similarity of the models on dataset Hosny2018C. The best model is framed with a blue border, models that were significantly different to the best model are not shown. Statistical significance was tested using a DeLong test.

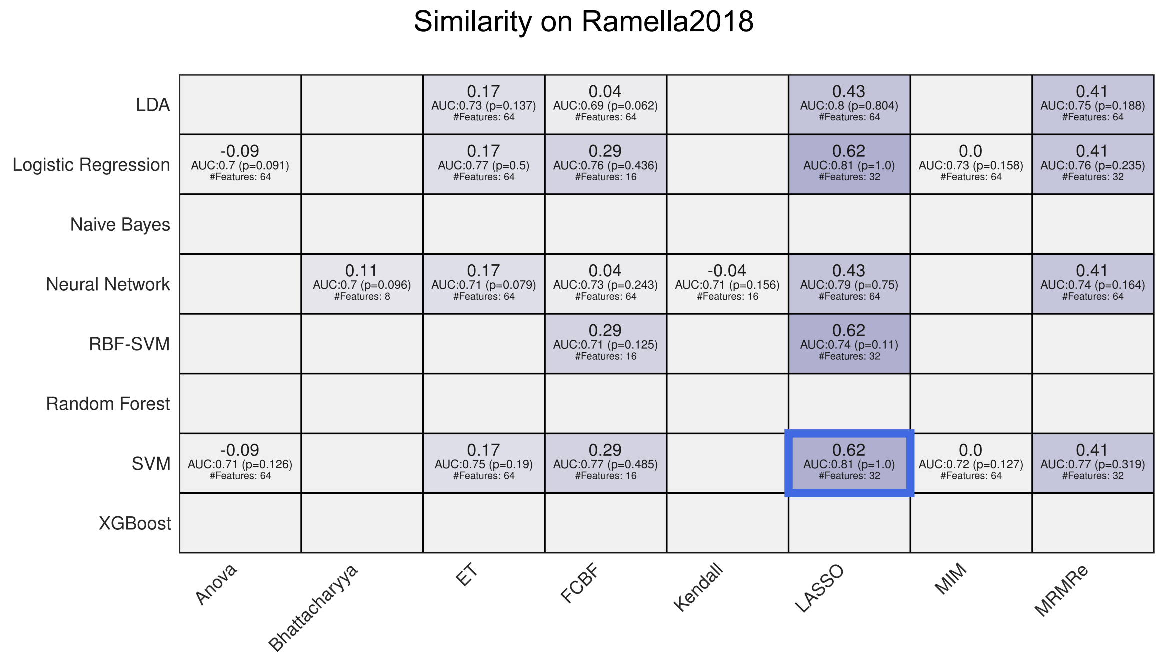

Figure S35: Similarity of the models on dataset Ramella2018. The best model is framed with a blue border, models that were significantly different to the best model are not shown. Statistical significance was tested using a DeLong test.

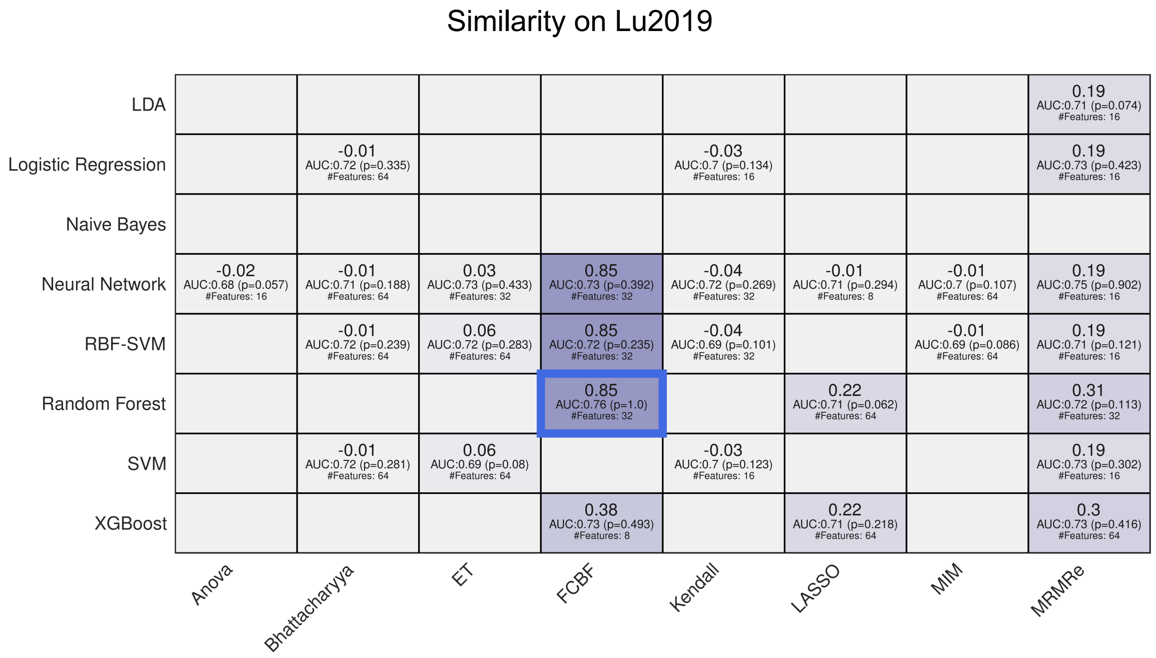

Figure S36: Similarity of the models on dataset Lu2019. The best model is framed with a blue border, models that were significantly different to the best model are not shown. Statistical significance was tested using a DeLong test.

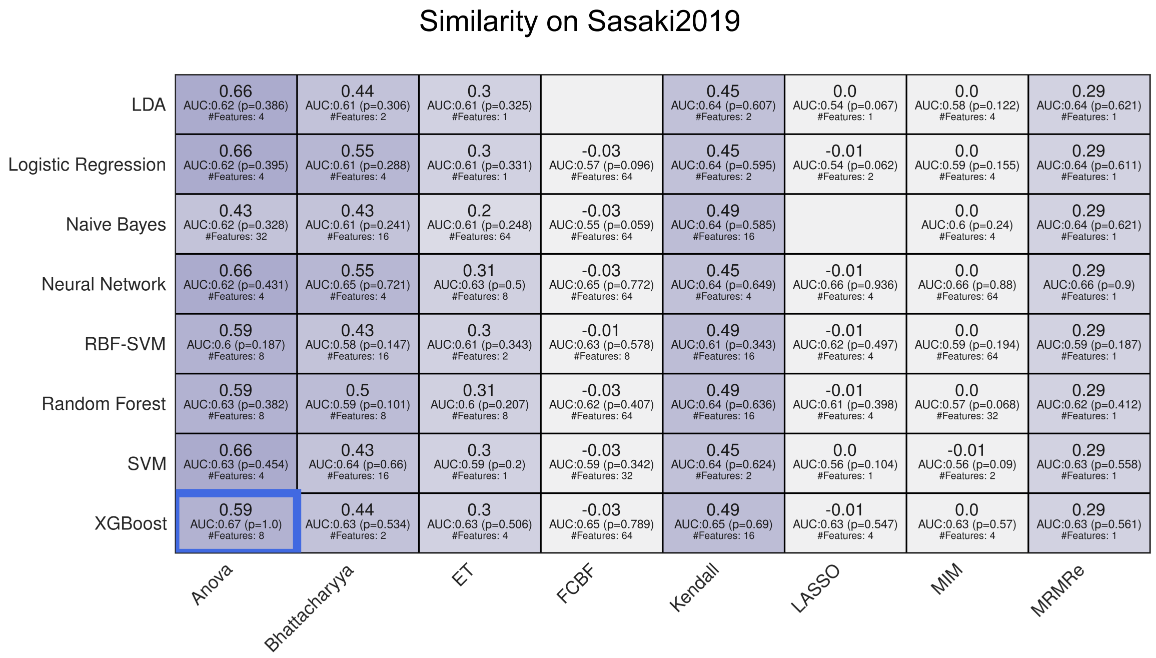

Figure S37: Similarity of the models on dataset Sasaki2019. The best model is framed with a blue border, models that were significantly different to the best model are not shown. Statistical significance was tested using a DeLong test.

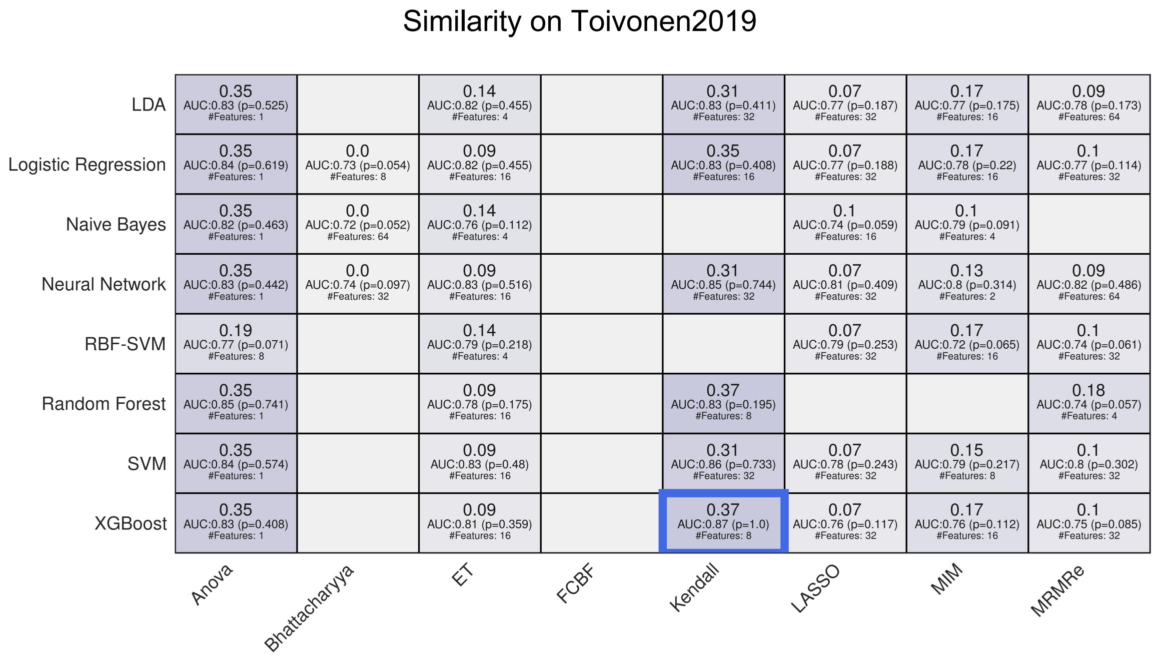

Figure S38: Similarity of the models on dataset Toivonen2019. The best model is framed with a blue border, models that were significantly different to the best model are not shown. Statistical significance was tested using a DeLong test.

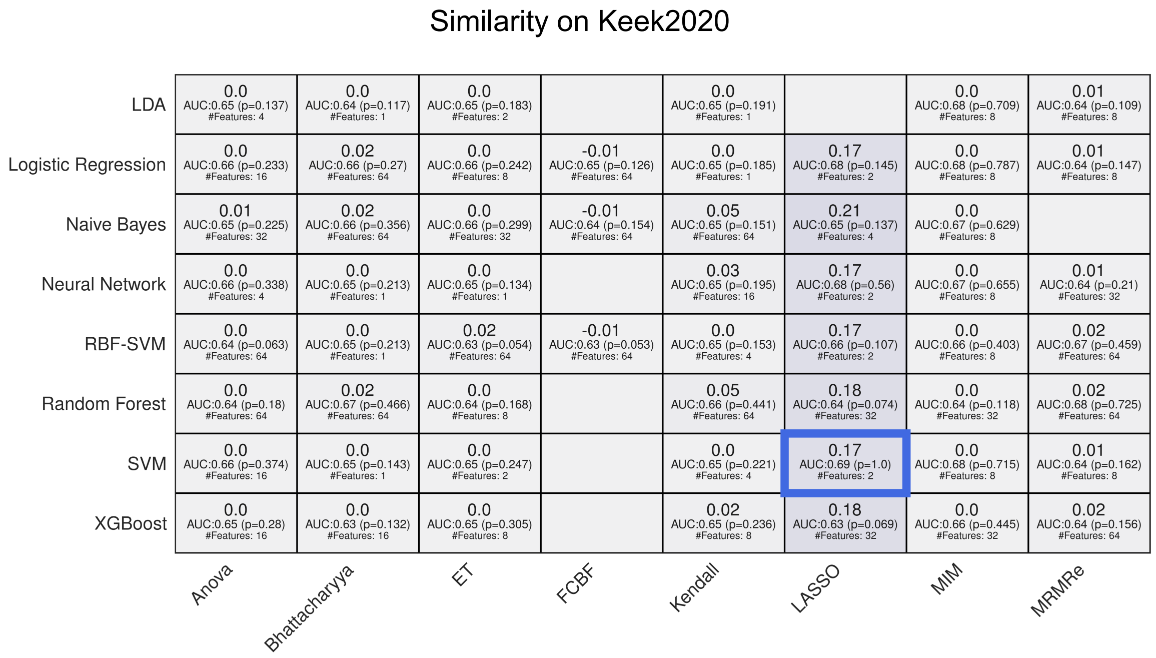

Figure S39: Similarity of the models on dataset Keek2020. The best model is framed with a blue border, models that were significantly different to the best model are not shown. Statistical significance was tested using a DeLong test.

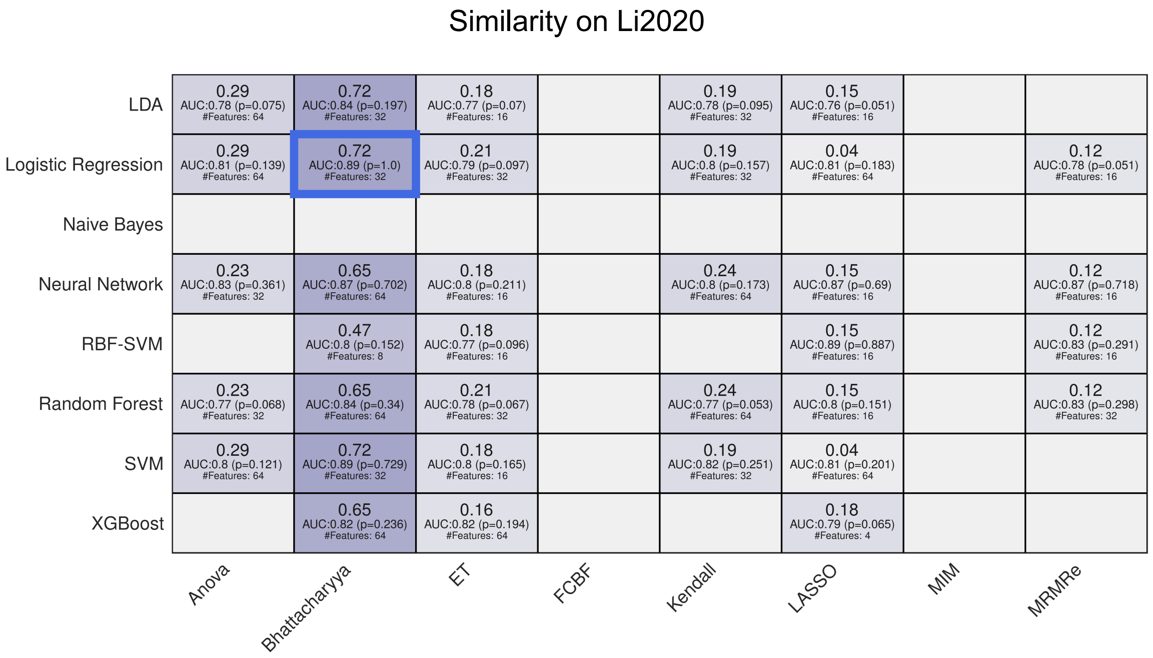

Figure S40: Similarity of the models on dataset Li2020. The best model is framed with a blue border, models that were significantly different to the best model are not shown. Statistical significance was tested using a DeLong test.

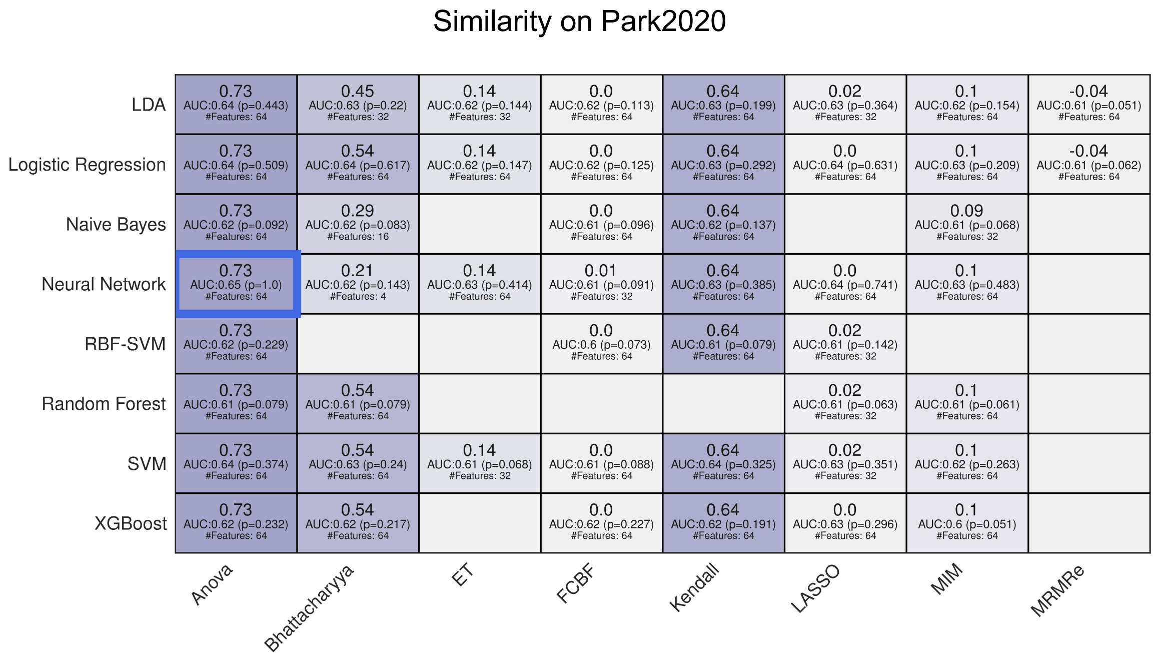

Figure S41: Similarity of the models on dataset Park2020. The best model is framed with a blue border, models that were significantly different to the best model are not shown. Statistical significance was tested using a DeLong test.

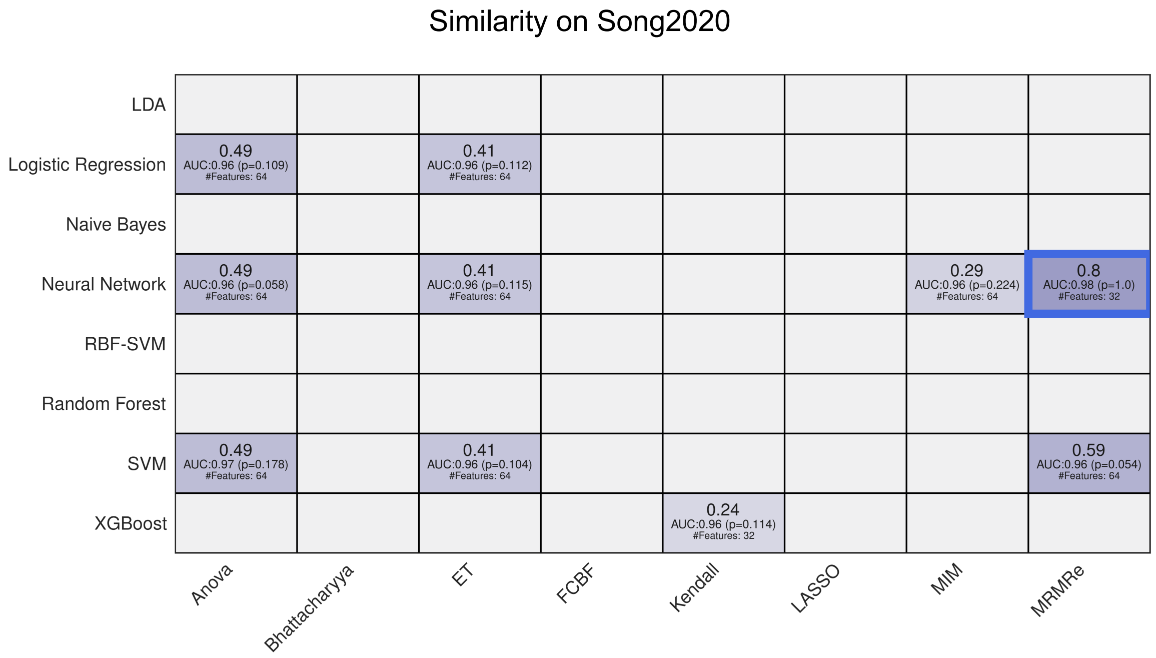

Figure S42: Similarity of the models on dataset Song2020. The best model is framed with a blue border, models that were significantly different to the best model are not shown. Statistical significance was tested using a DeLong test.

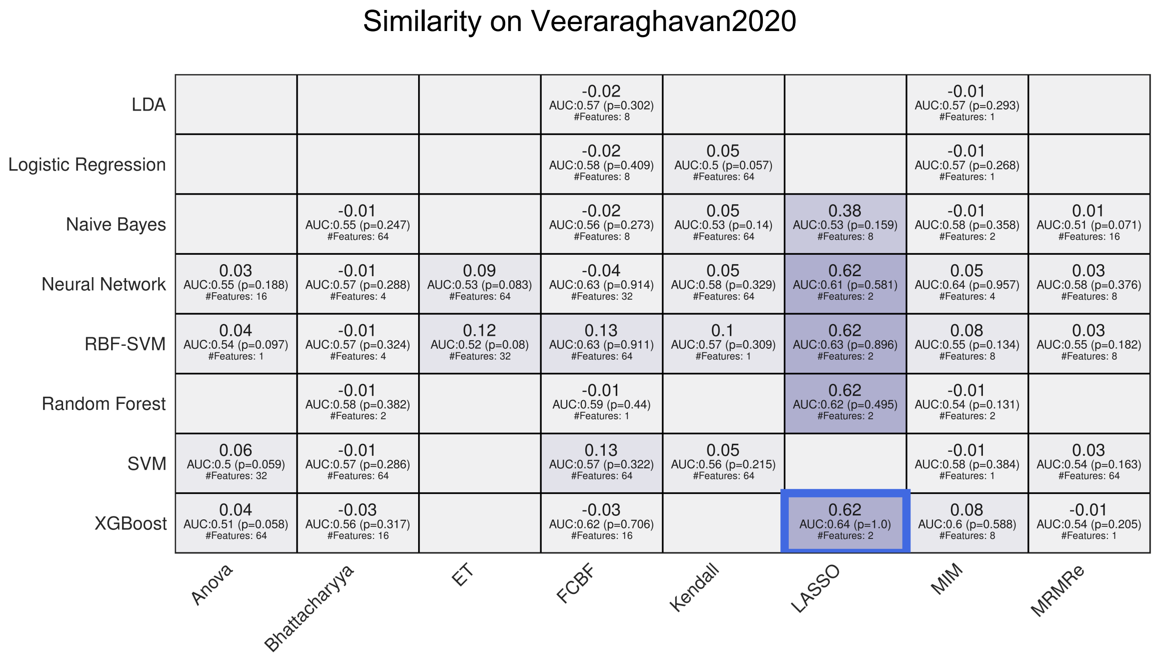

Figure S43: Similarity of the models on dataset Veeraraghavan2020. The best model is framed with a blue border, models that were significantly different to the best model are not shown. Statistical significance was tested using a DeLong test.
