# Supplementary material for: Evaluation of the dependence of radiomic features on the machine learning model
Source: Insights Imaging. 2022 Feb 24;13:28. doi: 10.1186/s13244-022-01170-2 (PMC8873309; doi:10.1186/s13244-022-01170-2)
Supplement: Supplementary file 5 — Additional file 5. Results for the correlation of the selected features for each data set. [file 13244_2022_1170_MOESM5_ESM.pdf]

Supplemental 5

Feature Correlation

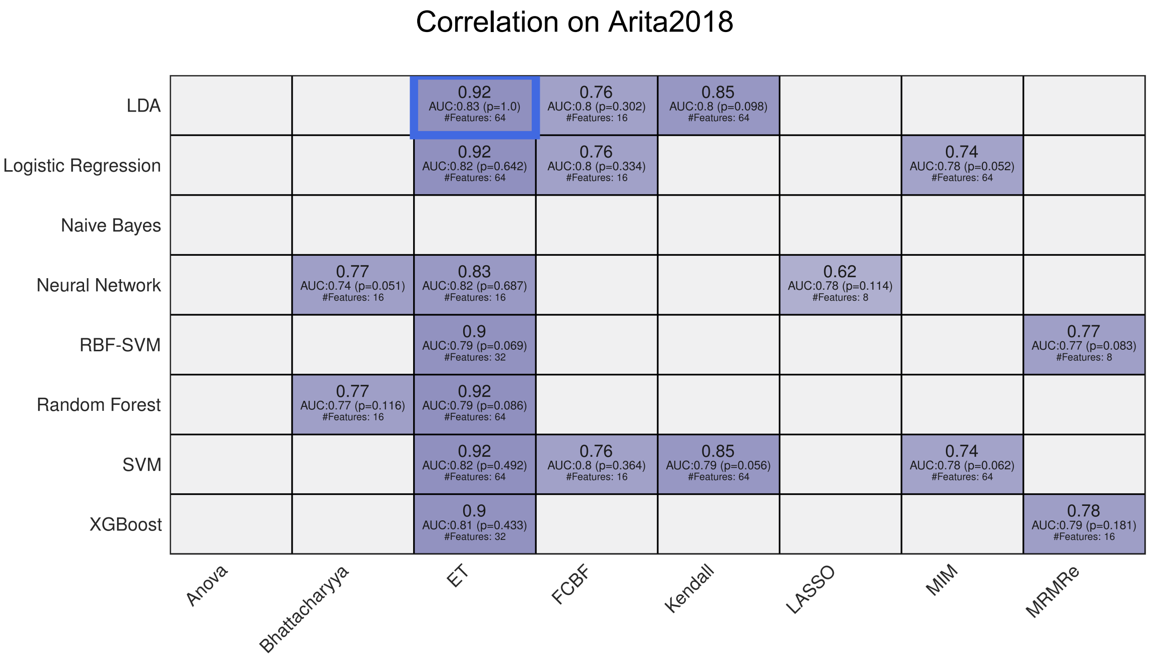

Figure S44: Correlation of the models on dataset Arita2018. The best model is framed with a blue border, models that were significantly different to the best model are not shown. Statistical significance was tested using a DeLong test.

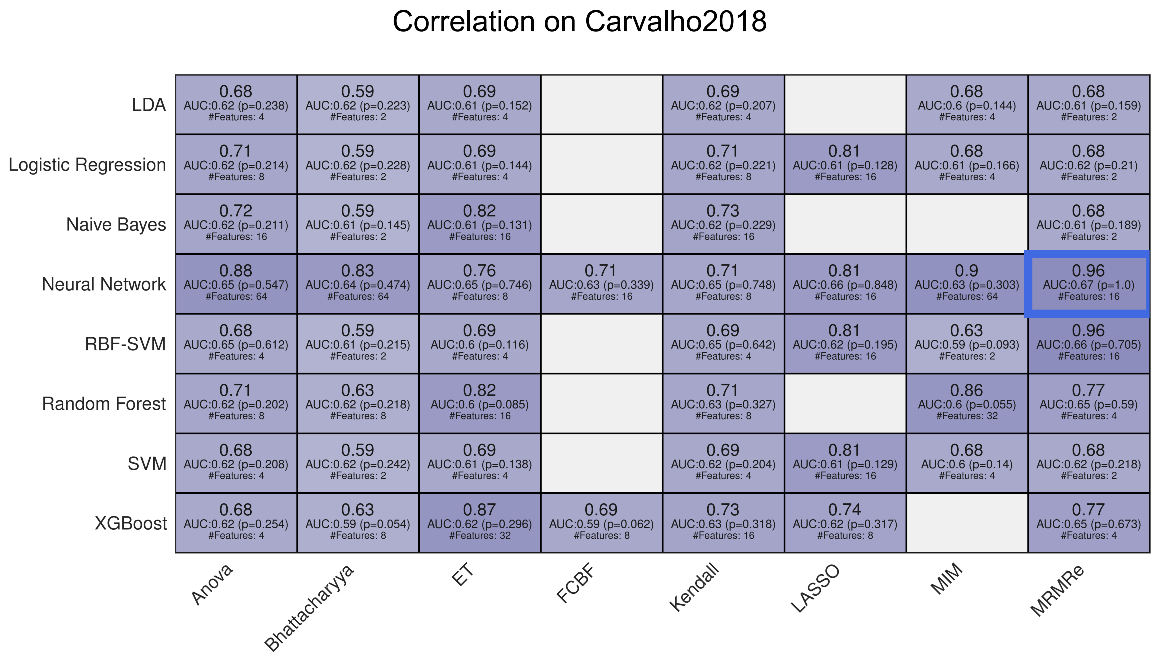

Figure S45: Correlation of the models on dataset Carvalho2018. The best model is framed with a blue border, models that were significantly different to the best model are not shown. Statistical significance was tested using a DeLong test.

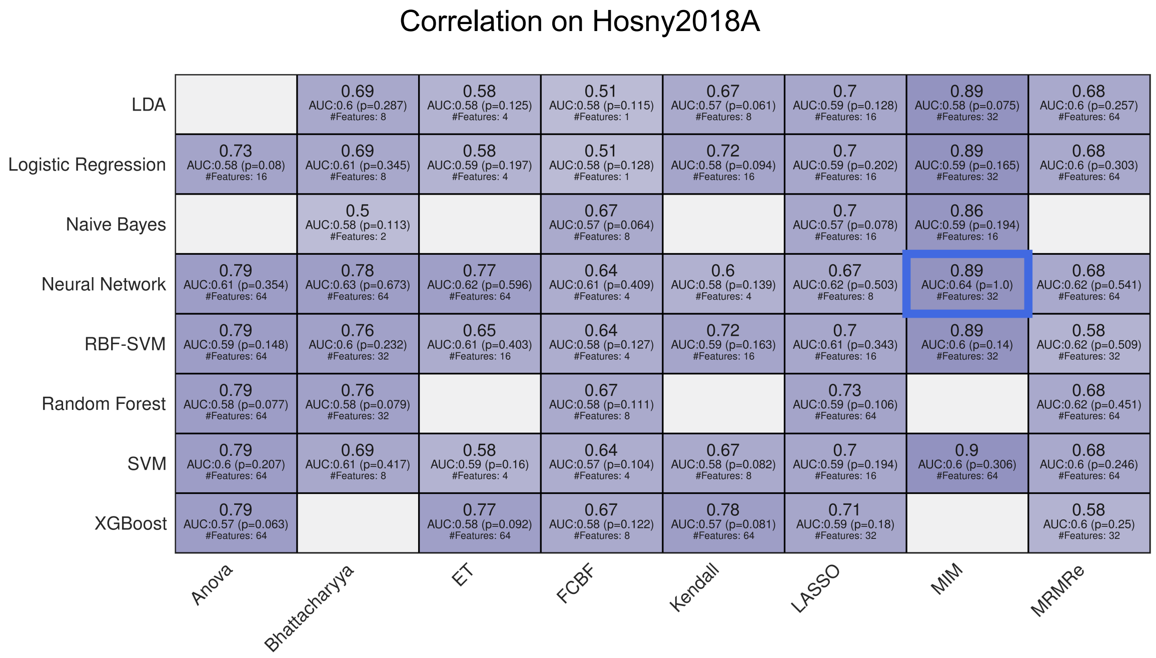

Figure S46: Correlation of the models on dataset Hosny2018A. The best model is framed with a blue border, models that were significantly different to the best model are not shown. Statistical significance was tested using a DeLong test.

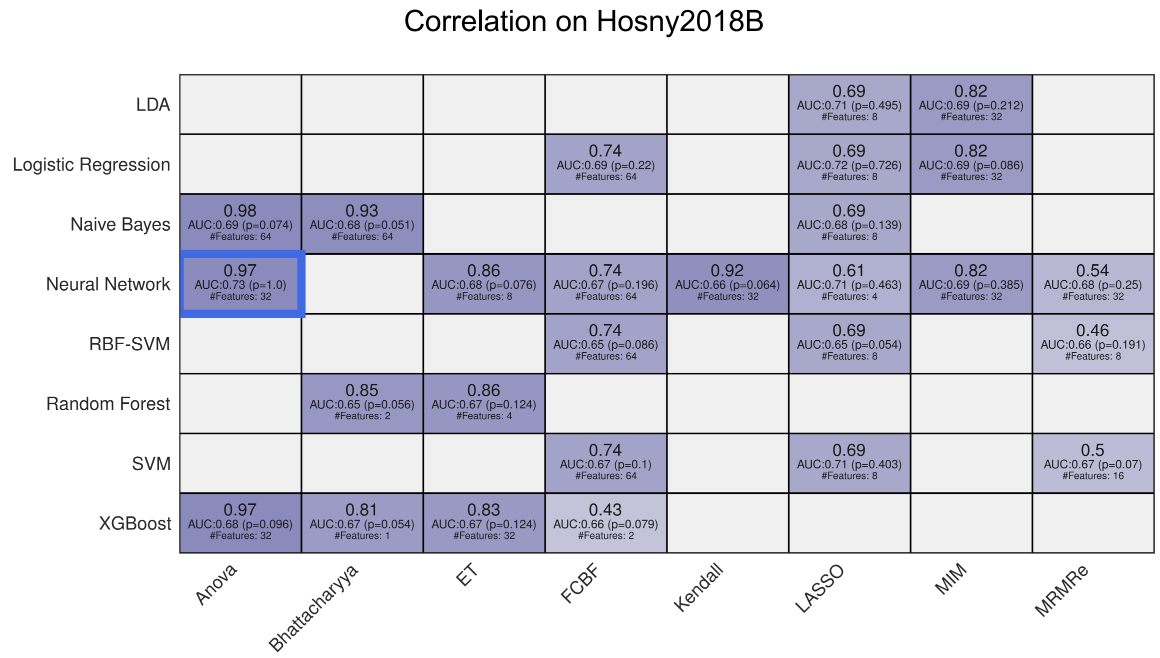

Figure S47: Correlation of the models on dataset Hosny2018B. The best model is framed with a blue border, models that were significantly different to the best model are not shown. Statistical significance was tested using a DeLong test.

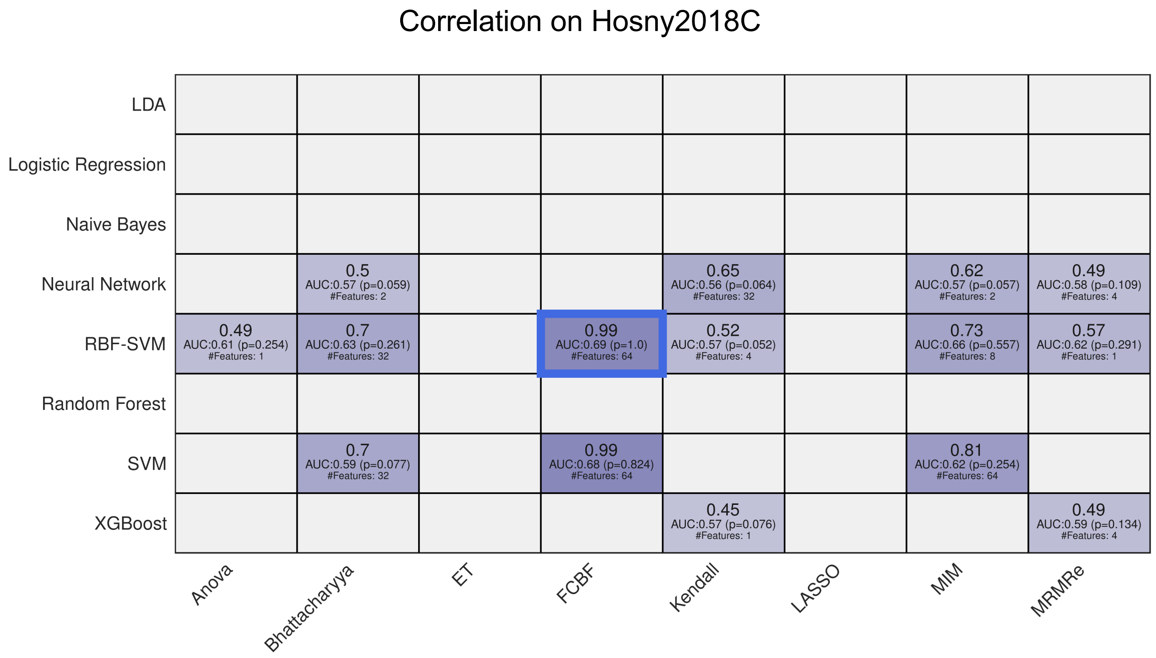

Figure S48: Correlation of the models on dataset Hosny2018C. The best model is framed with a blue border, models that were significantly different to the best model are not shown. Statistical significance was tested using a DeLong test.

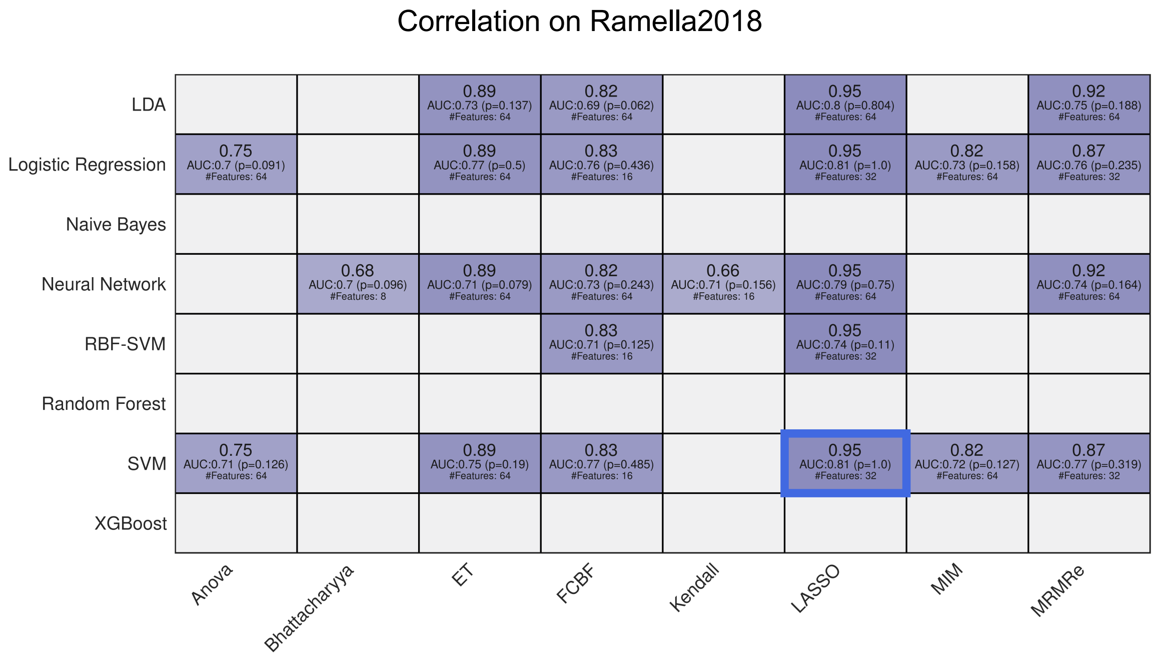

Figure S49: Correlation of the models on dataset Ramella2018. The best model is framed with a blue border, models that were significantly different to the best model are not shown. Statistical significance was tested using a DeLong test.

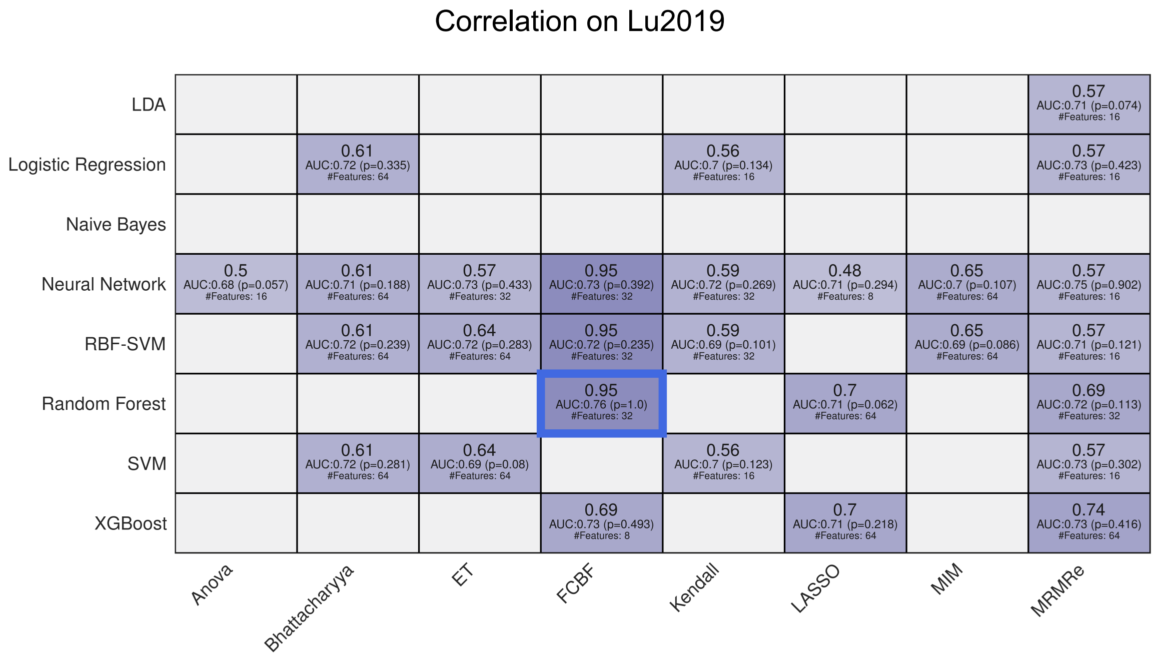

Figure S50: Correlation of the models on dataset Lu2019. The best model is framed with a blue border, models that were significantly different to the best model are not shown. Statistical significance was tested using a DeLong test.

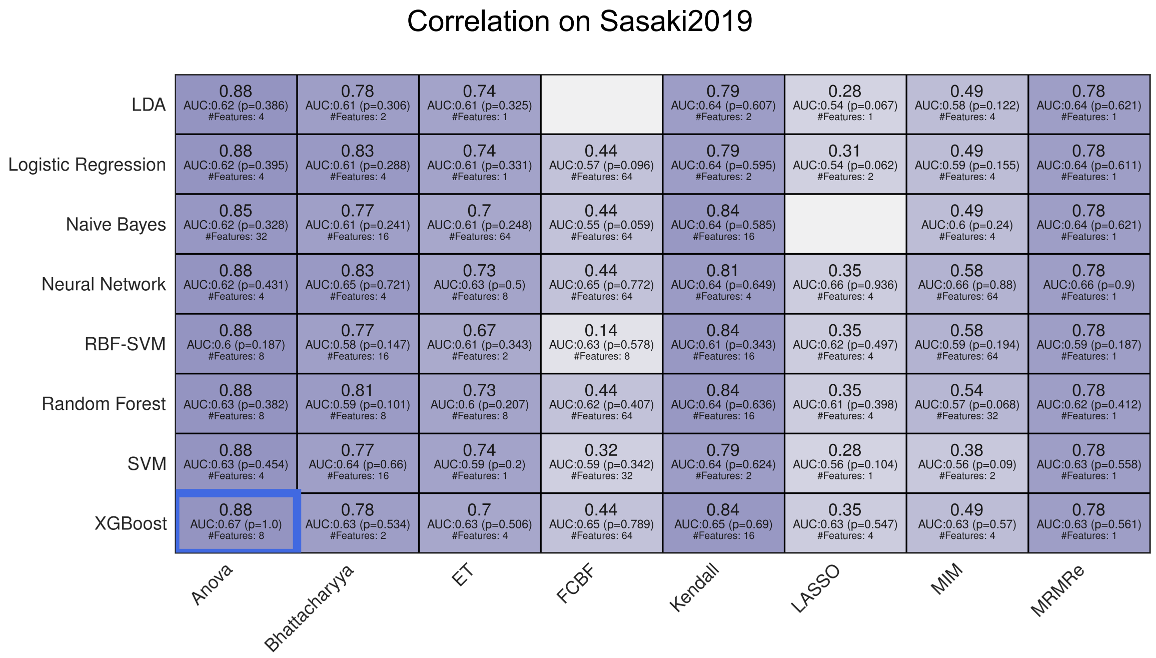

Figure S51: Correlation of the models on dataset Sasaki2019. The best model is framed with a blue border, models that were significantly different to the best model are not shown. Statistical significance was tested using a DeLong test.

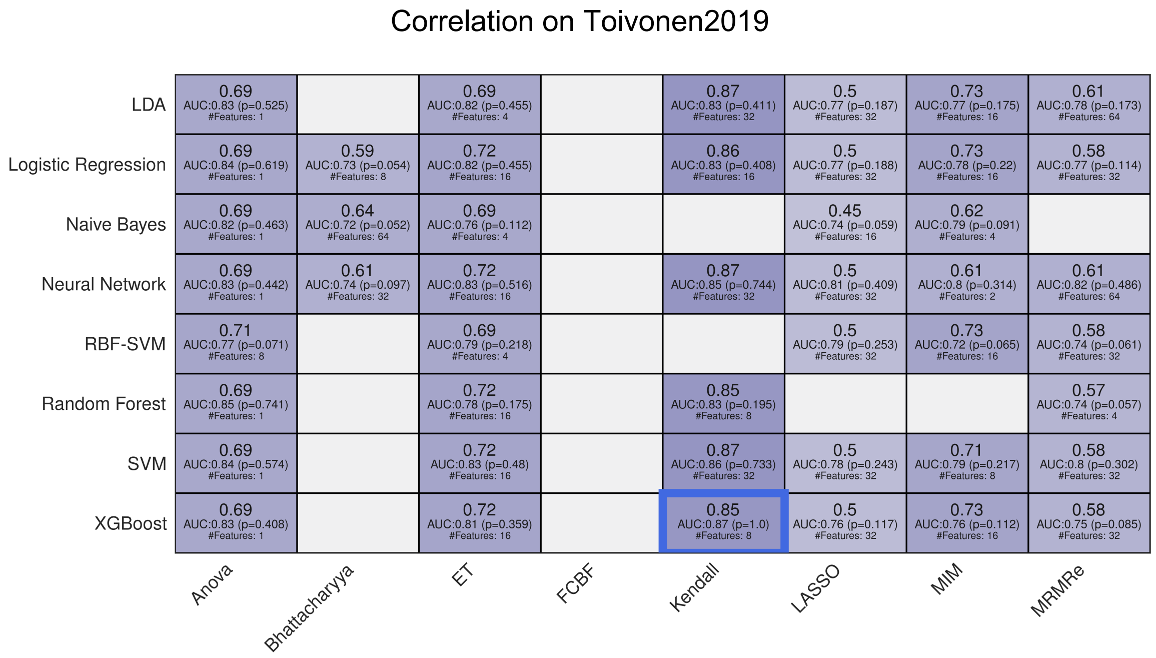

Figure S52: Correlation of the models on dataset Toivonen2019. The best model is framed with a blue border, models that were significantly different to the best model are not shown. Statistical significance was tested using a DeLong test.

Correlation on Keek2020

	Anova	Bhattacharyya	ET	FCBF	Kendall	LASSO	MIM	MRMRe
LDA	0.59<br>AUC:0.65 (p=0.137)<br>#Features: 4	0.57<br>AUC:0.64 (p=0.117)<br>#Features: 1	0.59<br>AUC:0.65 (p=0.183)<br>#Features: 2		0.56<br>AUC:0.65 (p=0.191)<br>#Features: 1		0.59<br>AUC:0.68 (p=0.709)<br>#Features: 8	0.34<br>AUC:0.64 (p=0.109)<br>#Features: 8
Logistic Regression	0.61<br>AUC:0.66 (p=0.233)<br>#Features: 16	0.63<br>AUC:0.66 (p=0.27)<br>#Features: 64	0.61<br>AUC:0.66 (p=0.242)<br>#Features: 8	0.59<br>AUC:0.65 (p=0.126)<br>#Features: 64	0.56<br>AUC:0.65 (p=0.185)<br>#Features: 1	0.59<br>AUC:0.68 (p=0.145)<br>#Features: 2	0.59<br>AUC:0.68 (p=0.787)<br>#Features: 8	0.34<br>AUC:0.64 (p=0.147)<br>#Features: 8
Naive Bayes	0.62<br>AUC:0.65 (p=0.225)<br>#Features: 32	0.63<br>AUC:0.66 (p=0.356)<br>#Features: 64	0.65<br>AUC:0.66 (p=0.299)<br>#Features: 32	0.59<br>AUC:0.64 (p=0.154)<br>#Features: 64	0.69<br>AUC:0.65 (p=0.151)<br>#Features: 64	0.6<br>AUC:0.65 (p=0.137)<br>#Features: 4	0.59<br>AUC:0.67 (p=0.629)<br>#Features: 8	
Neural Network	0.59<br>AUC:0.66 (p=0.338)<br>#Features: 4	0.57<br>AUC:0.65 (p=0.213)<br>#Features: 1	0.56<br>AUC:0.65 (p=0.134)<br>#Features: 1		0.65<br>AUC:0.65 (p=0.195)<br>#Features: 16	0.59<br>AUC:0.68 (p=0.56)<br>#Features: 2	0.59<br>AUC:0.67 (p=0.655)<br>#Features: 8	0.43<br>AUC:0.64 (p=0.21)<br>#Features: 32
RBF-SVM	0.63<br>AUC:0.64 (p=0.063)<br>#Features: 64	0.57<br>AUC:0.65 (p=0.213)<br>#Features: 1	0.67<br>AUC:0.63 (p=0.054)<br>#Features: 64	0.59<br>AUC:0.63 (p=0.053)<br>#Features: 64	0.61<br>AUC:0.65 (p=0.153)<br>#Features: 4	0.59<br>AUC:0.66 (p=0.107)<br>#Features: 2	0.59<br>AUC:0.66 (p=0.403)<br>#Features: 8	0.51<br>AUC:0.67 (p=0.459)<br>#Features: 64
Random Forest	0.63<br>AUC:0.64 (p=0.18)<br>#Features: 64	0.63<br>AUC:0.67 (p=0.466)<br>#Features: 64	0.61<br>AUC:0.64 (p=0.168)<br>#Features: 8		0.69<br>AUC:0.66 (p=0.441)<br>#Features: 64	0.66<br>AUC:0.64 (p=0.074)<br>#Features: 32	0.64<br>AUC:0.64 (p=0.118)<br>#Features: 32	0.51<br>AUC:0.68 (p=0.725)<br>#Features: 64
SVM	0.61<br>AUC:0.66 (p=0.374)<br>#Features: 16	0.57<br>AUC:0.65 (p=0.143)<br>#Features: 1	0.59<br>AUC:0.65 (p=0.247)<br>#Features: 2		0.61<br>AUC:0.65 (p=0.221)<br>#Features: 4	0.59<br>AUC:0.69 (p=1.0)<br>#Features: 2	0.59<br>AUC:0.68 (p=0.715)<br>#Features: 8	0.34<br>AUC:0.64 (p=0.162)<br>#Features: 8
XGBoost	0.61<br>AUC:0.65 (p=0.28)<br>#Features: 16	0.6<br>AUC:0.63 (p=0.132)<br>#Features: 16	0.61<br>AUC:0.65 (p=0.305)<br>#Features: 8		0.63<br>AUC:0.65 (p=0.236)<br>#Features: 8	0.66<br>AUC:0.63 (p=0.069)<br>#Features: 32	0.64<br>AUC:0.66 (p=0.445)<br>#Features: 32	0.51<br>AUC:0.64 (p=0.156)<br>#Features: 64

Figure S53: Correlation of the models on dataset Keek2020. The best model is framed with a blue border, models that were significantly different to the best model are not shown. Statistical significance was tested using a DeLong test.

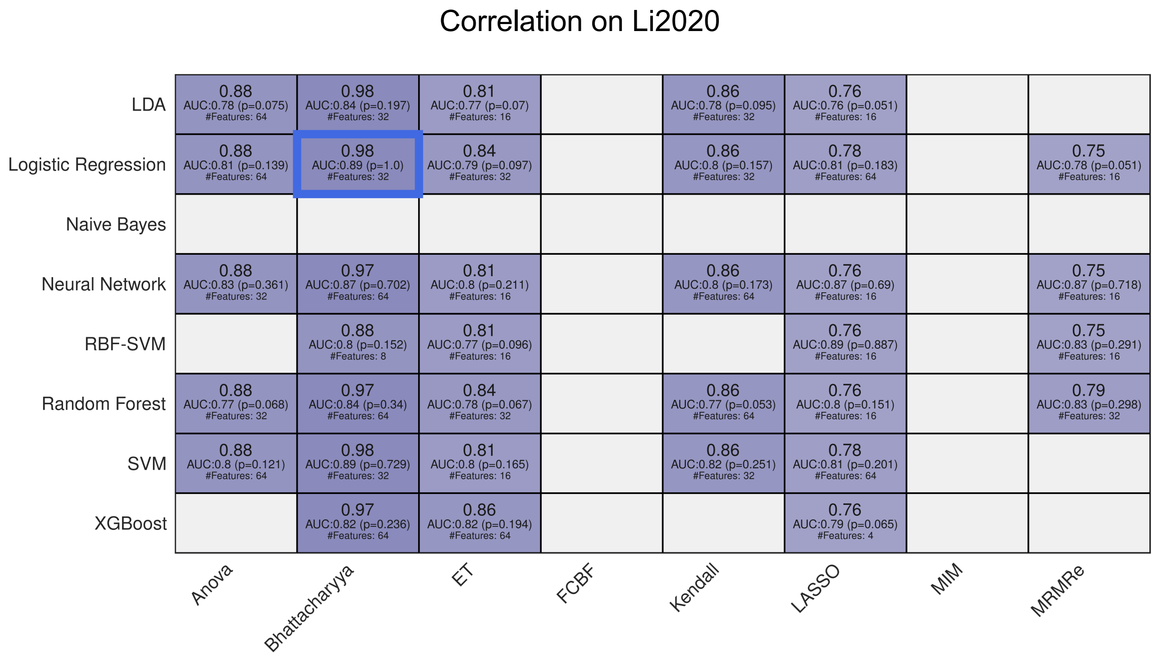

Figure S54: Correlation of the models on dataset Li2020. The best model is framed with a blue border, models that were significantly different to the best model are not shown. Statistical significance was tested using a DeLong test.

Correlation on Park2020

	Anova	Bhattacharyya	ET	FCBF	Kendall	LASSO	MIM	MRMRe
LDA	0.98 AUC:0.64 (p=0.443) #Features: 64	0.95 AUC:0.63 (p=0.22) #Features: 32	0.8 AUC:0.62 (p=0.144) #Features: 32	0.75 AUC:0.62 (p=0.113) #Features: 64	0.97 AUC:0.63 (p=0.199) #Features: 64	0.77 AUC:0.63 (p=0.364) #Features: 32	0.79 AUC:0.62 (p=0.154) #Features: 64	0.59 AUC:0.61 (p=0.051) #Features: 64
Logistic Regression	0.98 AUC:0.64 (p=0.509) #Features: 64	0.97 AUC:0.64 (p=0.617) #Features: 64	0.8 AUC:0.62 (p=0.147) #Features: 64	0.75 AUC:0.62 (p=0.125) #Features: 64	0.97 AUC:0.63 (p=0.292) #Features: 64	0.75 AUC:0.64 (p=0.631) #Features: 64	0.79 AUC:0.63 (p=0.209) #Features: 64	0.59 AUC:0.61 (p=0.062) #Features: 64
Naive Bayes	0.98 AUC:0.62 (p=0.092) #Features: 64	0.9 AUC:0.62 (p=0.083) #Features: 16		0.75 AUC:0.61 (p=0.096) #Features: 64	0.97 AUC:0.62 (p=0.137) #Features: 64		0.78 AUC:0.61 (p=0.068) #Features: 32	
Neural Network	0.98 AUC:0.65 (p=1.0) #Features: 64	0.83 AUC:0.62 (p=0.143) #Features: 4	0.8 AUC:0.63 (p=0.414) #Features: 64	0.67 AUC:0.61 (p=0.091) #Features: 32	0.97 AUC:0.63 (p=0.385) #Features: 64	0.75 AUC:0.64 (p=0.741) #Features: 64	0.79 AUC:0.63 (p=0.483) #Features: 64	
RBF-SVM	0.98 AUC:0.62 (p=0.229) #Features: 64			0.75 AUC:0.6 (p=0.073) #Features: 64	0.97 AUC:0.61 (p=0.079) #Features: 64	0.77 AUC:0.61 (p=0.142) #Features: 32		
Random Forest	0.98 AUC:0.61 (p=0.079) #Features: 64	0.97 AUC:0.61 (p=0.079) #Features: 64				0.77 AUC:0.61 (p=0.063) #Features: 32	0.79 AUC:0.61 (p=0.061) #Features: 64	
SVM	0.98 AUC:0.64 (p=0.374) #Features: 64	0.97 AUC:0.63 (p=0.24) #Features: 64	0.8 AUC:0.61 (p=0.068) #Features: 32	0.75 AUC:0.61 (p=0.088) #Features: 64	0.97 AUC:0.64 (p=0.325) #Features: 64	0.77 AUC:0.63 (p=0.351) #Features: 32	0.79 AUC:0.62 (p=0.263) #Features: 64	
XGBoost	0.98 AUC:0.62 (p=0.232) #Features: 64	0.97 AUC:0.62 (p=0.217) #Features: 64		0.75 AUC:0.62 (p=0.227) #Features: 64	0.97 AUC:0.62 (p=0.191) #Features: 64	0.75 AUC:0.63 (p=0.296) #Features: 64	0.79 AUC:0.6 (p=0.051) #Features: 64	

Figure S55: Correlation of the models on dataset Park2020. The best model is framed with a blue border, models that were significantly different to the best model are not shown. Statistical significance was tested using a DeLong test.

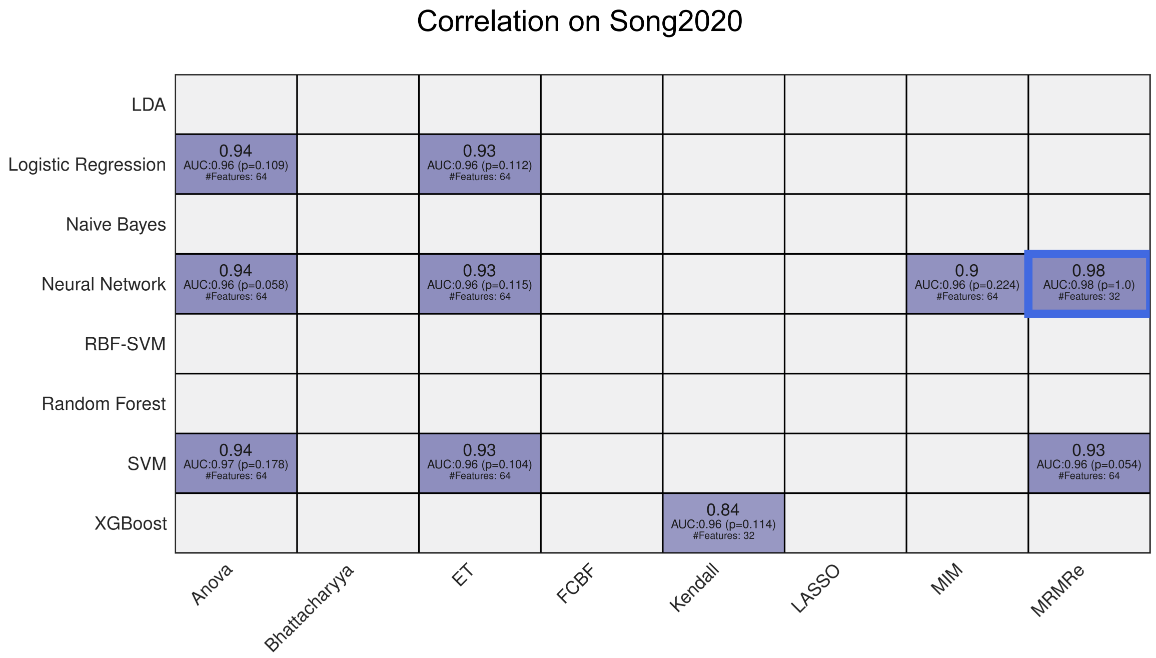

Figure S56: Correlation of the models on dataset Song2020. The best model is framed with a blue border, models that were significantly different to the best model are not shown. Statistical significance was tested using a DeLong test.

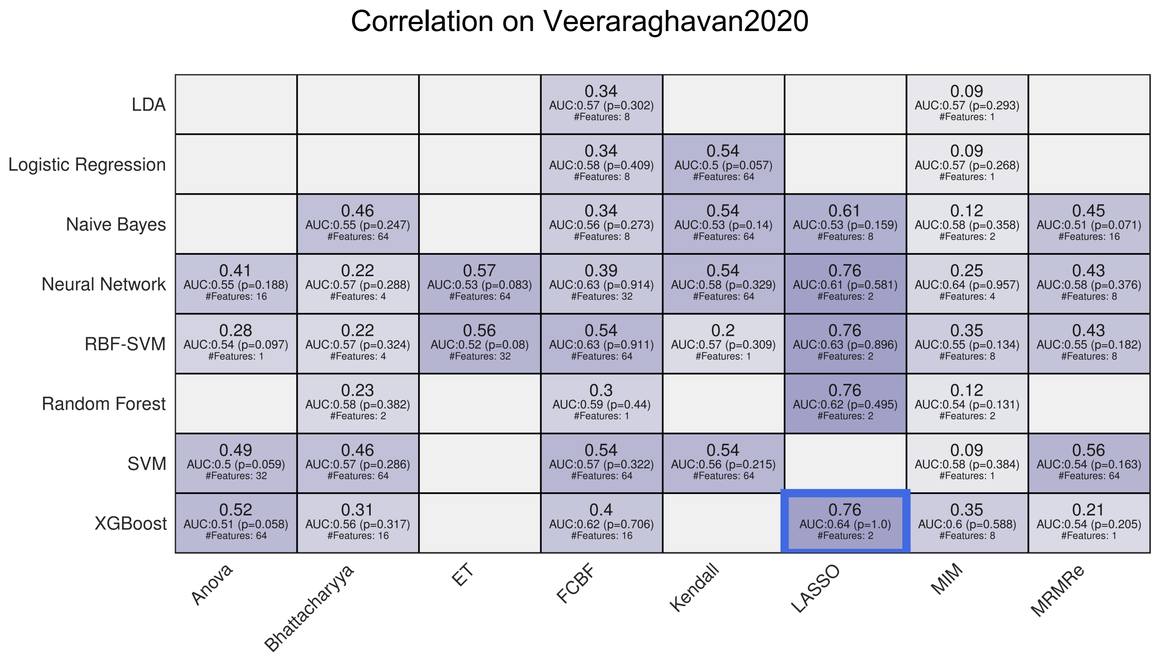

Figure S57: Correlation of the models on dataset Veeraraghavan2020. The best model is framed with a blue border, models that were significantly different to the best model are not shown. Statistical significance was tested using a DeLong test.
